# *Endo*-functionalization of tailored-adaptive nanospace for efficient binding of organic molecules in non-aqueous media

**DOI:** 10.1093/nsr/nwaf296

**Published:** 2025-07-21

**Authors:** Zi-En Zhang, Le Zhang, Lu-Wen Zhang, Ying-Feng Han

**Affiliations:** Key Laboratory of Synthetic and Natural Functional Molecule of the Ministry of Education, College of Chemistry and Materials Science, Northwest University, Xi'an 710127, China; Key Laboratory of Synthetic and Natural Functional Molecule of the Ministry of Education, College of Chemistry and Materials Science, Northwest University, Xi'an 710127, China; Key Laboratory of Synthetic and Natural Functional Molecule of the Ministry of Education, College of Chemistry and Materials Science, Northwest University, Xi'an 710127, China; Key Laboratory of Synthetic and Natural Functional Molecule of the Ministry of Education, College of Chemistry and Materials Science, Northwest University, Xi'an 710127, China; State Key Laboratory of Coordination Chemistry, School of Chemistry and Chemical Engineering, Nanjing University, Nanjing 210093, China

**Keywords:** adaptive nanospace, coordination cage, *endo*-functionalization, host–guest chemistry

## Abstract

Precision in controlling the microenvironment of nanospaces is a potent strategy for exploring architecture‒function relationships. Herein, a face-capped tetrahedral cage, featuring Pd‒Pd-bonded vertices, with a tailored nanospace surrounded by 12 ethyl units, was facilitated to adaptively accommodate a library of guests with different sizes and shapes, including C6 cyclic hydrocarbons, adamantane derivatives, S_8_ and P_4_. This nanocavity can achieve strong binding with cyclohexane in non-aqueous media in contrast to reported structurally similar non-*endo*-functionalized cages by an increase of four orders of magnitude. The crystal structures of free cage and host‒guest complexes demonstrate that the aliphatic units within the nanospace are allowed to adaptively deform to stabilize the guests or serve as grippers to form unique interactions. This work indicates the achievability of both efficient organic guests and elemental sulfur/phosphorus binding in a non-aqueous system with minimal synthetic efforts, by modifying inward-facing aliphatic units to the inner face of the nanospace.

## Teaser

A tetrahedral cage with palladium-palladium-bonded nodes has a tailored confined nanospace surrounded by 12 flexible aliphatic grippers, which can efficiently and adaptively trap different guests with enhanced affinity.

## INTRODUCTION

The dynamic process accompanying the adaptive entrapment of guests within a microenvironment holds significant importance in biological systems [1]. Drawing inspiration from biological superstructures, the microenvironment of coordination cages is characterized by confined nanospaces and formed by coordination-driven self-assembly; they find diverse applications in guest binding and sensing, entrapping active species and catalyzing reactions [2–9]. During the recognition process, these nanospaces undergo adaptive conformational adjustments based on the shape of guests, facilitating optimal matching and ensuring the highest affinity [10–13]. Notably, most coordination cages with well-defined nanospaces are constructed using rigid building linkers to ensure the directionality of the coordination process, while flexible hosts often lack the capability to encapsulate guests owing to cavity collapse [14–20]. However, the strict rigidity that their architectures imposes has caused difficulties in deformation, limiting the adaptive capacity of the nanospaces to match the shape of guests [21–23]. Consequently, designing and constructing a coordination cage with an effectively deformable nanospace that allows dynamic motion while preventing architectural collapse presents a formidable challenge.

**Figure 1.**
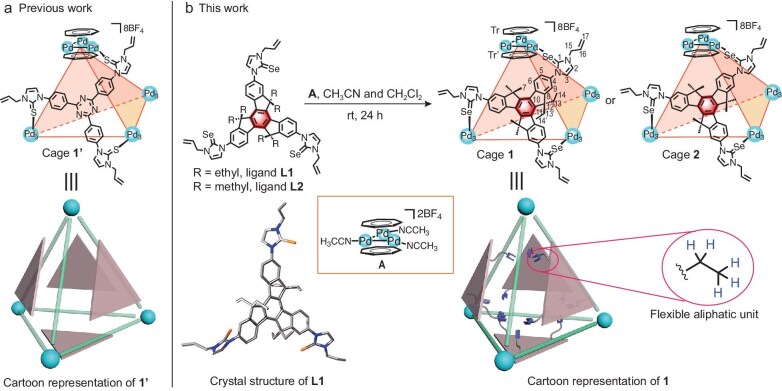
(a) Previous reported tetrahedral cage **1**′ with metal‒metal-bonded units and its cartoon representation. (b) Synthesis of *endo*-functionalized tetrahedral cages **1** and **2**. Insert: crystal structure of **L1** and cartoon representation of cage **1**. C, gray; N, blue; Se, light orange.

Triggering coordination-driven self-assembly with pre-functionalized organic linkers offers promising opportunities for constructing a range of *endo*-functionalized coordination cages [14–20,24–29]. In this category, the confined nanospace is modified by internal functional groups, mirroring the generic characteristics of the microenvironment found in biological receptors [30,31]. This intriguing approach allows precise construction and adjustment of the inner topology of the cage, and the interesting arrangement of specific functionalized units within the nanocavity induces changes in the local surroundings, enhancing adaptability to guests in non-aqueous media [32–34]. Obviously, the embrace created by these internally positioned functional sites is essential in determining the guest-binding properties of nanospaces [35–38]. Recently, attention has been drawn to coordination cages based on metal‒metal-bonded nodes due to their unique architectures for the selective binding and separation of specific guests in the aqueous phase [39–42]. Nevertheless, so far, these intriguing structures, which have been confined to inherent nanospaces surrounded by rigid aromatic panels, have been limited in their further development and applications of efficient host/guest partners in non-aqueous media due to their low affinity for organic molecules (Fig. 1a). Therefore, the *endo*-functionalization of such coordination cages holds the potential to introduce new functions and phenomena, thereby improving the guest-binding process.

Herein, we envision utilizing predesigned ligands containing the alkylated truxene core to initiate self-assembly with cycloheptatrienyl tripalladium complex **A** (a Pd‒Pd-bonded cluster in which the three metal centers are coordinated with the sp^2^ carbon atoms of two cycloheptatrienyl rings), resulting in the formation of *endo*-functionalized face-capped tetrahedral cages **1** and **2** (Fig. 1). The formed cavity of cage **1** incorporated 12 inward-oriented alkylated units (Fig. 1b). We anticipate that multiple flexible alkylated units within the nanospace can collectively and efficiently ensnare different guests. These inner units may deform the confined nanospace for accommodating the guests with different shapes and sizes, inducing enhanced affinity during the binding process. In fact, cage **1** can be tailored to encapsulate a wide variety of guests, including C6 cyclic hydrocarbons, adamantane derivatives, S_8_ and P_4_ in non-aqueous systems. For example, cage **1** with inward-oriented ethyl units exhibits a four-orders-of-magnitude improvement in cyclohexane binding in the organic phase (up to 2.06 × 10^5^ M^−1^) compared to reported structurally similar non-*endo*-functionalized cage **1**′ (Fig. 1a), as well as methyl-involving cage **2** [42]. Similar affinity enhancement was also observed for other guest molecules. X-ray crystallographic analysis of the host‒guest complexes confirmed that the guest-adaptive cavity breathing can be achieved through the conformational deformation of the microenvironment of the nanospace. The inner aliphatic units facilitate deformation to accommodate the guests and thus serve as grippers to form non-covalent interactions, potentially thereby inducing a strong affinity during the binding process. This work provides a valuable dimension of coordination cage to its functionality in terms of guest encapsulation.

## RESULTS AND DISCUSSION

### Ligand design and synthesis

Two truxene-core ligands **L1** and **L2** were designed and synthesized in three steps from hexaethyltruxene- or hexamethyltruxene-based starting materials (Figs S1‒S4). All intermediates and ligands were thoroughly characterized using NMR spectroscopy and high-resolution electrospray ionization (HR-ESI) mass spectrometry (Figs S5‒S19). Single-crystal X-ray diffraction analysis revealed a distinctive solid-state structure of ligand **L1**. Six sets of ethyl units were designed to be distributed almost perpendicularly to the central plane (Fig. 1b). The lengths of these ethyl units were constrained within the range of 2.54‒2.60 Å. This structural feature suggests that half of these aliphatic units may be directionally filled into the confined spaces after assembly, contributing to the overall understanding of the behavior of the ligand in the constructed coordination cage.

### Construction of *endo*-functionalized cages

**Figure 2.**
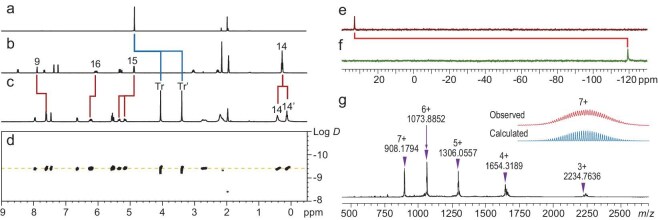
^1^H NMR spectra (CD_3_CN, 400 MHz, 298 K) of (a) complex **A**, (b) ligand **L1** and (c) cage **1**. (d) ^1^H DOSY (CD_3_CN, 400 MHz) spectrum of cage **1** (log *D* = −9.38). ^77^Se NMR spectra (CD_3_CN, 114 MHz, 298 K) of (e) ligand **L1** and (f) cage **1**. (g) HR-ESI mass spectrum (positive ions) of cage **1** and isotope distributions of selected cations [**1** ‒ 7BF_4_]^7+^ (experimentally observed distributions in red and calculated distributions in blue).

The reaction of ligand **L1** with complex **A** in a 1:1 mixture of acetonitrile and dichloromethane afforded face-capped tetrahedral cage **1** with triangular metal‒metal-bonded building blocks (Fig. 1b). Cage **1** was obtained in 96% yield and was comprehensively characterized using various techniques, including NMR spectroscopy (^1^H, ^13^C{^1^H}, ^19^F, ^77^Se and 2D NMR), as well as HR-ESI mass spectrometry (Fig. 2 and Figs S20‒S27). Following assembly, the ^1^H NMR spectrum of cage **1** revealed the proton signals assigned to the cycloheptatrienyl cationic rings underwent upfield shifts, which split into two sharp singlet signals. Similarly, the proton signals assigned to the ethyl units of truxene cores also split evenly into two sets of signals, reflecting differences in the chemical environment inside and outside the cavity of cage **1** (Fig. 2a‒c). The diffusion-ordered NMR spectroscopy (DOSY) experiment of cage **1** displayed a single band with a diffusion coefficient of *D* = 4.17 × 10^−10^ m^2^ s^−1^ (log *D* = −9.38), confirming the hydrodynamic radius was calculated to be 15.8 Å. (Fig. 2d).

Subsequently, ^77^Se NMR experiments were performed on ligand **L1** and Pd‒Pd-bonded cage **1** to investigate variations in the chemical environment of coordinated atoms. As shown in Fig. 2e and f, upon the assembly of ligand **L1** with Pd–Pd-bonded clusters, the sharp singlet (*δ* = 33.03 ppm) assigned to Se atoms of **L1** experienced a substantial upfield shift to *δ* = −119.27 ppm (Δ*δ* = 152.30 ppm). This shift indicates the formation of a new species after the assembly process. The ^77^Se NMR results offer valuable insights into the chemical transformation during the assembly of ligand **L1** into Pd‒Pd-bonded cage **1**, revealing changes in the electronic environment surrounding selenium atoms during the self-assembly process.

The formation of cage **1** was unambiguously supported by the presence of five major peaks corresponding to [**1** ‒ *n*BF_4_]*^n^*^+^ (*n* = 3‒7) in the HR-ESI mass spectrum (positive ion mode) (Fig. 2g and Fig. S27). In addition, the peaks that belong to {[**1** ‒ 4BF_4_] + CH_3_CN}^4+^ and {[**1** ‒ 3BF_4_] + CH_3_CN}^3+^ were also observed.

Similarly, cage **2** was also synthesized from ligand **L2** and complex **A** in high yield (Fig. 1b). It was fully characterized by multiple NMR spectroscopy and HR-ESI mass spectrometry (Figs S28‒S36). Again, ^77^Se NMR spectra showed the sharp singlet assigned to **L2** (*δ* = 27.55 ppm) experienced significant upfield shift (Δ*δ* = 132.82 ppm) after assembly, reaching *δ* = −105.27 ppm (Fig. S36).

**Figure 3.**
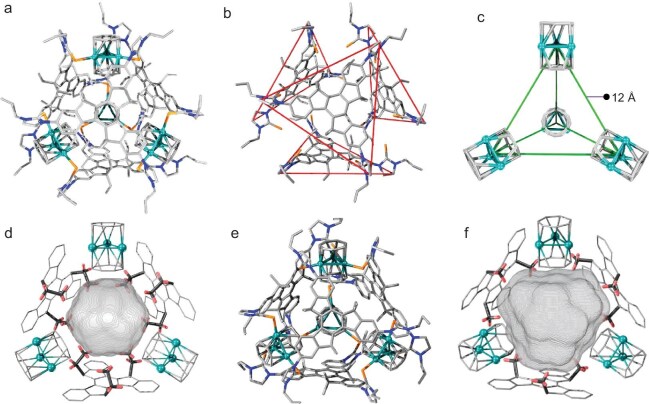
(a) Cationic part of the crystal structure of cage **1** confirmed by single-crystal X-ray diffraction. (b) View of the four organic ligands of cage **1**. (c) View of the four tripalladium fragments within cage **1**. (d) View of the partial aliphatic units oriented towards the interior of cage **1** and its cavity as calculated using VOIDOO. (e) Cationic part of the crystal structure of cage **1** confirmed by single-crystal X-ray diffraction. (f) View of the partial aliphatic units oriented towards the interior of cage **2** and its cavity as calculated using VOIDOO. Pd, teal sphere; Se, light orange; N, blue; C, gray; H, light red. Hydrogen atoms have been omitted for clarity (except for partial ethyl units oriented inwards the cavity).

The composition of cage **1** was further elucidated using single-crystal X-ray diffraction analysis. Single crystals suitable for diffraction were obtained by the slow vapor diffusion of isopropyl ether into a *N,N*-dimethylformamide (DMF) solution of cage **1**. The resulting crystal structure revealed the presence of four Pd–Pd-bonded clusters bridged by four ligands, giving rise to a slightly deformed tetrahedral vessel (Fig. 3a). In this structure, each Pd–Pd-bonded cluster occupied a vertex of the cage, and each functionalized ligand formed a triangular ‘shell’ surrounding a tetrahedral surface (Fig. 3b). Four inward cycloheptatrienyl cationic rings enclosed a smaller tetrahedral part, whereas the remaining four rings were positioned outside the cavity (Fig. 3c). The Pd–Pd-bonded cores were separated by distances of about 12 Å. Intriguingly, the 12 sets of ethyl units of the four truxene centers were directionally filled into the cavity, while the other 12 sets of ethyl units pointed outward. This strategy of continuously compressing the space of the cavity with aliphatic units endows it with a novel internal environment involving flexibility and aliphatic characteristics, distinguishing it from common cages equipped with lined aromatic panels. Ligand **L1** and tripalladium clusters were anchored through Pd‒Se-based coordination interactions, with bond distances ranging from 2.568(7) to 2.583(0) Å. The Pd–Pd bond lengths fell within the range of 2.785(7)‒2.791(7) Å, while the Pd–C_Tr_/C_Tr__′_ bond lengths were consistent with those reported in previous work [42]. The lipophilic cavity volume of cage **1**, enclosed by the inner 12 ethyl units and four cycloheptatrienyl cationic rings, was calculated to be 272 Å^3^ using VOIDOO (Fig. 3d).

The crystal structure of cage **2** also exhibited a very similar deformed tetrahedral container (Fig. 3e and Fig S37). However, the flexibility of the nanospace decreased due to the replacement of flexible ethyl units with rigid methyl units, and the volume of the cavity increased (Fig. 3f).

### Guest-binding properties

As observed in the single-crystal structures (Fig. 3), 12 ethyl units in a confined space provide an additional inner microenvironment distinct from reported non-*endo*-functionalized cages [39–42]. We hypothesize that the flexible alkyl chains within the cavity may facilitate deformation to adapt to the shape and size of the guest, leading to strong affinity during the binding process [36].

**Figure 4.**
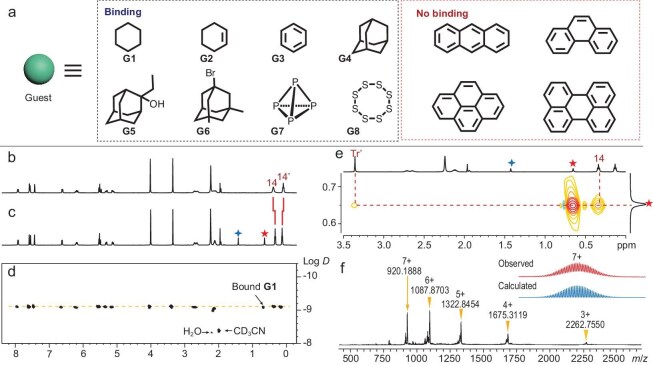
(a) Summary of the host‒guest chemistry of cage **1** in CD_3_CN solution. ^1^H NMR spectra (CD_3_CN, 400 MHz, 298 K) of (b) free cage **1** and (c) cage **1** binding **G1**. (d) ^1^H DOSY spectrum (CD_3_CN, 400 MHz, 298 K) of cage **1** binding **G1** (log *D* = −9.13). (e) ^1^H–^1^H NOESY spectrum (CD_3_CN, 600 MHz, 298 K) of cage **1** binding **G1**. (f) HR-ESI mass spectrum (positive ions) of cage **1** binding **G1** and isotope distributions of selected cations [**G1** **⊂** **1** ‒ 7BF_4_]^7+^ (experimentally observed distributions in red and calculated distributions in blue). Red five-pointed star, bound guest; blue four-pointed star, free guest.

Firstly, the binding studies of cage **1** with C6 cyclic hydrocarbons **G1‒G3** were investigated (Fig. 4a). These guests can be encapsulated in cage **1**, and all host‒guest complexes were fully characterized by NMR spectroscopy (^1^H, ^1^H‒^1^H NOESY and ^1^H DOSY NMR) and HR-ESI mass spectrometry (Figs S38‒S47). For example, when excess **G1** was added to the acetonitrile solution of cage **1**, in addition to discovering the proton signal (*δ* = 1.43 ppm) assigned to free **G1**, we also observed a sharp resonance signal (*δ* = 0.65 ppm) assigned to bound **G1**, indicating slow exchange of guest binding on the NMR time scale (Fig. 4b and c). This obvious upfield shift (Δ*δ* = 0.78 ppm) was attributed to the strong shielding effect of cage **1**. Inconspicuous shifts in the proton signal of the host (Δ*δ* ≤ 0.05 ppm) were observed, indicating a close match between the guest and the confined nanospace [41]. The DOSY experiment showed that the diffusion coefficient of encapsulated **G1** was consistent with that of the complexed cage **1** (log *D* = −9.13), further indicating the formation of the host–guest complex (Fig. 4d). In the ^1^H‒^1^H NOESY spectrum of **G1** **⊂** **1**, strong nuclear Overhauser effect (NOE) cross peaks were found between the proton signals of bound **G1** and the proton signals H14 of the ethyl units, as well as the proton signals H_Tr__′_ of the internal cycloheptatrienyl cations, respectively (Fig. 4e). These results confirmed the existence of close through-space contacts between cage **1** and **G1**.

Furthermore, the HR-ESI mass spectrum (positive ion mode) of **G1** **⊂** **1** indicated a series of major peaks corresponding to [**G1** **⊂** **1** ‒*n*BF_4_]*^n^*^+^ (*n* = 3‒7), which fit perfectly with the theoretical isotopic distribution. For example, the measured peak for [**G1** **⊂** **1** ‒ 7BF_4_]^7+^ at *m/z* = 920.1888 closely matched its theoretical distribution at *m/z* = 920.1931 (Fig. 4f and Fig. S39). Similarly, the addition of **G2** or **G3** into the solution of cage **1** also resulted in the formation of their corresponding inclusion complexes (Figs S40‒S47).

Titrations of guests **G1‒G3** into the CD_3_CN solution of cage **1** were investigated by ^1^H NMR spectroscopy, and the binding constants *K*_a_ for **G1‒G3** were obtained (Figs S48‒S55 and Table 1). In addition, the isothermal titration calorimetry (ITC) experiments were conducted to obtain a more accurate binding constant of cage **1** for **G1**, that cage **1** was observed to exhibit a remarkably strong affinity for cyclohexane (**G1**), achieving a *K*_a_ of up to (2.06 ± 0.40) × 10^5^ M^−1^ in CH_3_CN (Fig. S50). This affinity surpasses that of reported coordination cages for cyclohexane in the organic phase (Fig. S55) [42–47]. The Δ*H* and Δ*S* values were −8.6 kcal mol^−1^ and −4.4 cal mol^−1^ K^−1^, respectively (Fig. S50). Compared to cage **2** or non-*endo*-functionalized cage **1**′ with similar topologies, the affinity of **G1‒G3** has generally been enhanced by cage **1** (Table 1 and Figs S56‒S64) [42]. The observations indicate the flexible, inward-facing ethyl units within nanospace play an important role in encapsulating the guest.

**Table 1.** Binding constants *K*_a_ of cage **1**′, cage **2** and cage **1**, for C6 cyclic hydrocarbons **G1‒G3** determined by ^1^H NMR titrations in CD_3_CN at 298 K.

	*K*_a_ of cage **1**′ (M^−1^) ^a^	*K*_a_ of cage **2** (M^−1^)	*K*_a_ of cage **1** (M^−1^)
**G1**	8.9	(1.03 ± 0.02) × 10^1^	(2.06 ± 0.40) × 10^5^ ^b^
**G2**	5.8	(6.23 ± 0.09) × 10^0^	(8.12 ± 1.16) × 10^3^
**G3**	No binding	No binding	(3.03 ± 0.05) × 10^1^

a The values were taken from Wang *et al.* [42].

b The binding constant was determined by the ITC experiments in CH_3_CN at 298 K.

Although cage **1** only exhibited a low binding constant for aromatic guest **G3**, it is worth noting that non-*endo*- or methyl-functionalized cages do not trap benzene under the same conditions (Table 1 and Fig. S64) [42]. These results again indicate that the inner flexible aliphatic units play a crucial role in the affinity improvement of guest binding. We infer that the high affinity of cage **1** toward **G1** results from a better size and shape match together with multiple weak interactions with the nanospace, as compared to **G2** or **G3**.

Secondly, we examined the inclusion of adamantane and its derivatives with larger sizes and rigidity (Fig. 4). The inclusion species **G** **⊂** **1** (**G4, G5** or **G6**) were obtained when cage **1** was formed in the presence of adamantane and its derivatives, as detected by the ^1^H NMR spectra. ESI-MS experiments also confirmed the binding of one guest molecule to cage **1** (Figs S65‒S70).

Finally, we envisaged this tailored nanocavity to be suitable for the binding of elemental sulfur and white phosphorus (Fig. 4a) [18,48–50]. In fact, the addition of P_4_ (**G7**) and S_8_ (**G8**) to an acetonitrile solution of cage **1** resulted in the formation of **G7** **⊂** **1** and **G8** **⊂** **1** rapidly (Figs S71‒S74). In the ^31^P NMR spectra of cage **1** binding **G7**, the obvious upfield shift (Δ*δ* = 11.6 ppm) of the encapsulated P_4_ signal was attributed to the strong shielding effect of the cavity (Fig. S72) [13,18]. The ESI-MS spectrum of **G8** **⊂** **1** revealed multiple prominent peaks corresponding to one S_8_ encapsulated species with charge states resulting from the loss of the BF_4_ counterion (Fig. S74). The peaks corresponding to cage **1** complexed with one S_8_ molecule were found at *m*/*z* = 2320.0029, 1718.5342, 1357.4786, 1116.6879 and 944.7703, corresponding to [**G8** **⊂** **1** ‒ *n*BF_4_]*^n^*^+^ (*n* = 3, 4, 5, 6, 7), respectively. These peaks were isotopically resolved and agree very well with their calculated theoretical distributions. Furthermore, the addition of bromine leads to the dissociation of the architecture, thereby achieving the release of the guests.

**Figure 5.**
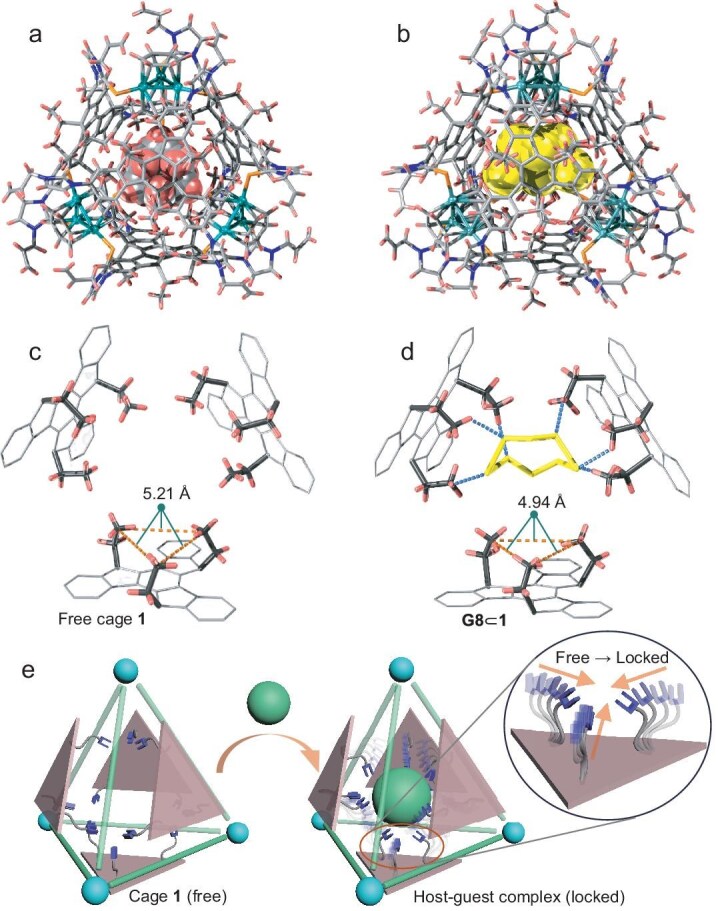
Cationic part of the crystal structure of (a) **G4** **⊂** **1** and (b) **G8** **⊂** **1** confirmed by single-crystal X-ray diffraction. Views involving partial aliphatic *endo*-functional units of a portion of the crystal structures of (c) free cage **1** and (d) **G8** **⊂** **1** confirmed by single-crystal X-ray diffraction, and CH···S interaction between the center panels and the S_8_ molecule. (e) Cartoon image of trapping the guest in cage **1**.

Fortunately, single crystals of cage **1** encapsulating **G4** and **G8** were obtained by the slow vapor diffusion of benzene into their corresponding DMF solutions (Fig. 5a and b). In the solid state, the structures demonstrate the flexibility of the aliphatic units inside the cavity, allowing adaptation to catch guests, although the distances between trimetallic metal–metal-bonded clusters did not change before and after accommodating guests (Fig. S75). In detail, compared to the distance between the inner ethyl vertices on the same central plane of free cage **1**, that of **G4** **⊂** **1** has significantly decreased from 5.21 to 4.86 Å, reflecting the conformational adjustment involving the adaptive guest-binding process (Fig. S76). Similarly, the distance between inner ethyl vertices of **G8** **⊂** **1** after host‒guest complexation has obviously decreased to 4.94 Å (Fig. 5c and d). In addition, the S_8_ molecule was strongly restricted by employing internal ethyl units as grippers to form CH···S interactions (2.49 Å). While guest encapsulation did not change the position of trimetallic vertices and the size of the tetrahedral cage, the shape and volume of the nanocavity were impacted by the positions of inner aliphatic units, which were influenced by the size and shape of the guests (Fig. 5e). Specifically, during the host‒guest complexation, the inner ethyl units have transitioned from a free mode to a locked mode with appropriate distance changes between each other. All in all, the presence of these aliphatic units serves as flexible grippers, allowing for adaptable deformation or providing weak interactions to accommodate different types of guests or provide high affinity. We acknowledge that the nanocavity expansion also possibly occurs through the motion of *endo*-functionalized aliphatic units to accept other special guests.

## CONCLUSION

In conclusion, we have constructed an *endo*-functionalized tetrahedral cage **1**, which incorporates triangular metal–metal-bonded coordination vertices and organic selenone ligands through rational design. By changing the shape and size of the nanocavity through the movement of internal functional units, the desired cage was tailored to adaptively encapsulate a wide variety of guests with different sizes and shapes, including C6 cyclic hydrocarbons, adamantane derivatives, S_8_ and P_4_. Cage **1**, containing 12 inward-facing ethyl grippers, exhibits the significant improvement in the binding of C6 cyclic hydrocarbons in the organic phase compared to other structurally similar non-*endo*- or methyl-functionalized cages. The special host‒guest property of cage **1** comes from its unique nanospace, in which the inner ethyl groups are partially flexible to adapt themselves to the size and shape of the encapsulated guest for conformational adjustment or to provide weak interactions as grippers, thereby achieving the ameliorative guest-binding process. This work offers significant reference value for the precise and effective construction and adjustment of the topology and the properties of the nanospace of coordination cages with metal–metal-bonded units. Additionally, the special bonding properties of such an inner cavity described herein may serve as the new hosts for separation, purification or stabilization of different species.

## METHODS

All details on syntheses, single-crystal structure determination and details in the guest-binding process are provided in the Supplementary data.

## Supplementary Material

nwaf296_Supplemental_File
