# Supplementary material for: Endo-functionalization of tailored-adaptive nanospace for efficient binding of organic molecules in non-aqueous media
Source: Natl Sci Rev. 2025 Jul 21;12(9):nwaf296. doi: 10.1093/nsr/nwaf296 (PMC12409780; doi:10.1093/nsr/nwaf296)
Supplement: nwaf296_Supplemental_File [file nwaf296_supplemental_file.pdf]

Supporting Information for

Endo-functionalization of tailored-adaptive nanospace for efficient binding of organic molecules in nonaqueous media

Zi-En Zhang[1], Le Zhang[1], Lu-Wen Zhang[1], Ying-Feng Han[1,2,]*

[1]Key Laboratory of Synthetic and Natural Functional Molecule of the Ministry of Education, College of Chemistry and Materials Science, Northwest University, Xi'an 710127, China.

[2]State Key Laboratory of Coordination Chemistry, School of Chemistry and Chemical Engineering, Nanjing University, Nanjing 210093, China.

*Corresponding authors: yfhan@nwu.edu.cn

Table of Contents

1. Materials and methods S3

2. Synthesis of **b1**, **c1**, **L1** and **L2** S4

   2a. Synthesis of **b1** S4

   2b. Synthesis of trisimidazolium salt **c** S5

   2c. Synthesis of ligand **L1** S6

   2d. Synthesis of ligand **L2** S7

3. Synthesis of cages **1** and **2** S9

   3a. Synthesis of cage **1** S9

   3b. Synthesis of cage **2** S10

4. NMR spectra and ESI-MS spectra S11

5. Volume calculations with VOIDOO S26

6. Hydrodynamic radius S27

7. Host–guest chemistry between cage **1** and C6 cyclic hydrocarbons S28

8. Binding strength studies S33

9. Host–guest chemistry between cage **1** and other guests S42

10. X-ray crystallography S47

11. References S54

1. Materials and methods

Compound **a1**, **a2** and complex **A** were prepared were prepared following procedures outlined in the literature [S1, S2]. Unless otherwise stated, all reagents were purchased from commercial sources and used as received. ^{1}H, ^{13}C{^{1}H}, ^{19}F, ^{77}Se and 2D NMR spectra were recorded on Bruker AVANCE III 400 or Bruker AVANCE III HD 600 spectrometers. ^{1}H and ^{13}C{^{1}H} NMR chemical shifts were reported relative to residual solvent peaks (^{1}H NMR: 1.94 ppm for CD_3CN, ^{13}C{^{1}H} NMR: 1.32 ppm, 118.26 ppm for CD_3CN). Mass spectra were recorded on a Micromass Quattro II triple-quadrupole mass spectrometer using electrospray ionization with a MassLynx operating system. Isothermal titration calorimetry (ITC) was carried out using a VP-ITC (Malvern) at 298 K, and computer fitting of the data were performed using the VP-ITC analyze software. Diffraction data was collected at 200–236 K with a Bruker APEX-II CCD diffractometer.

2. Synthesis of b1, c1, L1 and L2

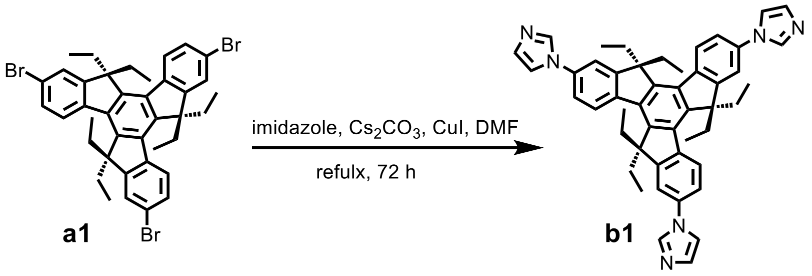

Figure S1. Synthesis of **b1**.

2a. Synthesis of b1.

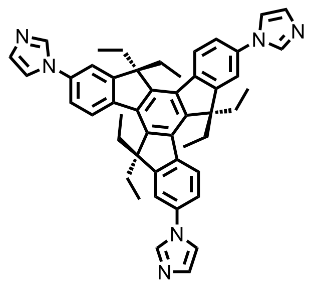

Synthetic route for **b1**: The compound **a1** (748.1 mg, 1.0 mmol), imidazole (1022.5 mg, 15.0 mmol), Cs_2CO_3 (4889.7 mg, 15.0 mmol) and CuI (571.4 mg, 3.0 mmol) were dissolved in anhydrous N,N-dimethylformamide (DMF, 40 mL) under a nitrogen atmosphere. The obtained mixture was refluxed for 72 h under exclusion of light. After cooling to room temperature, the solution was concentrated to 3 mL and H_2O (400 mL) was added to give a grayish green powder **b1**. The grayish green powder **b1** was collected by filtration, washed with H_2O (200 mL), and dried in *vacuo*. Yield: 594.9 mg (0.84 mmol, 84%). ^{1}H NMR (400 MHz, $CDCl_3$): δ = 8.42 (d, J = 9.0 Hz, 3H), 7.99 (s, 3H), 7.47−7.41 (m, 9H), 7.26 (s, 3H), 3.05−2.97 (m, 6H), 2.24−2.15 (m, 6H), 0.26 ppm (t, J = 7.2 Hz, 18H). ^{13}C{^{1}H} NMR (100 MHz, $CDCl_3$): δ = 154.3, 143.7, 138.8, 137.5, 135.6, 135.1, 129.9, 125.0, 119.0, 117.7, 114.5, 56.6, 28.9, 7.9 ppm. ESI-MS (positive ions): m/z = 709.3850 (calcd for [**b1** + H]$^+$ 709.4013).

2b. Synthesis of trisimidazolium salt c1.

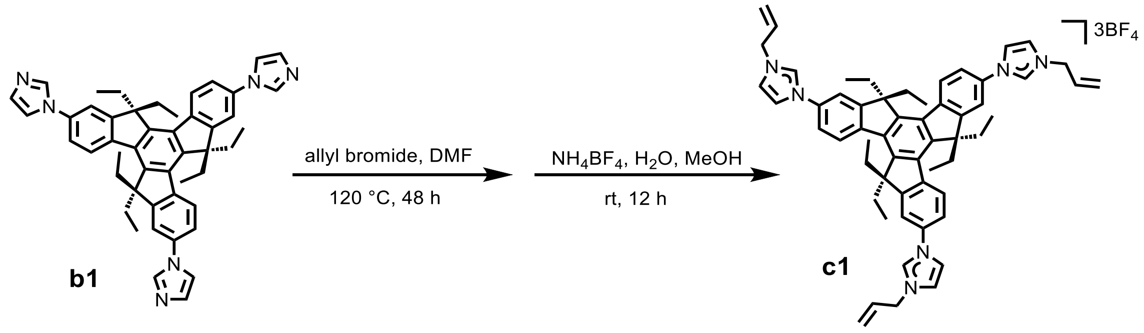

Figure S2. Synthesis of trisimidazolium salt **c1**.

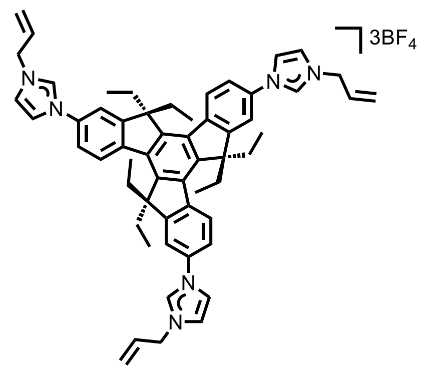

Synthetic route for **c1**: The C_3-symmetrical core **b1** (356.2 mg, 0.5 mmol) and allyl bromide (369.0 mg, 3.0 mmol) were dissolved in anhydrous N,N-dimethylformamide (DMF, 10 mL). The resulting mixture was heated at 120 ℃ for 48 h. After cooling to room temperature, the clear solution was concentrated to 2 mL, followed by the addition of ethyl acetate (20 mL), resulting in the precipitation of a brown solid. The solid was collected by filtration, washed with ethyl acetate (20 mL), and dried in *vacuo*. The obtained solid was dissolved in methanol (3 mL). Upon the addition of a solution of NH_4BF_4 (526.6 mg, 5.0 mmol) in H_2O (40 mL), a pale yellow precipitate formed immediately. The mixture was further stirred at room temperature for 12 h. The precipitate was collected by filtration, washed with H_2O, and dried in *vacuo* to afford trisimidazolium salt **c1**. Yield: 448.5 mg (0.41 mmol, 82%). ^{1}H NMR (400 MHz, CD_3CN): δ = 9.04 (s, 3H, $H_{N\text{-CH-}N}$), 8.60 (d, *J* = 8.6 Hz, 3H), 7.95 (s, 3H), 7.82 (s, 3H), 7.70 (d, *J* = 8.6 Hz, 3H), 7.63 (s, 3H), 6.20–6.09 (m, 3H), 5.55–5.52 (m, 6H), 4.91 (d, *J* = 5.8 Hz, 6H), 3.08–3.02 (m, 6H), 2.35–2.30 (m, 6H), 0.23 ppm (t, *J* = 5.8 Hz, 18H). ^{13}C{^{1}H} NMR (100 MHz, CD_3CN): δ = 156.1, 146.8, 142.2, 139.0, 134.9, 131.2, 127.0, 124.1, 124.1, 123.1, 122.5, 121.7, 121.7, 58.6, 53.0, 29.9, 8.8 ppm. ESI-MS (positive ions): *m/z* = 459.2546 (calcd for $[\textbf{c1} - 2BF_4]^{2+}$ 459.2571).

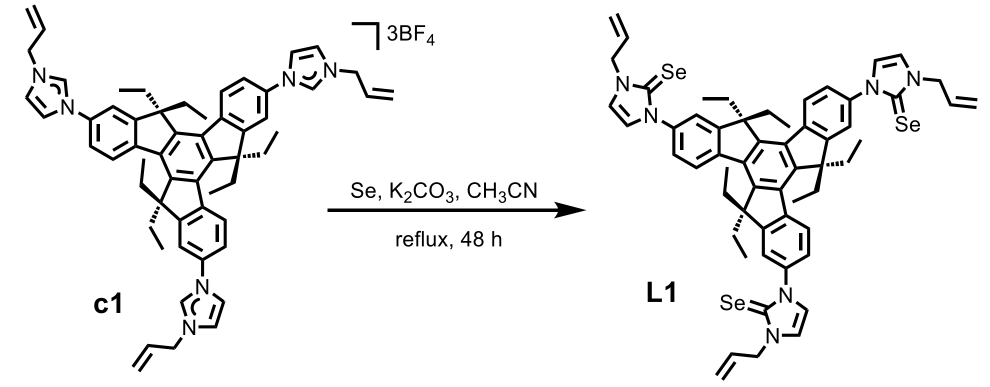

Figure S3. Synthesis of ligand **L1**.

2c. Synthesis of ligand L1.

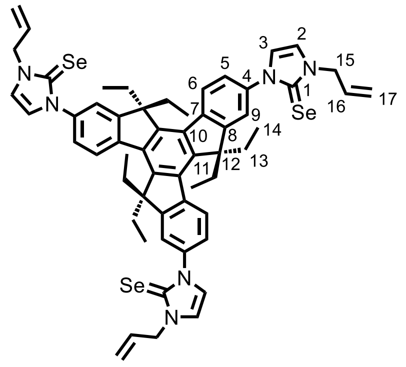

Synthetic route for ligand **L1**: The trisimidazolium salt **c1** (219.3 mg, 0.20 mmol), Se (96.2 mg, 1.20 mmol) and K_2CO_3 (278.0 mg, 2.00 mmol) were dissolved in acetonitrile (30 mL). The resulting mixture was refluxed for 48 h. After cooling to room temperature, the solution was evaporated to dryness and then extracted with CH_2Cl_2. The filtrate was washed with distilled water (3 × 50 mL). Afterwards, the solution was dried over $MgSO_4$. The clear solution was concentrated to 3 mL and diethyl ether (40 mL) was added to give a yellow solid **L1**. The yellow solid **L1** was collected by filtration, washed with diethyl ether, and dried in *vacuo*. Yield: 169.2 mg (0.16 mmol, 80%). ^{1}H NMR (400 MHz, $CDCl_3$): δ = 8.41 (d, *J* = 8.6 Hz, 3H, H6), 7.83 (d, *J* = 2.1 Hz, 3H, H9), 7.64 (dd, *J* = 8.6, 2.1 Hz, 3H, H5), 7.16 (d, *J* = 2.3 Hz, 3H, H3), 7.04 (d, *J* = 2.3 Hz, 3H, H2), 6.09−5.99 (m, 3H, H16), 5.39−5.35 (m ,6H, H17), 4.92 (d, *J* = 6.2 Hz, 6H, H15), 3.02−2.93 (m, 6H, H13), 2.25−2.17 (m, 6H, H13), 0.29 ppm (t, *J* = 7.2 Hz, 18H, H14). ^{13}C{^{1}H} NMR (100 MHz, $CDCl_3$): δ = 156.3 (C1), 153.1 (C8), 144.8 (C11), 139.7 (C7), 137.6 (C10), 136.9 (C4), 131.1 (C16), 124.4 (C6), 123.3 (C5), 120.3 (C9), 119.9 (C3), 119.3 (C17), 118.4 (C2), 56.6 (C12), 51.9 (C15), 28.7 (C13), 8.2 (C14) ppm. ESI-MS (positive ions): *m/z* = 1089.2453 (calcd for [**L1** + Na]$^+$ 1089.2296).

2d. Synthesis of ligand L2.

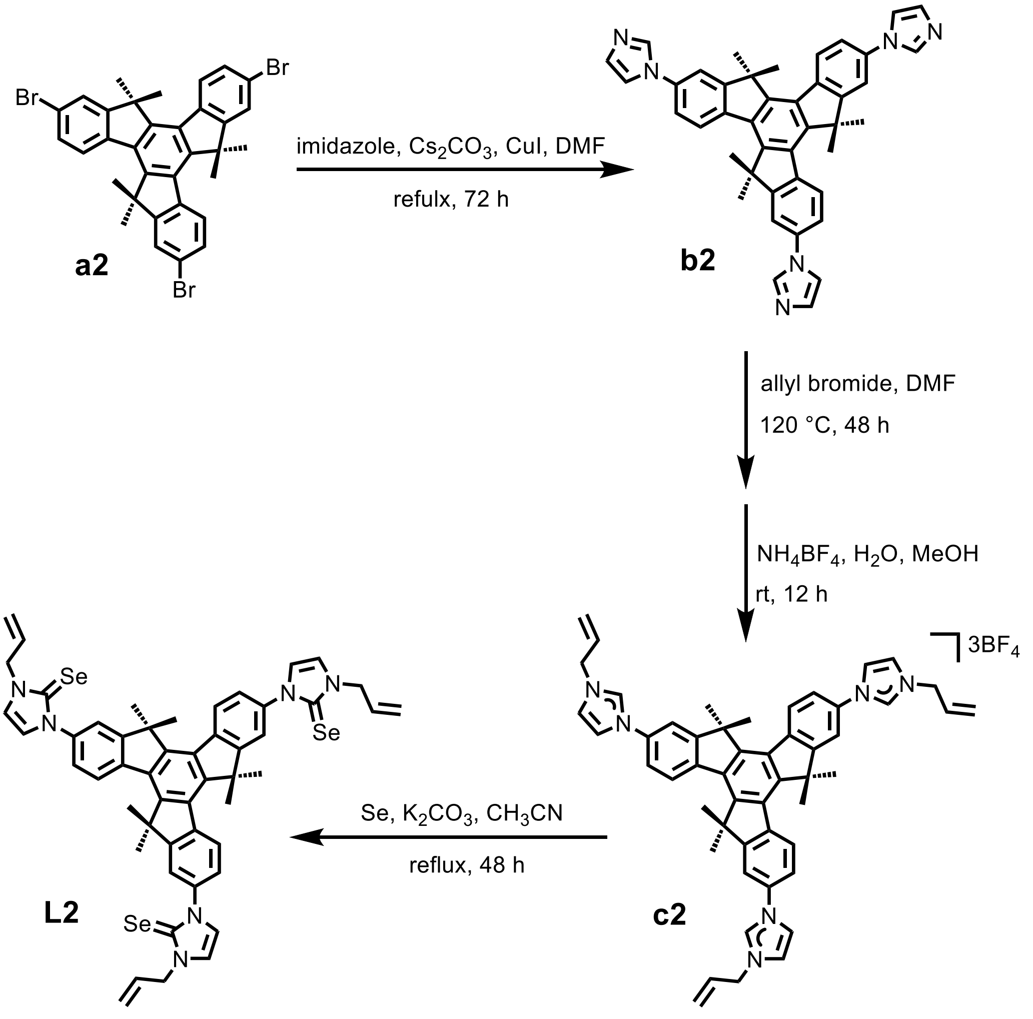

Figure S4. Synthesis of ligand **L2**.

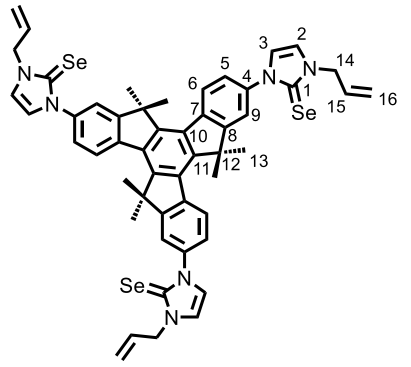

Synthetic route for ligand **L2**: The compound **a2** (664.3 mg, 1.0 mmol), imidazole (1021.2 mg, 15.0 mmol), Cs_2CO_3 (4887.6 mg, 15.0 mmol) and CuI (572.6 mg, 3.0 mmol) were dissolved in anhydrous N,N-dimethylformamide (DMF, 40 mL) under a nitrogen atmosphere. The obtained mixture was refluxed for 72 h under exclusion of light. After cooling to room temperature, the solution was concentrated to 3 mL and H_2O (400 mL) was added to give a C_3-symmetrical core **b2** as a grayish green powder. The grayish green powder **b2** was collected by filtration, washed with H_2O (200 mL), and dried in

vacuo. Yield: 505.4 mg (0.81 mmol, 81%).

Subsequently, the obtained C_3-symmetrical core **b2** (505.4 mg, 0.81 mmol) and allyl bromide (598.2 mg, 4.94 mmol) were dissolved in anhydrous N,N-dimethylformamide (DMF, 10 mL). The resulting mixture was heated at 120 ℃ for 48 h. After cooling to room temperature, the clear solution was concentrated to 2 mL and ethyl acetate (20 mL) was added, leading to the precipitation of a brown solid. The solid was collected by filtration, washed with ethyl acetate (20 mL), and dried in *vacuo*. The obtained solid was dissolved in methanol (3 mL). Upon the addition of a solution of NH_4BF_4 (853.6 mg, 8.14 mmol) in H_2O (40 mL), a pale yellow precipitate formed immediately. The mixture was further stirred at room temperature for 12 h. The precipitate was collected by filtration, washed with H_2O, and dried in *vacuo* to afford trisimidazolium salt **c2**. Yield: 646.5 mg (0.64 mmol, 79%).

The obtained trisimidazolium salt **c2** (646.5 mg, 0.64 mmol), Se (303.6 mg, 3.84 mmol) and K_2CO_3 (885.5 mg, 6.40 mmol) was dissolved in acetonitrile (30 mL). The resulting mixture was refluxed for 48 h. After cooling to room temperature, the solution was evaporated to dryness and extracted with CH_2Cl_2. The filtrate was washed with distilled water (3 × 50 mL). Afterwards, the solution was dried over $MgSO_4$. The clear solution was concentrated to 3 mL and diethyl ether (40 mL) was added to give a yellow solid. The yellow solid **L2** was collected by filtration, washed with diethyl ether, and dried in *vacuo*. Yield: 402.6 mg (0.41 mmol, 82%). ^{1}H NMR (600 MHz, $CDCl_3$): δ = 8.36 (d, J = 8.5 Hz, 3H, H6), 7.87 (d, J = 1.7 Hz, 3H, H9), 7.62 (dd, J = 8.5, 1.7 Hz, 3H, H5), 7.16 (d, J = 2.1 Hz, 3H, H3), 7.05 (d, J = 2.1 Hz, 3H, H2), 6.08–6.02 (m, 3H, H15), 5.40–5.37 (m, 6H, H16), 4.92 (d, J = 6.1 Hz, 6H, H14), 1.92 ppm (s, 18H, H13). ^{13}C{^{1}H} NMR (150 MHz, $CDCl_3$): δ = 157.6 (C8), 156.4 (C1), 149.0 (C11), 137.0 (10), 136.3 (C7), 134.5 (C4), 131.2 (C15), 125.4 (C6), 123.7 (C5), 120.5 (C9), 120.0 (C3), 119.5 (C16), 118.6 (C2), 52.1 (C14), 46.8 (C12), 23.3 (C13) ppm. ESI-MS (positive ions): m/z = 1005.1267 (calcd for [**L2** + Na]$^+$ 1005.1453).

3. Synthesis of cages 1 and 2

3a. Synthesis of cage 1.

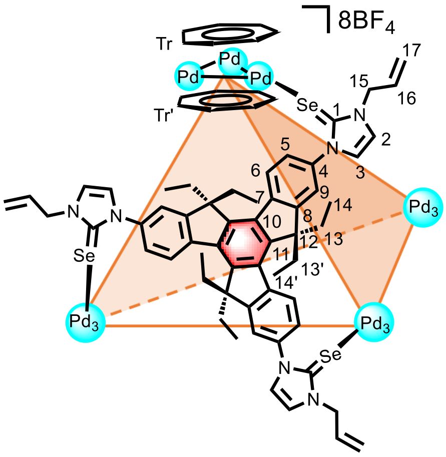

Synthetic route for cage **1**: Ligand **L1** (60.0 mg, 0.056 mmol) was dissolved in CH_2Cl_2 (10 mL), and the solution (acetonitrile, 10 mL) of complex **A** (45.2 mg, 0.056 mmol) was added to the former. The reaction mixture immediately turned to dark red and was stirred at ambient temperature for 24 h. As the reaction finished, the solution was concentrated to 2 mL and diethyl ether (20 mL) was added to obtain a dark red solid **1** which was collected by filtration, washed with diethyl ether, and dried in *vacuo*. Yield: 93.6 mg (0.013 mmol, 96%). ^{1}H NMR (600 MHz, CD_3CN): δ = 7.92 (d, J = 8.5 Hz, 12H, H6), 7.58 (d, J = 2.0 Hz, 12H, H2), 7.45 (d, J = 1.3 Hz, 12H, H9), 7.43 (d, J = 2.0 Hz, 12H, H3), 6.61 (d, J = 8.5 Hz, 12H, H5), 6.22–6.16 (m, 12H, H16), 5.54–5.48 (m, 24H, H17), 5.31 (dd, J = 15.5, 5.3 Hz, 12H, H15), 5.12 (dd, J = 15.5, 5.3 Hz, 12H, H15), 4.02 (s, 28H, H_{Tr}), 3.36 (s, 28H, $H_{Tr'}$), 2.74–2.67 (m, 12H, H13'), 2.65−2.62 (m, 12H, H13), 2.16 (br, 12H, H13), 2.12–2.08 (m, 12H, H13'), 0.39 (br, 36H, H14), 0.10 ppm (t, J = 6.9 Hz, 36H, H14'). ^{13}C{^{1}H} NMR (150 MHz, CD_3CN): δ = 154.4 (C8), 147.5 (C1), 146.7 (C11), 141.5 (C7), 138.9 (C10), 137.8 (C4), 132.6 (C16), 126.3 (C5), 125.6 (C3), 125.1 (C6), 124.4 (C9), 123.3 (C2), 120.7 (C17), 73.1 (C_{Tr}), 71.4 ($C_{Tr'}$), 57.9 (C12), 53.8 (C15), 30.0 (C13'), 29.4 (C13), 10.5 (C14), 8.5 (C14') ppm. ^{19}F NMR (565 MHz, CD_3CN): δ = –151.59 and –151.65 ppm. ESI-MS (positive ions): m/z = 908.1794 (calcd for [**1** – $7BF_4]^{7+}$ 908.1797); 1073.8852 (calcd for [**1** – $6BF_4]^{6+}$ 1073.8770); 1306.0557 (calcd for [**1** – $5BF_4]^{5+}$ 1306.0532); 1654.3189 (calcd for [**1** – $4BF_4]^{4+}$ 1654.3176); 2234.7636 (calcd for [**1** – $3BF_4]^{3+}$ 2234.7581); 1664.5666 (calcd for {[**1** – $4BF_4$] + $CH_3CN\}^{4+}$ 1664.5742); 2248.4348 (calcd for {[**1** – $3BF_4$] + $CH_3CN\}^{3+}$ 2248.4337).

3b. Synthesis of cage 2.

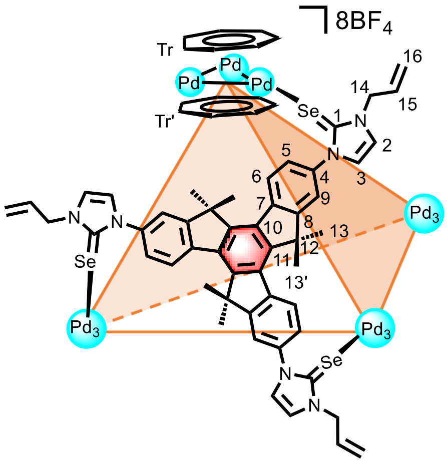

Synthetic route for cage **2**: Ligand **L2** (55.2 mg, 0.056 mmol) was dissolved in CH_2Cl_2 (10 mL), and the solution (acetonitrile, 10 mL) of complex **A** (45.3 mg, 0.056 mmol) was added to the former. The reaction mixture immediately turned to dark red and was stirred at ambient temperature for 24 h. As the reaction finished, the solution was concentrated to 2 mL and diethyl ether (20 mL) was added to obtain a dark red solid **2** which was collected by filtration, washed with diethyl ether, and dried in *vacuo*. Yield: 90.5 mg (0.013 mmol, 96%). ^{1}H NMR (600 MHz, CD_3CN): δ = 7.82 (d, *J* = 8.5 Hz, 12H, H6), 7.65 (s, 12H, H9), 7.60 (d, *J* = 2.2 Hz, 12H, H2), 7.43 (d, *J* = 2.2 Hz, 12H, H3), 6.58 (d, *J* = 8.5 Hz, 12H, H5), 6.24–6.18 (m, 12H, H15), 5.55–5.51 (m, 24H, H16), 5.33 (dd, *J* = 15.4, 5.5 Hz, 12H, H14), 5.14 (dd, *J* = 15.5, 5.3 Hz, 12H, H14), 3.94 (s, 28H, H_{Tr}), 3.28 (s, 28H, $H_{Tr'}$), 1.73 (s, 36H, H13), 1.67 ppm (s, 36H, H13'). ^{13}C{^{1}H} NMR (150 MHz, CD_3CN): δ = 158.5 (C8), 150.9 (C11), 148.2 (C1), 137.7 (C4), 137.2 (C7), 135.7 (C10), 132.6 (C15), 126.5 (C5), 126.1 (C6), 125.3 (C3), 124.8 (C9), 123.4 (C2), 120.9 (C16), 73.4 (C_{Tr}), 71.9 ($C_{Tr'}$), 53.8 (C14), 47.9 (C12), 24.7 (C13), 24.2 (C13') ppm. ^{19}F NMR (565 MHz, CD_3CN): δ = –151.60 and –151.65 ppm. ESI-MS (positive ions): *m/z* = 1017.7811 (calcd for {[**2** – $6BF_4]^{6+}$ 1017.8142); 1238.7227 (calcd for [**2** – $5BF_4]^{5+}$ 1238.7779); 1570.1821 (calcd for [**2** – $4BF_4]^{4+}$ 1570.2234).

4. NMR spectra and ESI-MS spectra

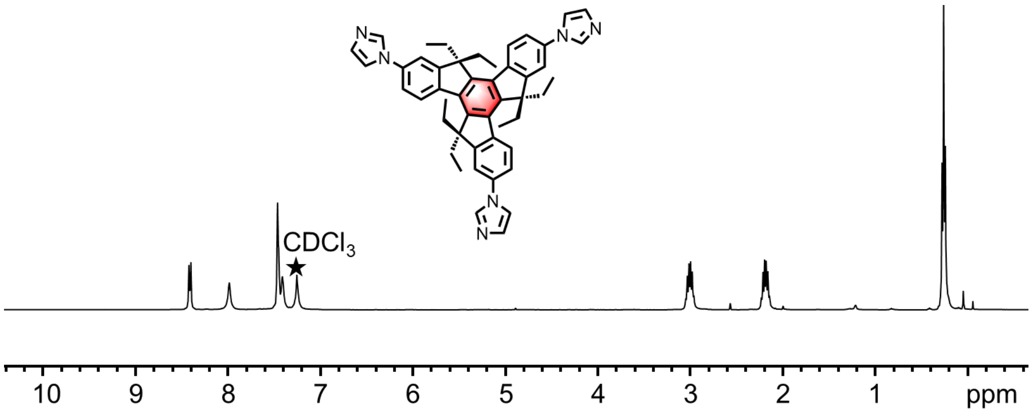

Figure S5. ^{1}H NMR spectrum (400 MHz, $CDCl_3$, 298 K) of compound **b1**.

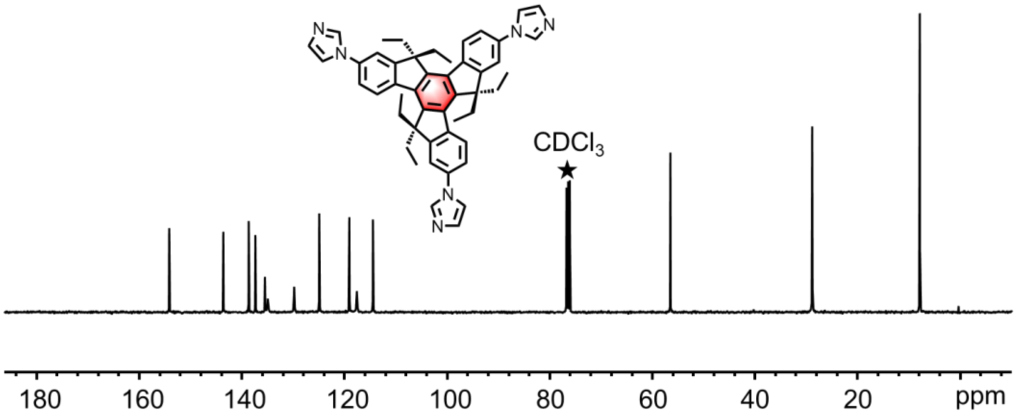

Figure S6. $^{13}C\{^{1}H\}$ NMR spectrum (100 MHz, $CDCl_3$, 298 K) of compound **b1**.

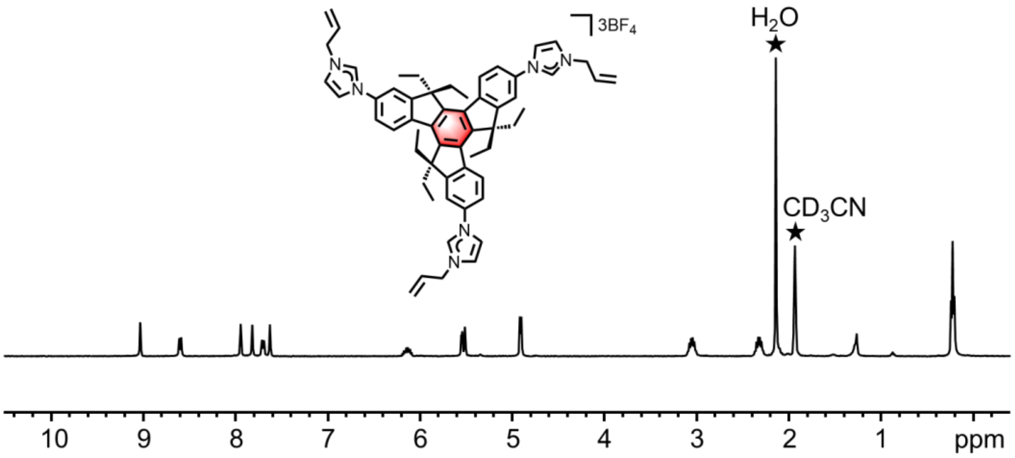

Figure S7. ^{1}H NMR spectrum (400 MHz, CD_3CN, 298 K) of **c1**.

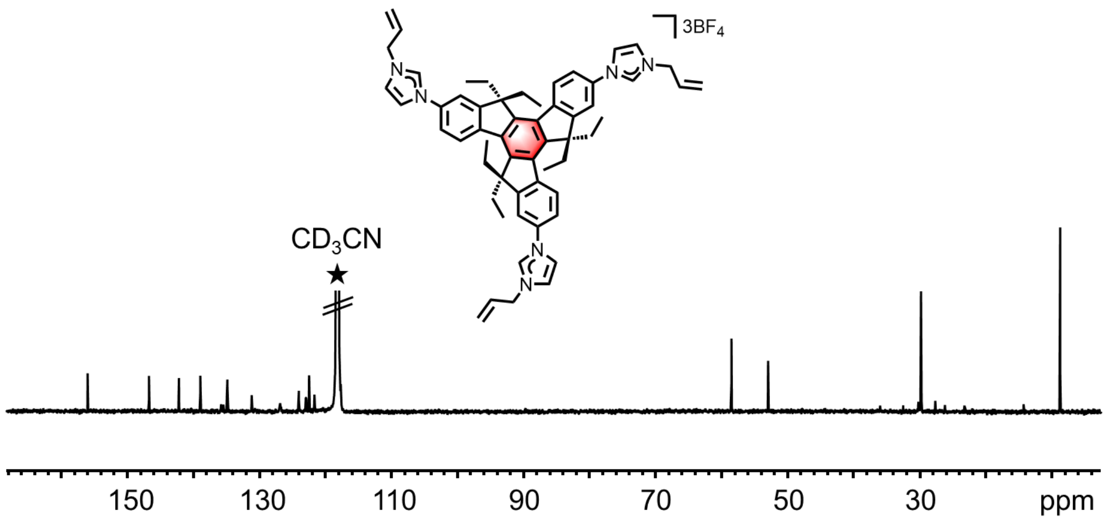

Figure S8. $^{13}C\{^1H\}$ NMR spectrum (100 MHz, CD_3CN, 298 K) of **c1**.

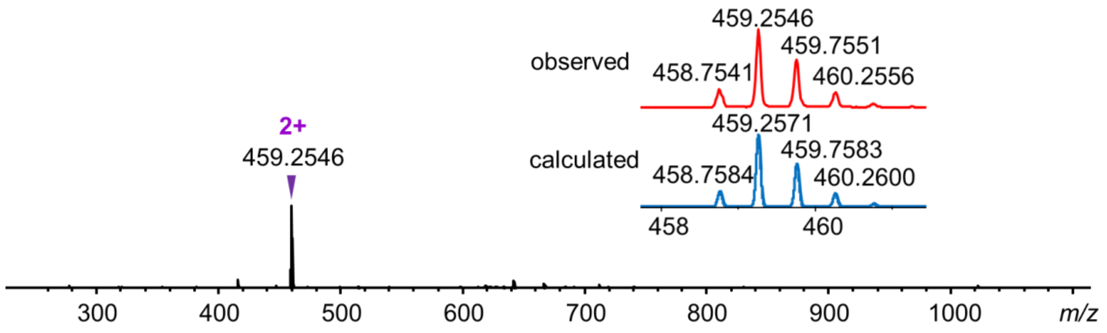

Figure S9. ESI mass spectrum (positive ions) of **c.** Calculated (bule) and experimental (red) isotope distribution of **c1**: [**c1** – $2BF_4]^{2+}$.

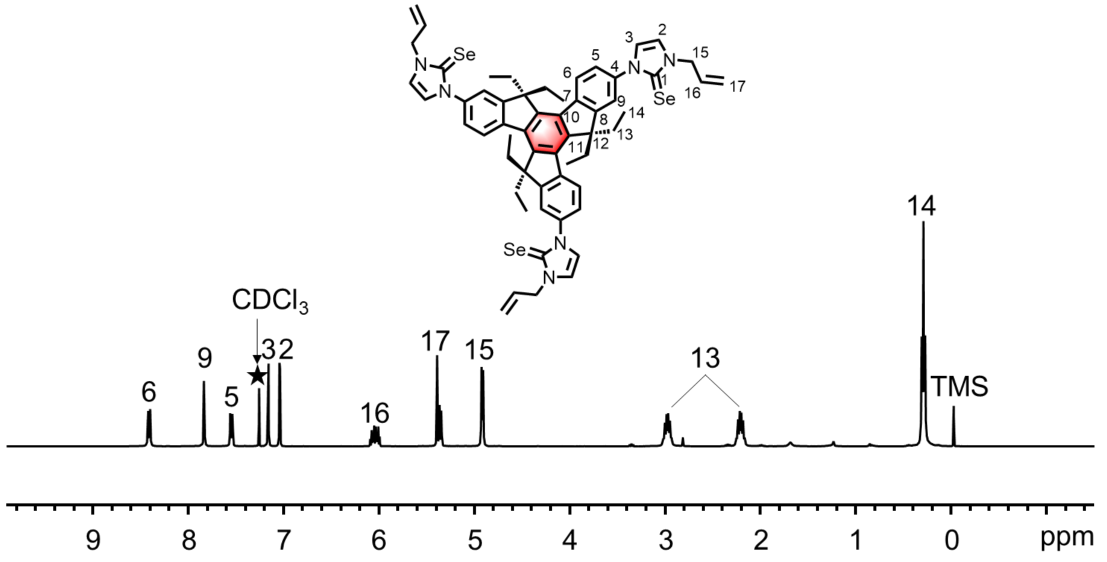

Figure S10. ^1H NMR spectrum (400 MHz, $CDCl_3$, 298 K) of ligand **L1**.

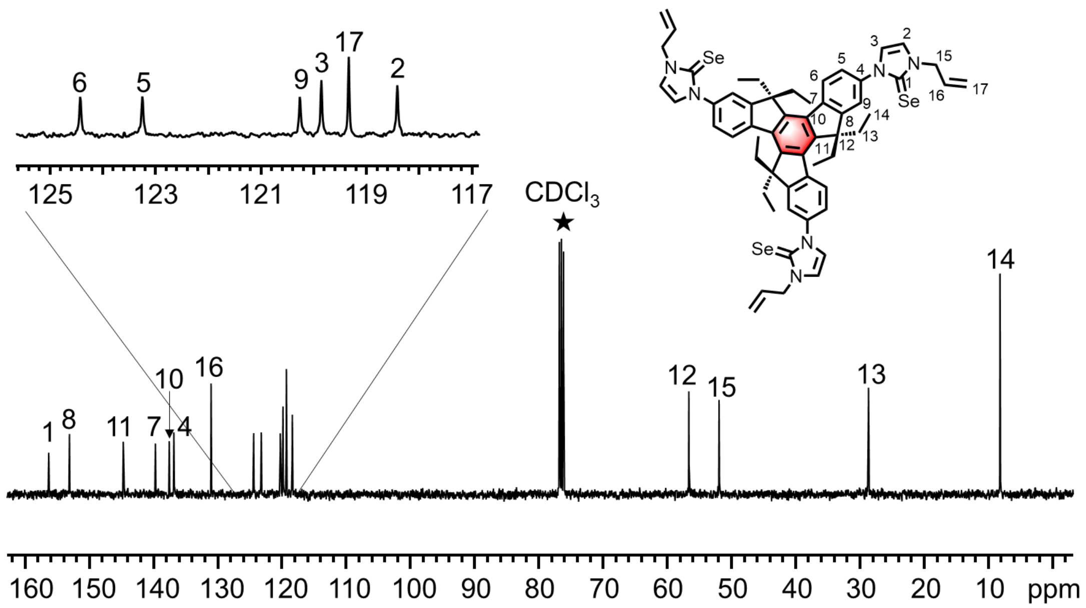

Figure S11. $^{13}C\{^{1}H\}$ NMR spectrum (100 MHz, $CDCl_3$, 298 K) of ligand **L1**.

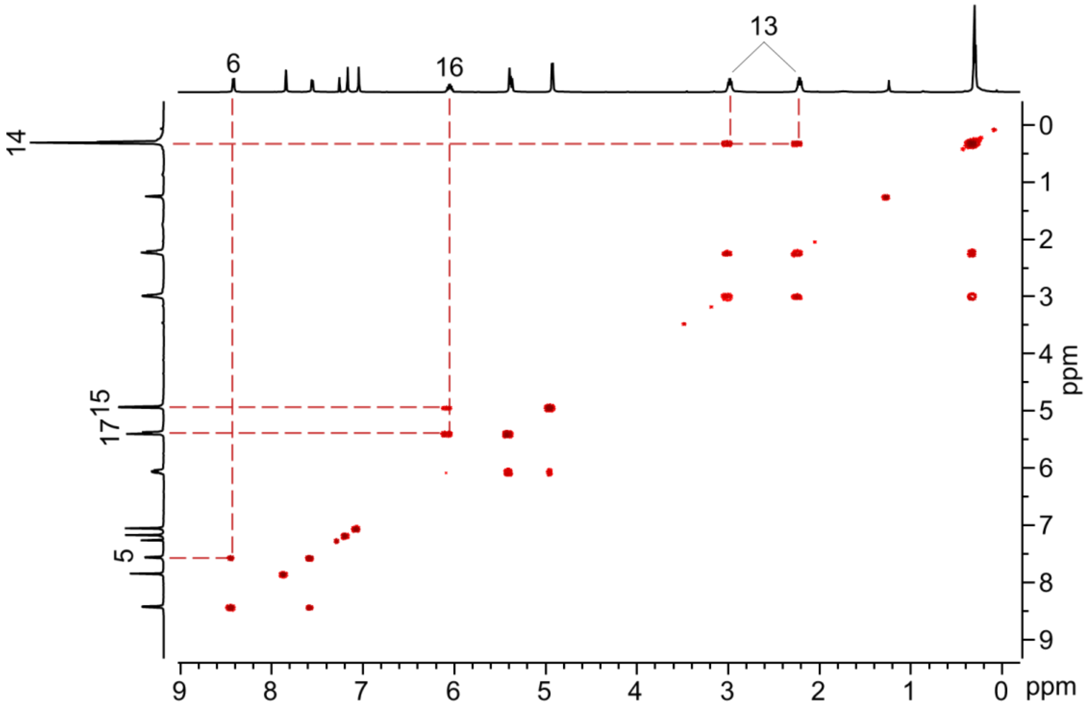

Figure S12. ^{1}H-^{1}H COSY NMR spectrum (400 MHz, $CDCl_3$, 298 K) of ligand **L1**.

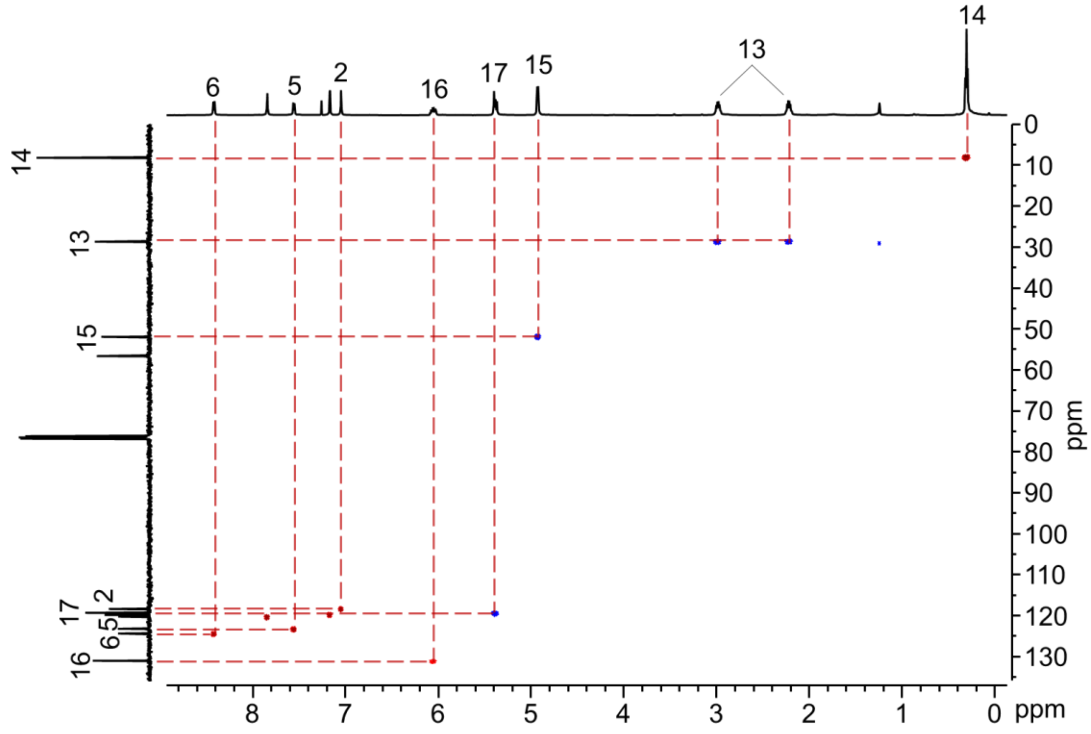

Figure S13. ^{1}H-^{13}C HSQC NMR spectrum (400 MHz, $CDCl_3$, 298 K) of ligand **L1**.

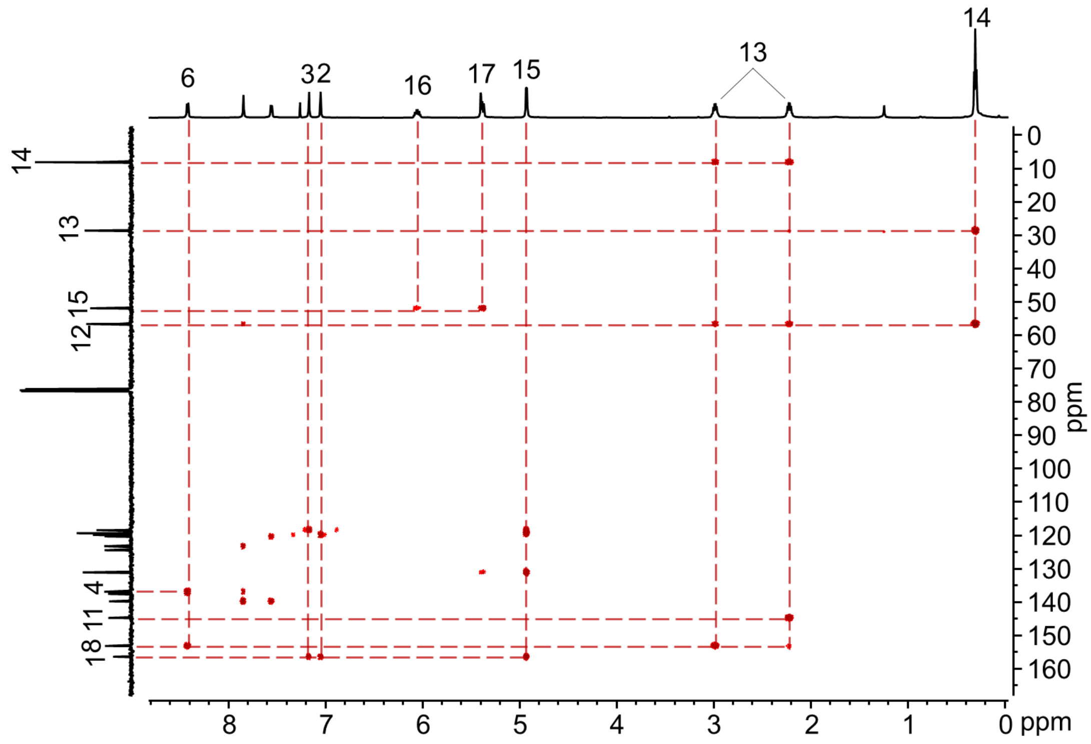

Figure S14. ^{1}H-^{13}C HMBC NMR spectrum (400 MHz, $CDCl_3$, 298 K) of ligand **L1**.

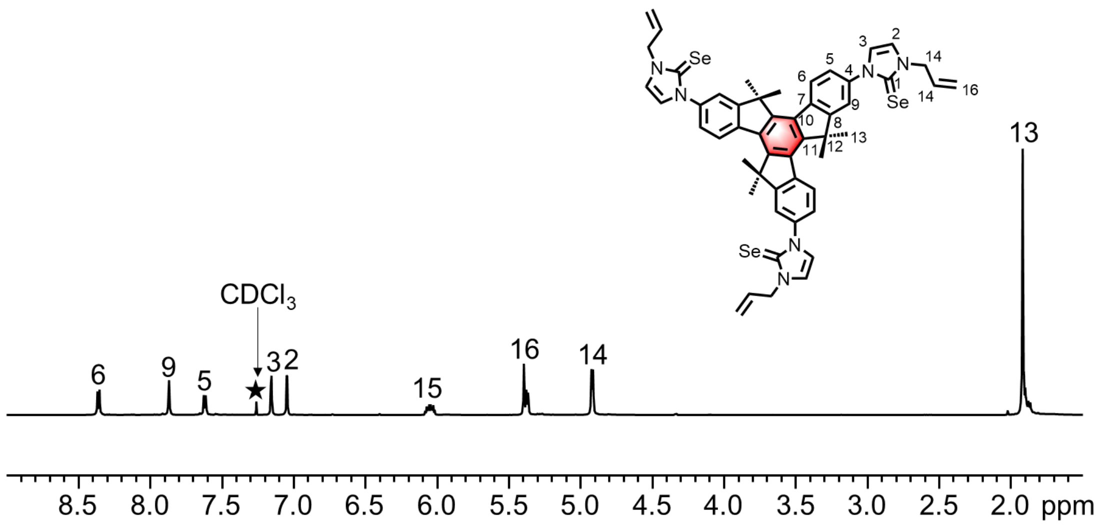

Figure S15. ^{1}H NMR spectrum (600 MHz, CDCl$_3$, 298 K) of ligand **L2**.

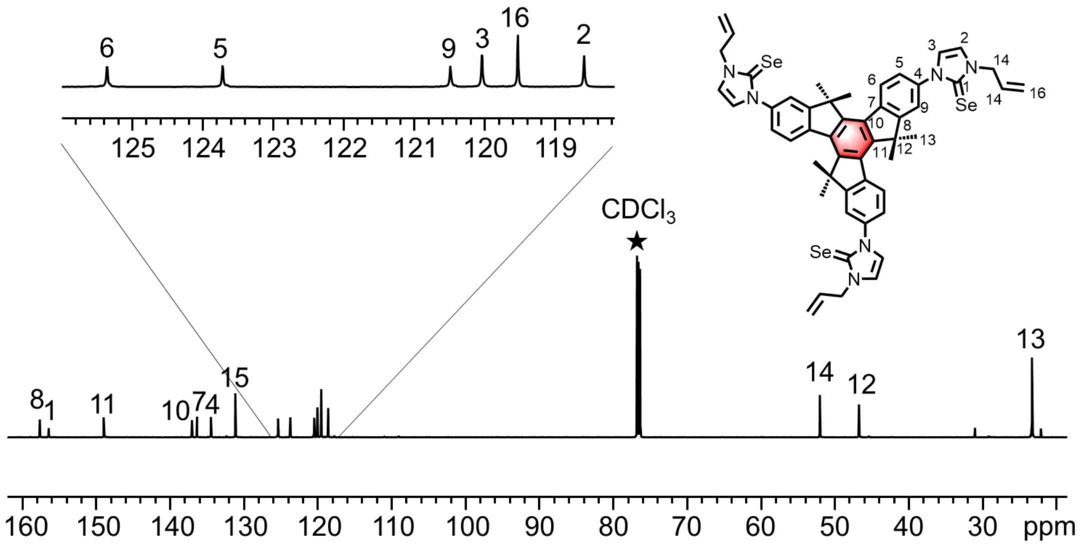

Figure S16. ^{13}C{^{1}H} NMR spectrum (150 MHz, CDCl$_3$, 298 K) of ligand **L2**.

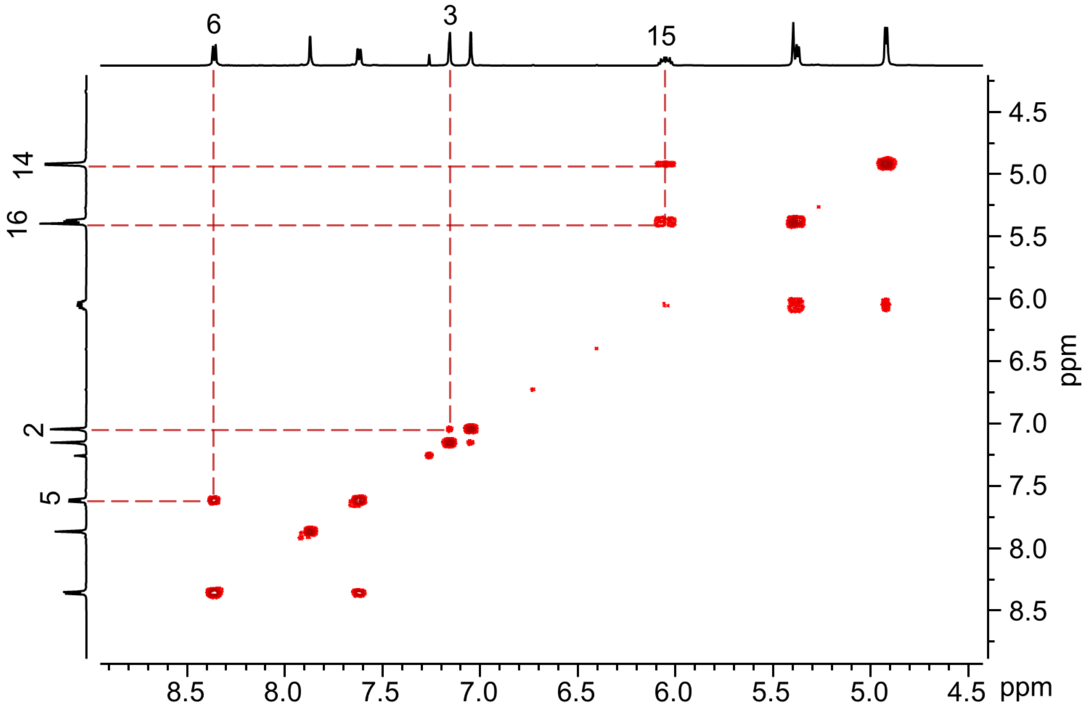

Figure S17. ^{1}H-^{1}H COSY NMR spectrum (600 MHz, $CDCl_3$, 298 K) of ligand **L2**.

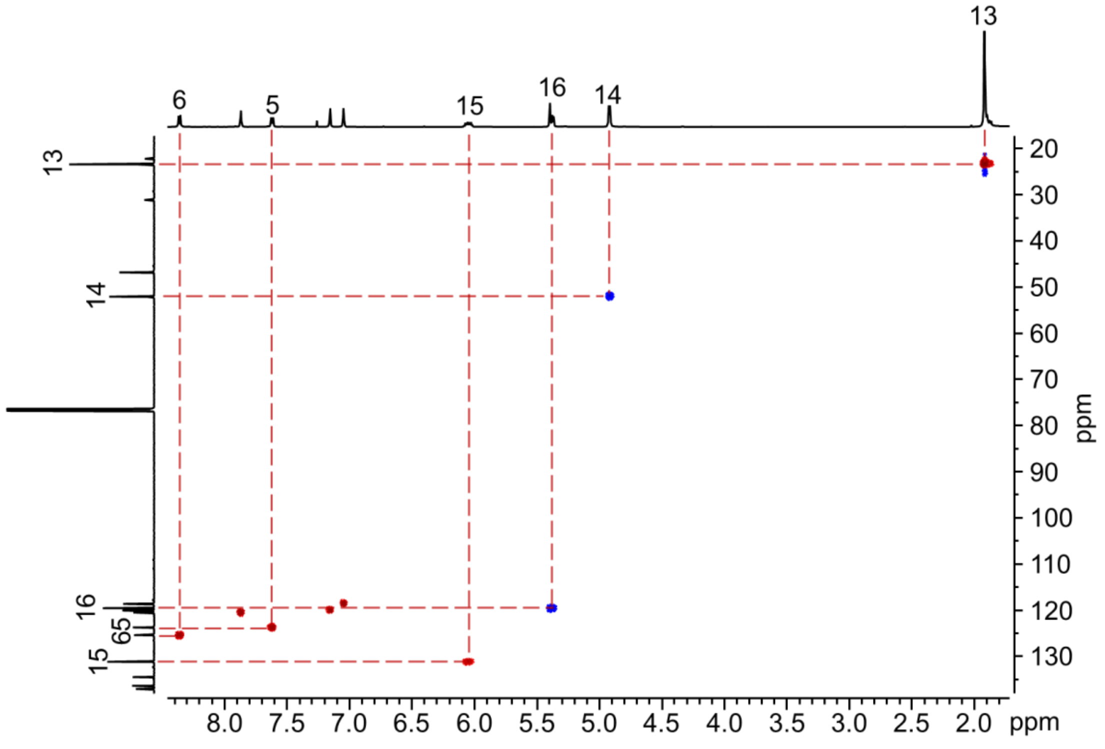

Figure S18. ^{1}H-^{13}C HSQC NMR spectrum (600 MHz, $CDCl_3$, 298 K) of ligand **L2**.

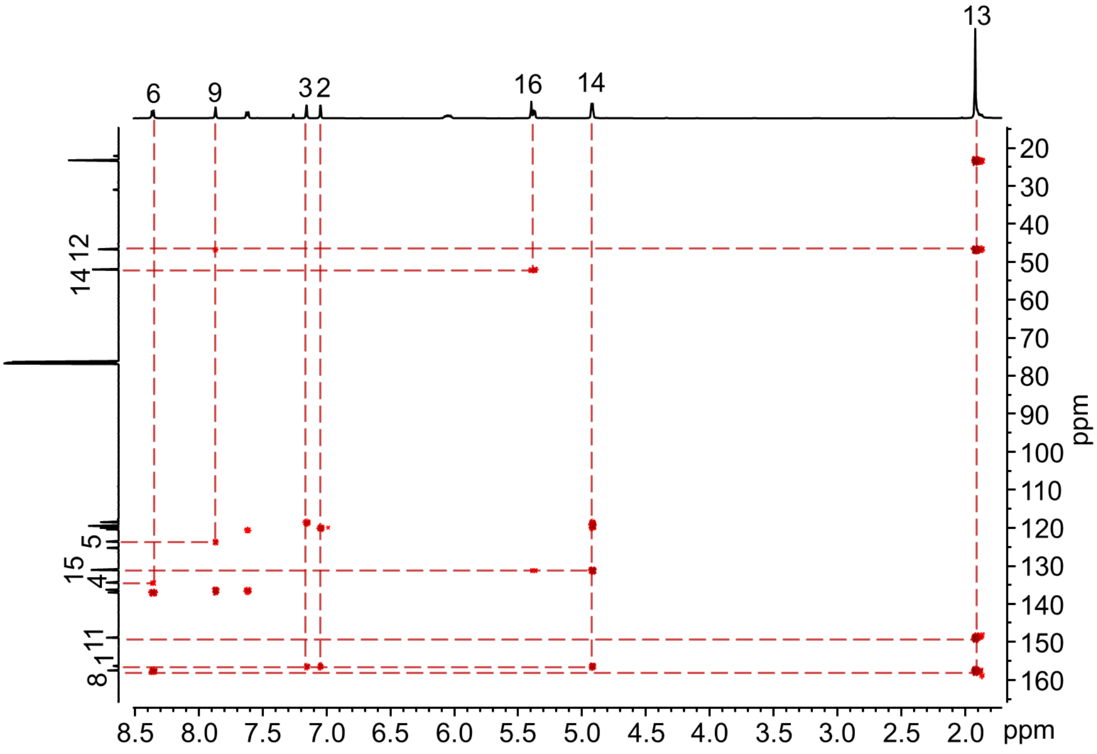

Figure S19. ^1H-^{13}C HMBC NMR spectrum (600 MHz, $CDCl_3$, 298 K) of ligand **L2**.

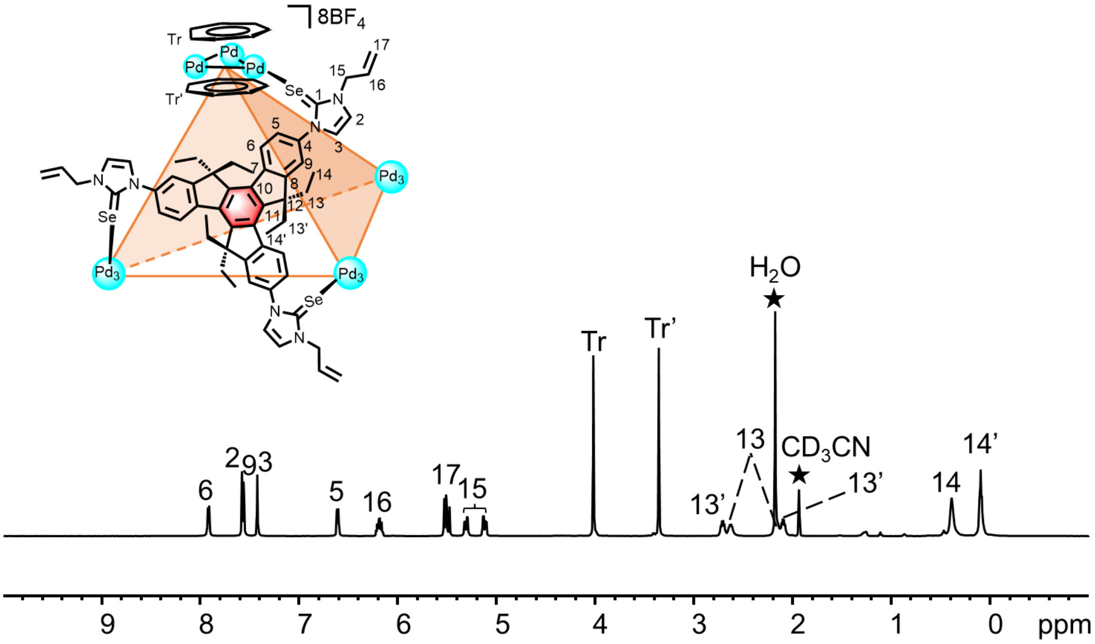

Figure S20. ^1H NMR spectrum (600 MHz, CD_3CN, 298 K) of cage **1**.

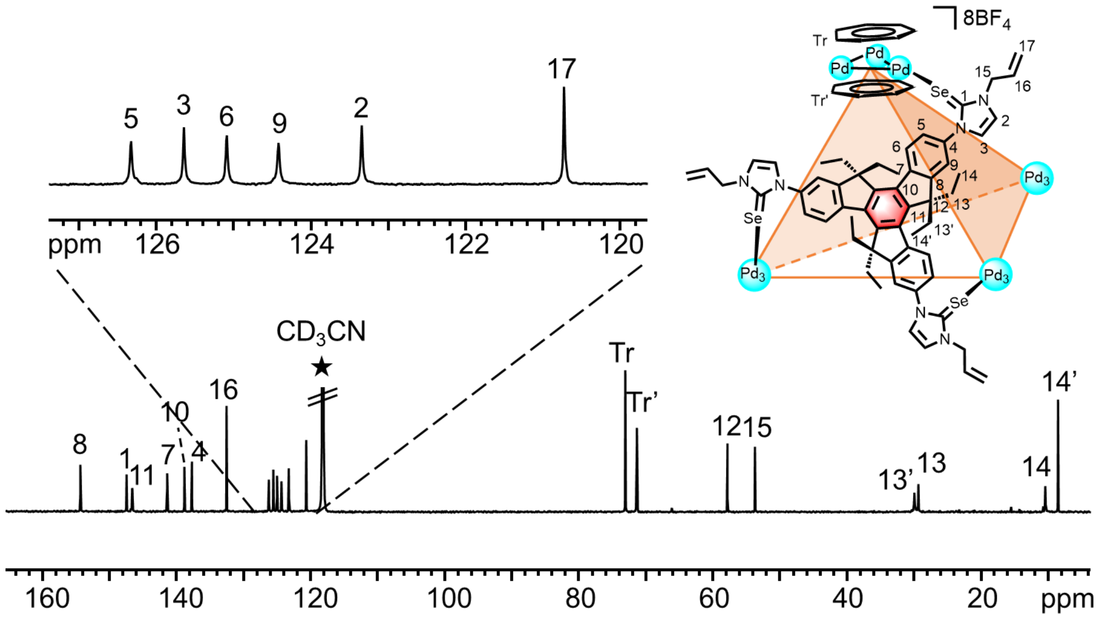

Figure S21. $^{13}C\{^{1}H\}$ NMR spectrum (150 MHz, CD_3CN, 298 K) of cage **1**.

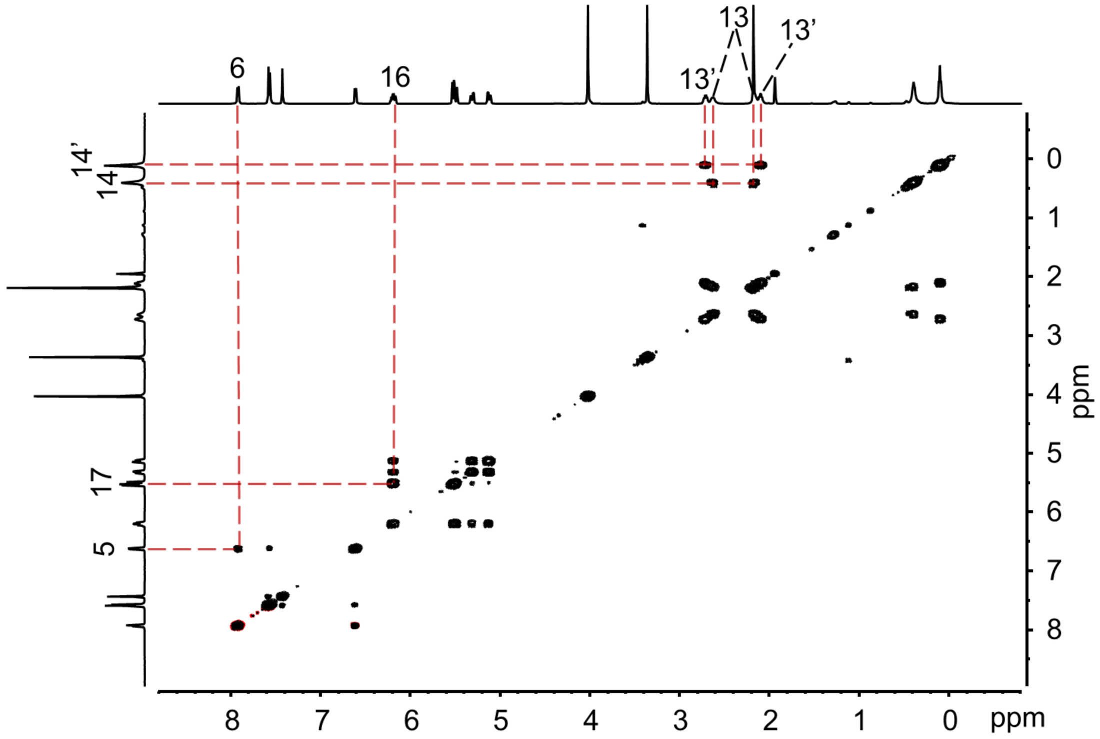

Figure S22. ^{1}H-^{1}H COSY NMR spectrum (600 MHz, CD_3CN, 298 K) of cage **1**.

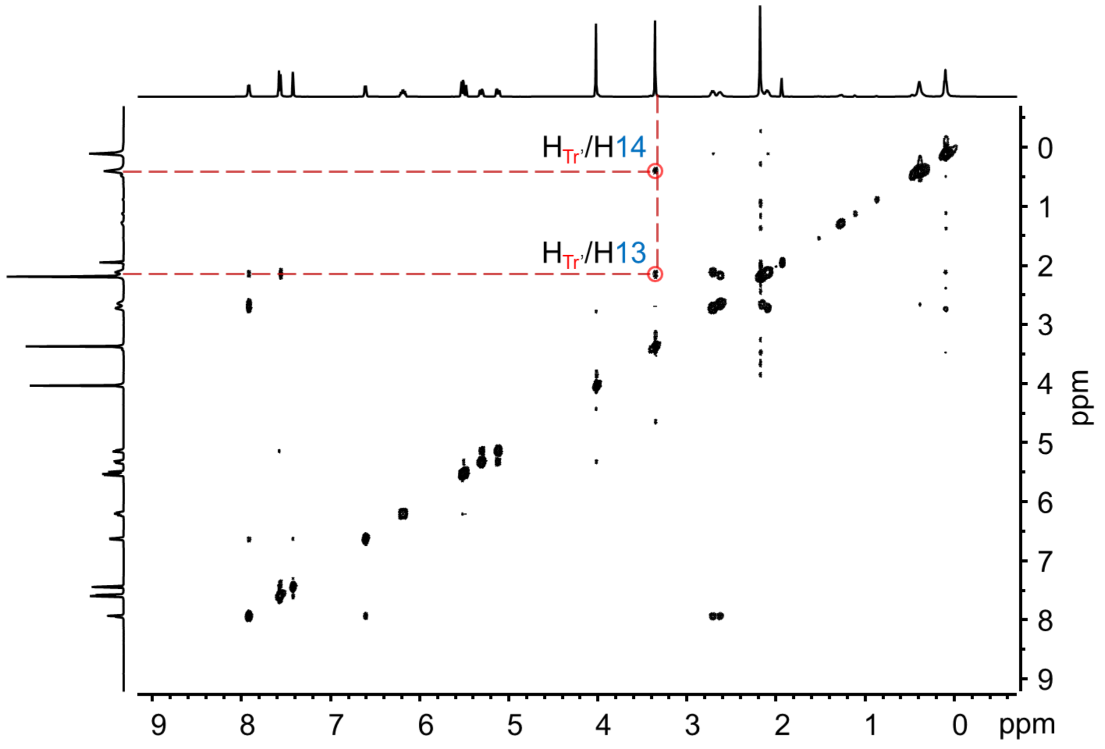

Figure S23. ^{1}H-^{1}H NOESY NMR spectrum (600 MHz, CD_3CN, 298 K) of cage **1**.

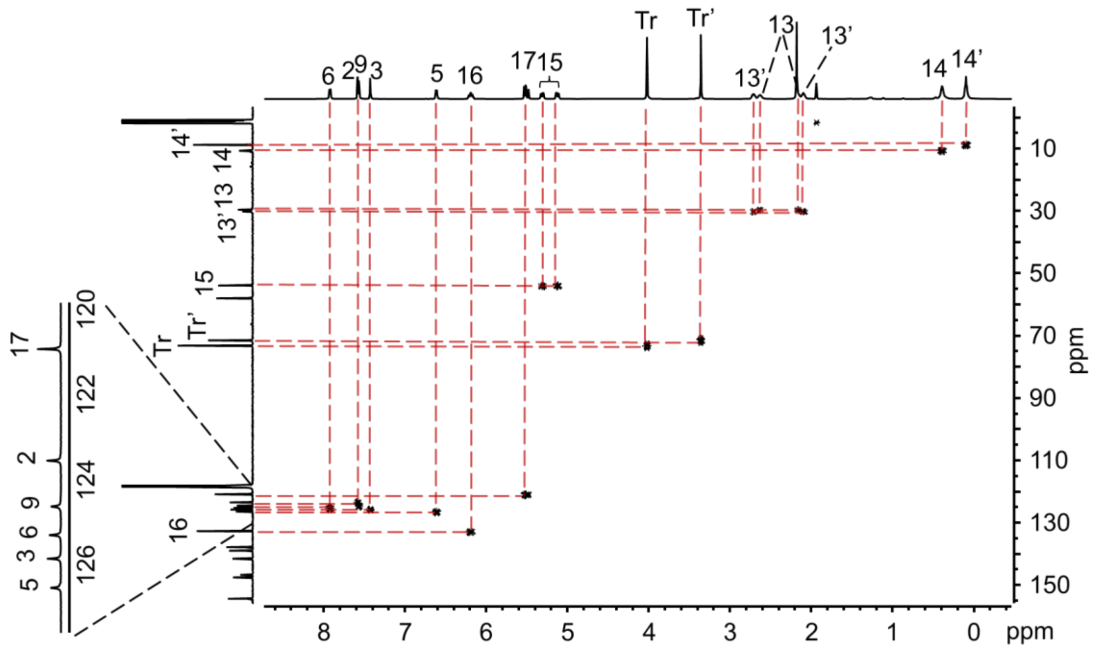

Figure S24. ^{1}H-^{13}C HSQC NMR spectrum (600 MHz, CD_3CN, 298 K) of cage **1**.

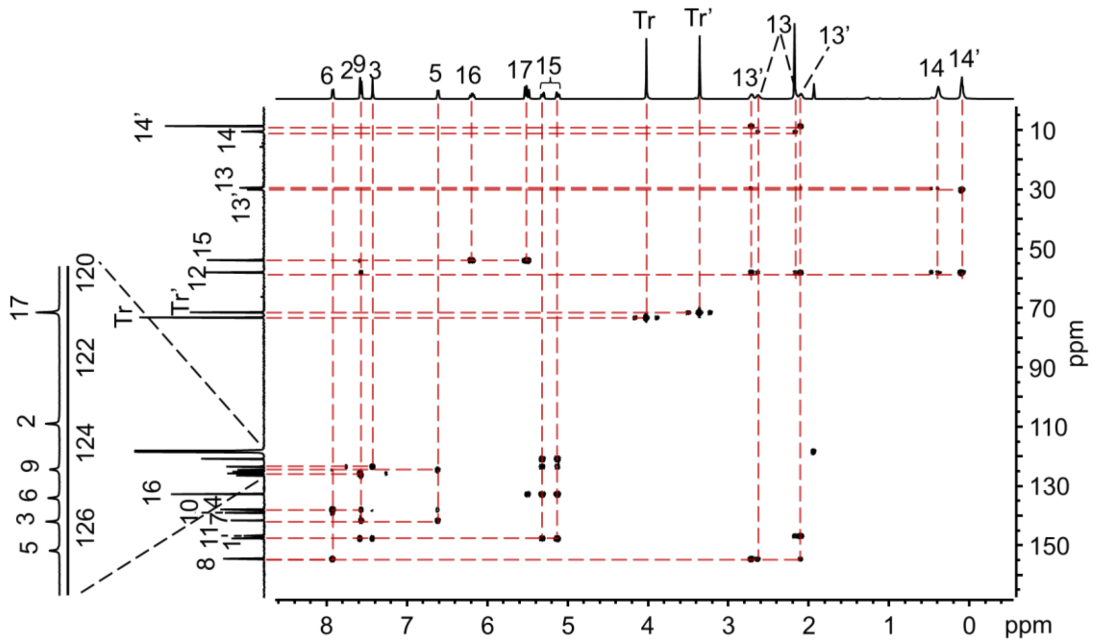

Figure S25. ^{1}H-^{13}C HMBC NMR spectrum (600 MHz, CD_3CN, 298 K) of cage **1**.

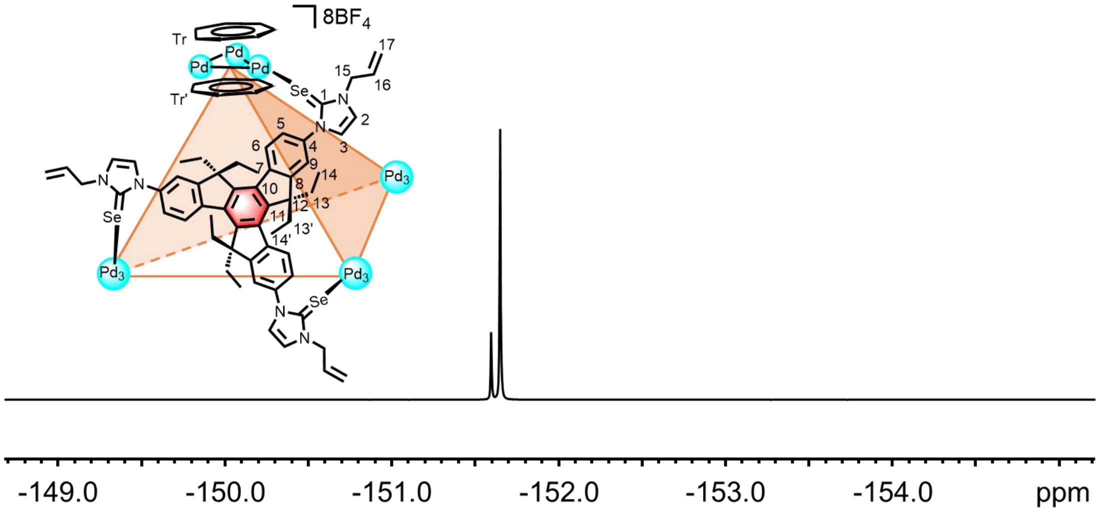

Figure S26. ^{19}F NMR spectrum (565 MHz, CD_3CN, 298 K) of cage **1**.

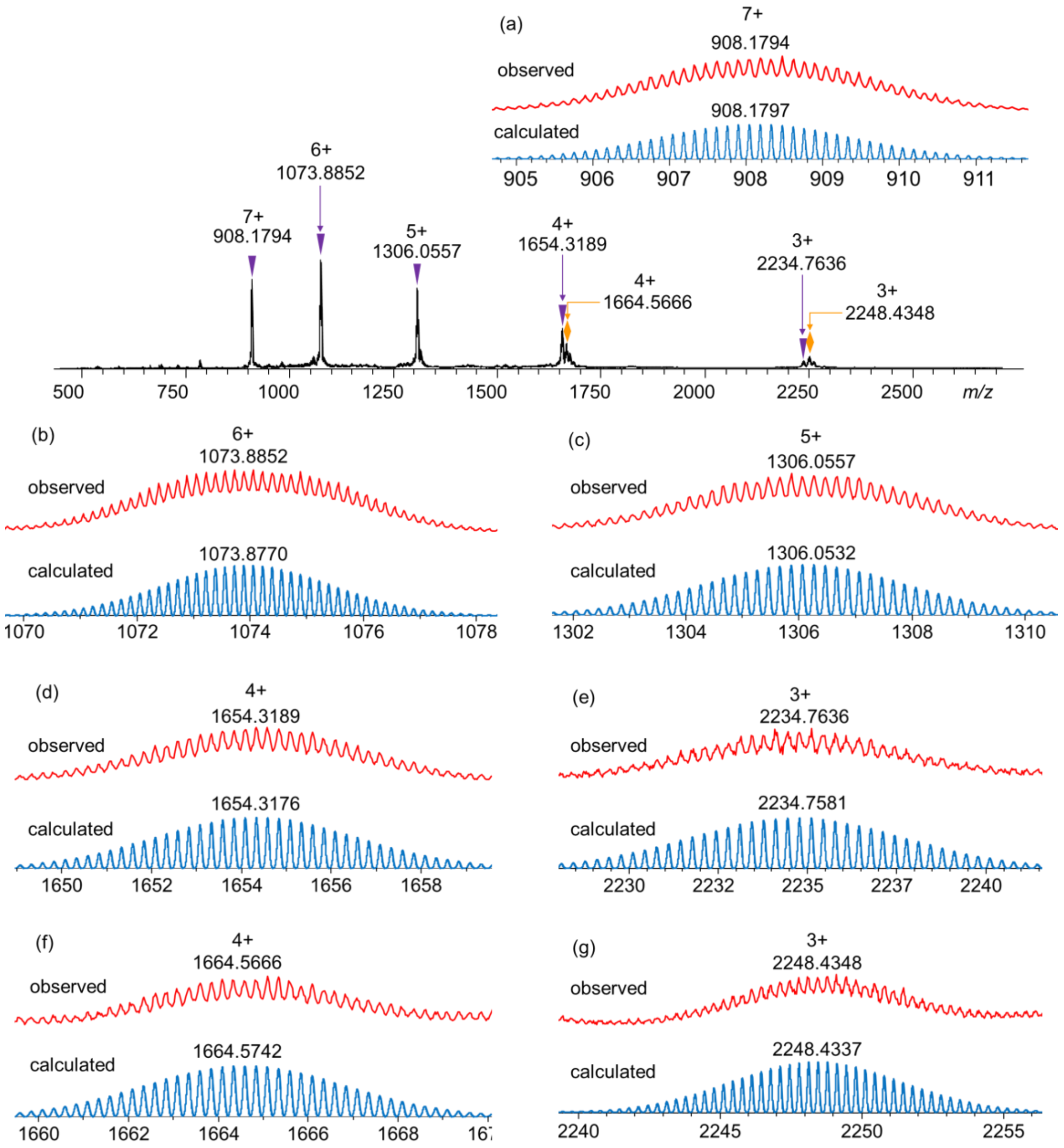

Figure S27. ESI mass spectrum (positive ions) of cage **1.** Calculated (blue) and experimental (red) isotope distribution (positive ions) of cage **1**: (a) [**1** – 7BF_4]$^{7+}$; (b) [**1** – 6BF_4]$^{6+}$; (c) [**1** – 5BF_4]$^{5+}$; (d) [**1** – 4BF_4]$^{4+}$; (e) [**1** – 3BF_4]$^{3+}$; (f) {[**1** – 4BF_4] + CH_3CN}$^{4+}$; (g) {[**1** – 3BF_4] + CH_3CN}$^{3+}$. (▼) Cage **1**. (♦) Cage **1** complexing with acetonitrile.

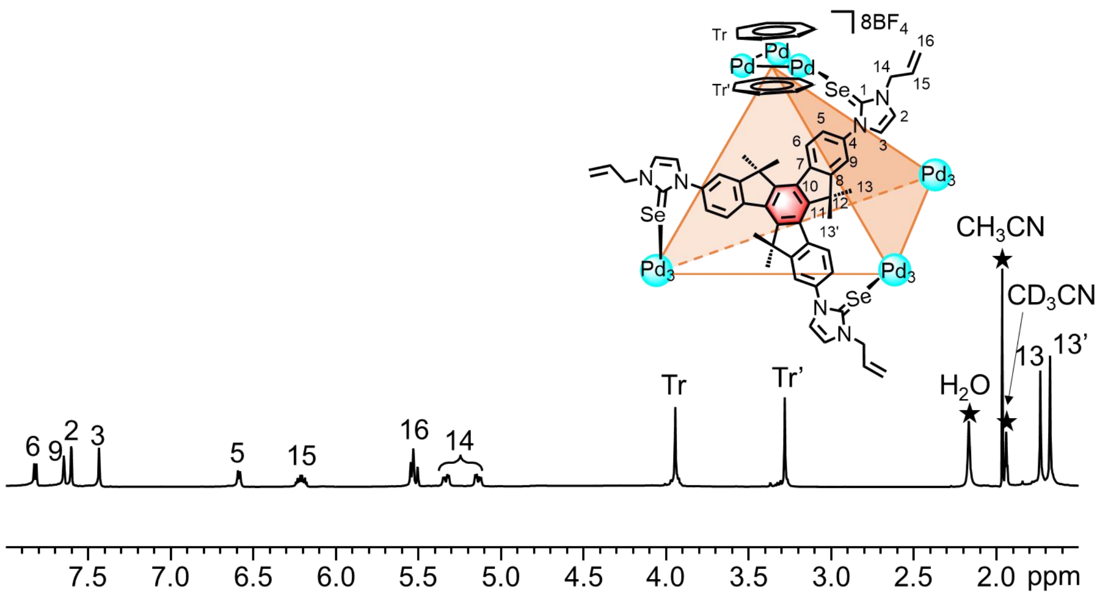

Figure S28. ^{1}H NMR spectrum (600 MHz, CD_3CN, 298 K) of cage **2**.

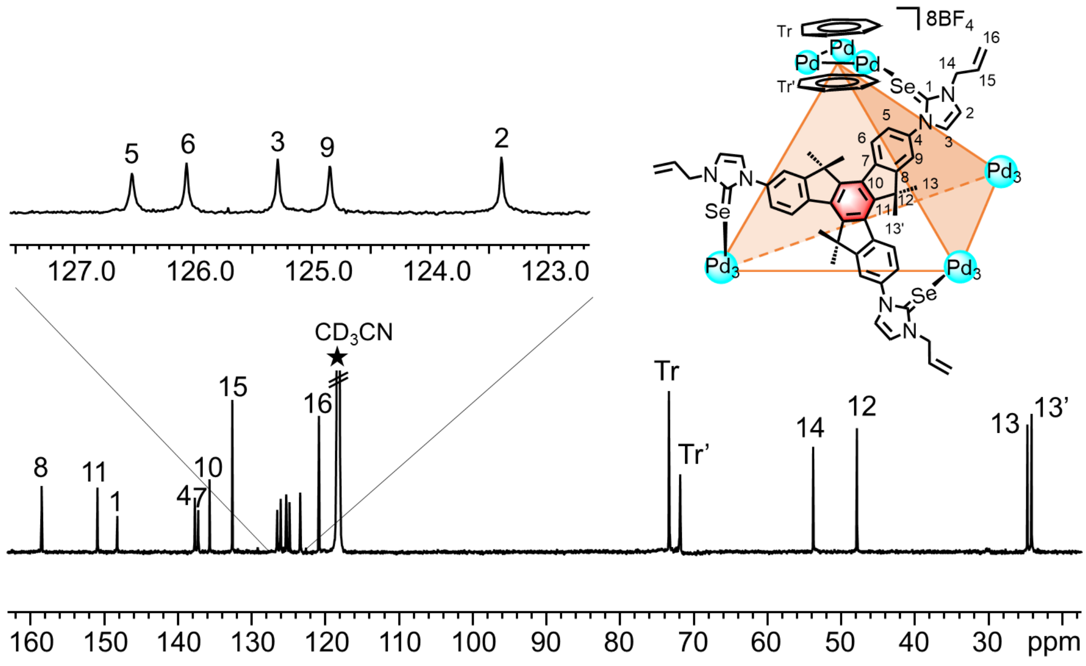

Figure S29. ^{13}C{^{1}H} NMR spectrum (150 MHz, CD_3CN, 298 K) of cage **2**.

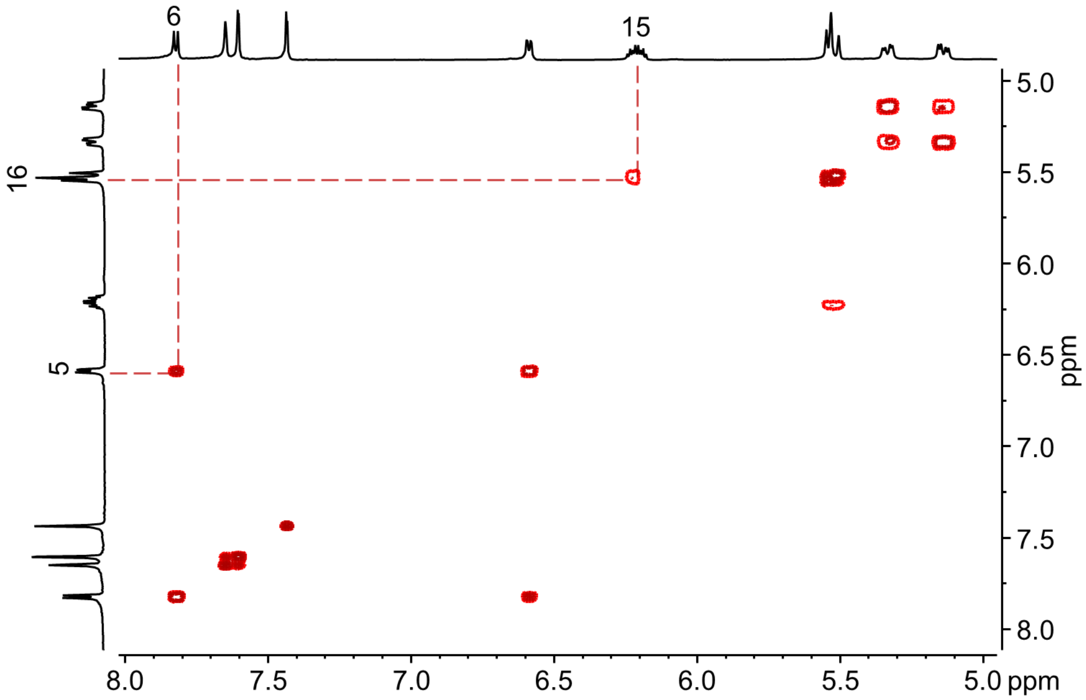

Figure S30. ^{1}H-^{1}H COSY NMR spectrum (600 MHz, CD_3CN, 298 K) of cage **2**.

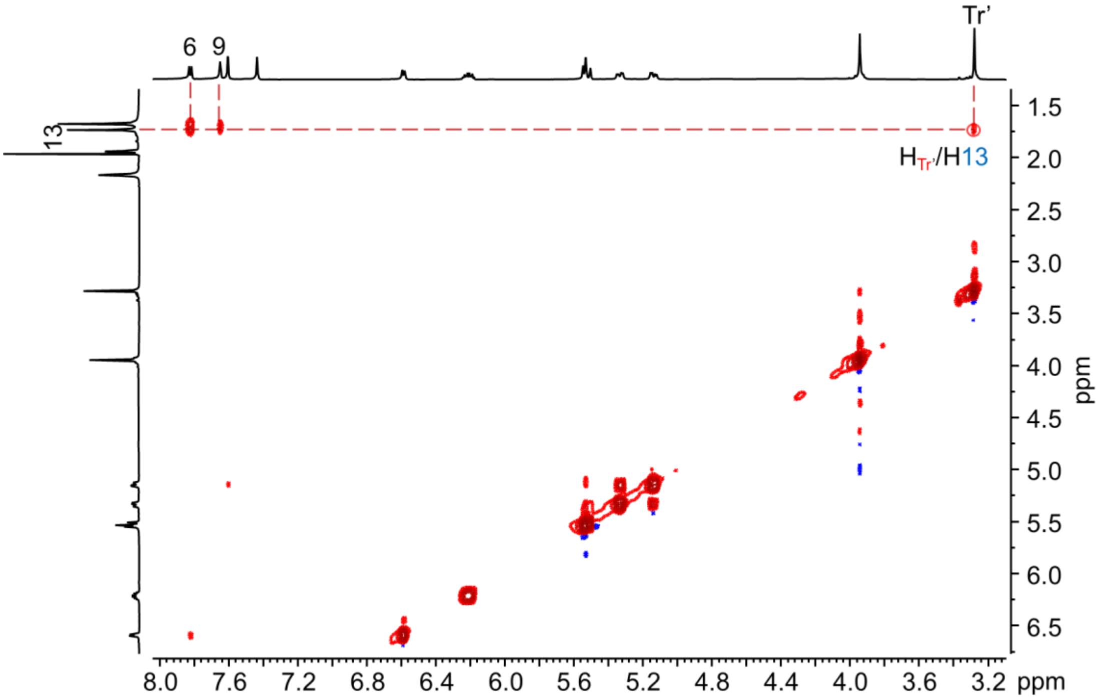

Figure S31. ^{1}H-^{1}H NOESY NMR spectrum (600 MHz, CD_3CN, 298 K) of cage **2**.

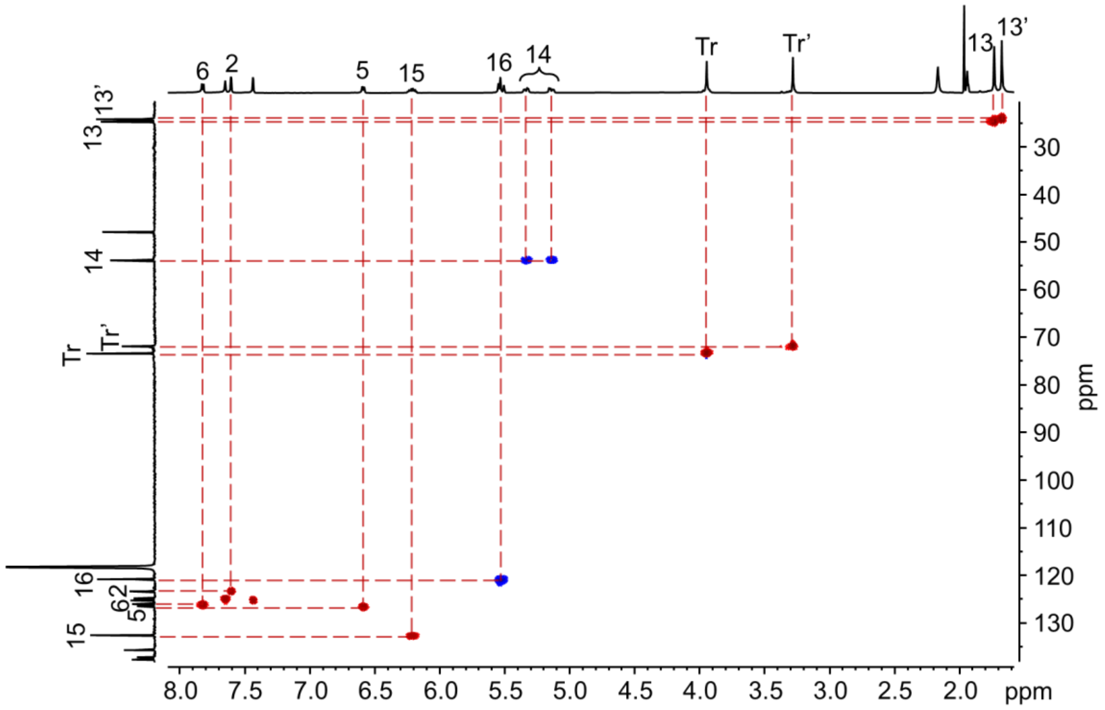

Figure S32. ^{1}H-^{13}C HSQC NMR spectrum (600 MHz, CD_3CN, 298 K) of cage **2**.

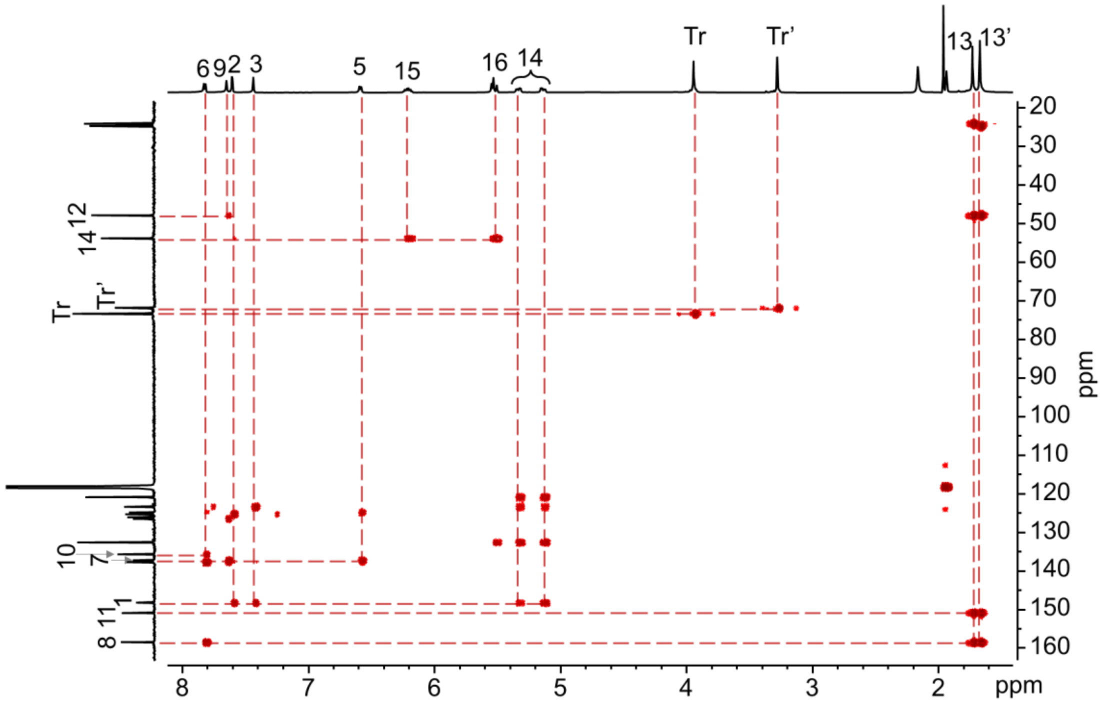

Figure S33. ^{1}H-^{13}C HMBC NMR spectrum (600 MHz, CD_3CN, 298 K) of cage **2**.

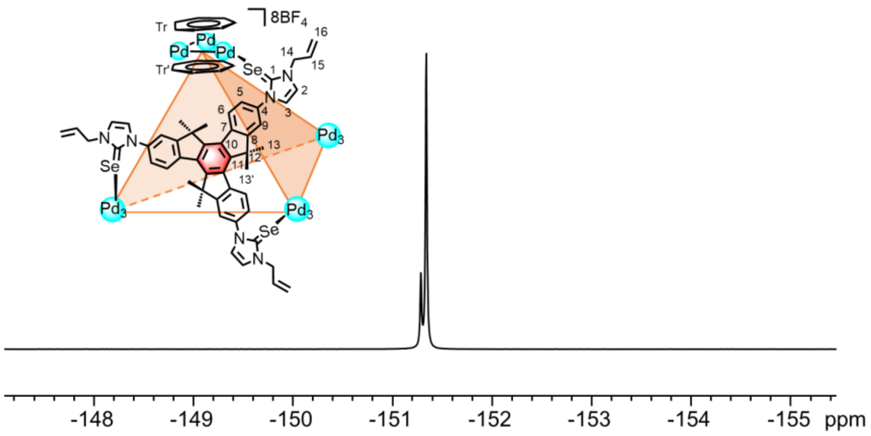

Figure S34. ^{19}F NMR spectrum (565 MHz, CD_3CN, 298 K) of cage **2**.

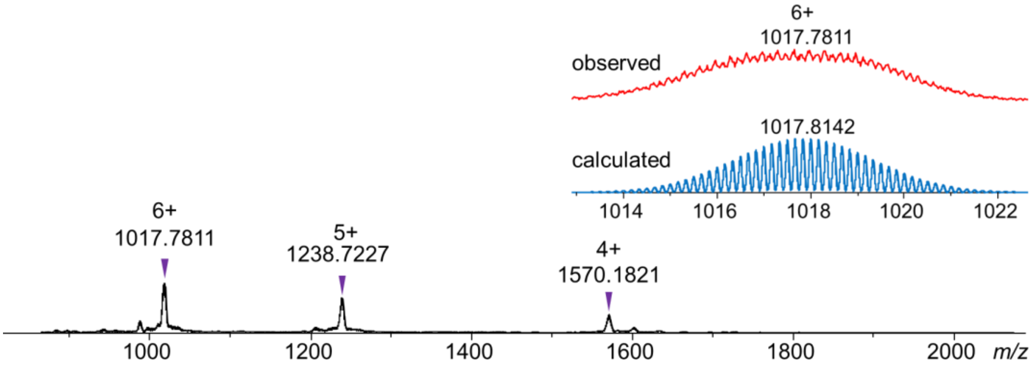

Figure S35. HR-ESI mass spectrum (positive ions) of cage **2**. Calculated (blue) and experimental (red) isotope distribution (positive ions) of cage **2**: $[\mathbf{2} - 6BF_4]^{6+}$.

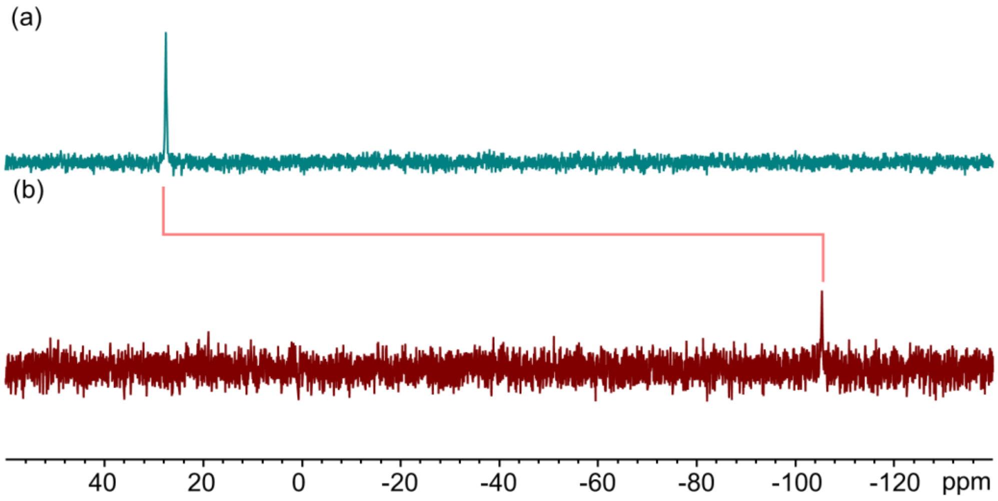

Figure S36. ^{77}Se NMR spectra (CD_3CN, 114 MHz, 298 K) of (a) ligand **L2** and (b) cage **2**.

5. Volume calculations with VOIDOO

In order to determine the available void space within **1**, and **2**, VOIDOO calculations based on the crystal structure were performed [S3]. A virtual probe with a radius of 1.4 Å (set by default, water-sized) was employed, and the following the parameters were involved in the calculation:

Maximum number of cavity-detection cycles	20
Probe radius	1.400
Maximum number of volume-refinement cycles	30
Minimum size of secondary grid	3
Grid for plot files	0.100
Primary grid spacing	0.100
Plot grid spacing	0.100

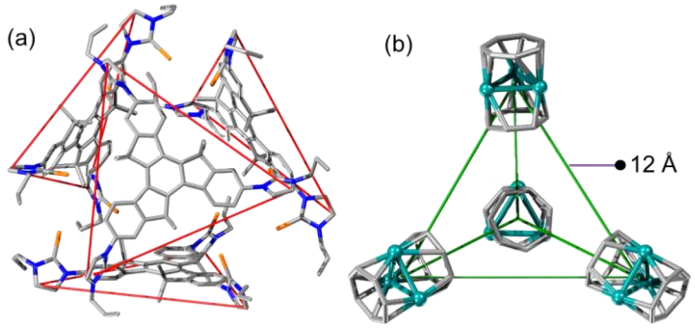

Figure S37. (a) View of the four organic ligands of the crystal structure of cage **2**. (b) View of the four tripalladium fragments within the crystal structure of cage **2**. Pd, teal sphere; Se, light orange; N, blue; C, gray; H, light red. Hydrogen atoms have been omitted for clarity (except for partial methyl units oriented inwards the cavity).

6. Hydrodynamic radius

Diffusion ordered spectroscopy (DOSY) NMR of **1** was performed on a Bruker 400 MHz NMR spectrometer using CD_3CN as the solvent at 298 K. The hydrodynamic radius R was calculated using the Stokes-Einstein equation: $D = (k_BT)/(6\pi\eta R)$, where D is the diffusion coefficient, k_B is the Boltzmann constant, T is absolute temperature, and η is the viscosity of CD_3CN at 298 K. The hydrodynamic radius R was calculated to be 15.8 Å. The value is very close to that observed in the solid state (16.0 Å).

7. Host–guest chemistry between cage 1 and C6 cyclic hydrocarbons

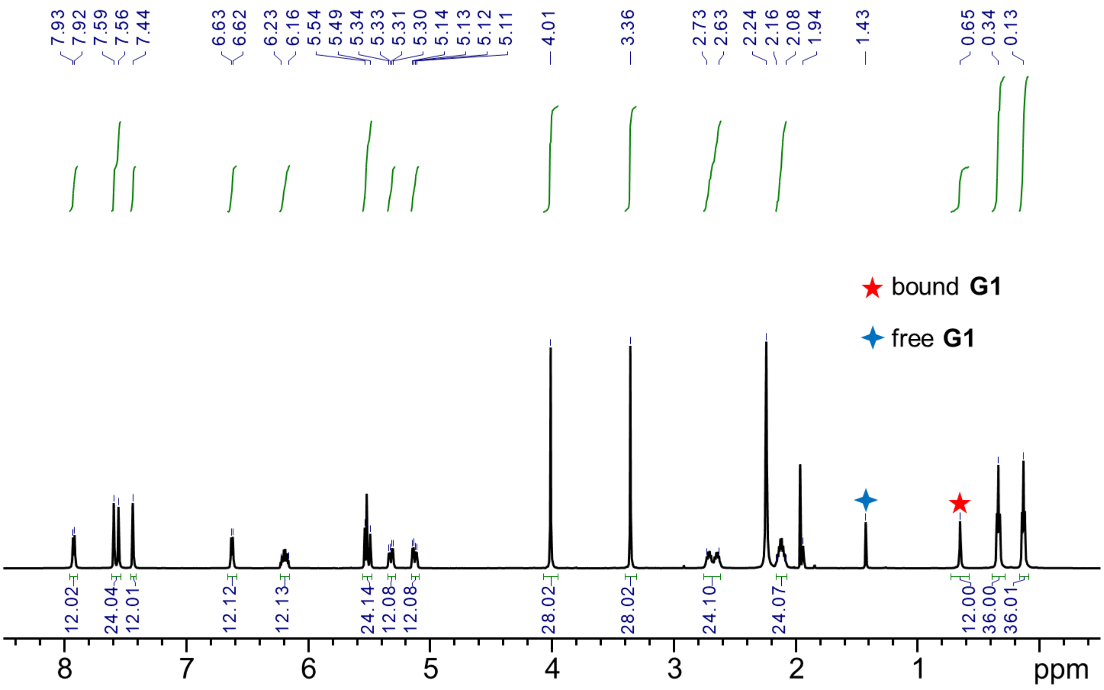

Figure S38. ^{1}H NMR spectrum (600 MHz, CD_3CN, 298 K) of cage **1** binding **G1**. (★) Bound guest. (✦) Free guest.

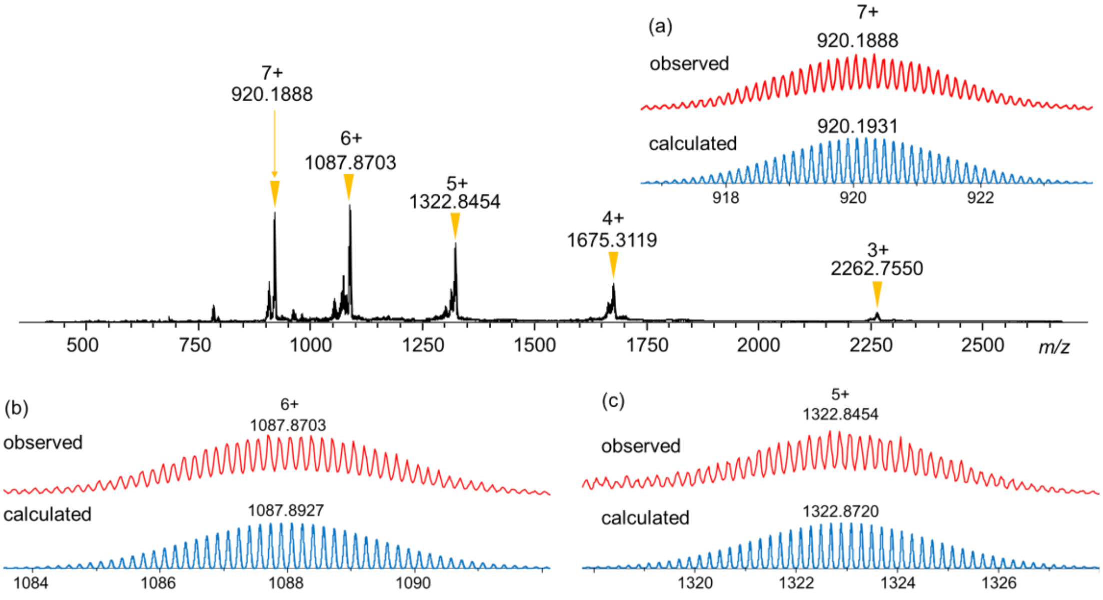

Figure S39. ESI mass spectrum (positive ions) of cage **1** binding **G1**. Calculated (blue) and experimental (red) isotope distribution (positive ions) of **G1⊂1**: (a) [**G1⊂1** – $7BF_4]^{7+}$; (b) [**G1⊂1** – $6BF_4]^{6+}$; (c) [**G1⊂1** – $5BF_4]^{5+}$.

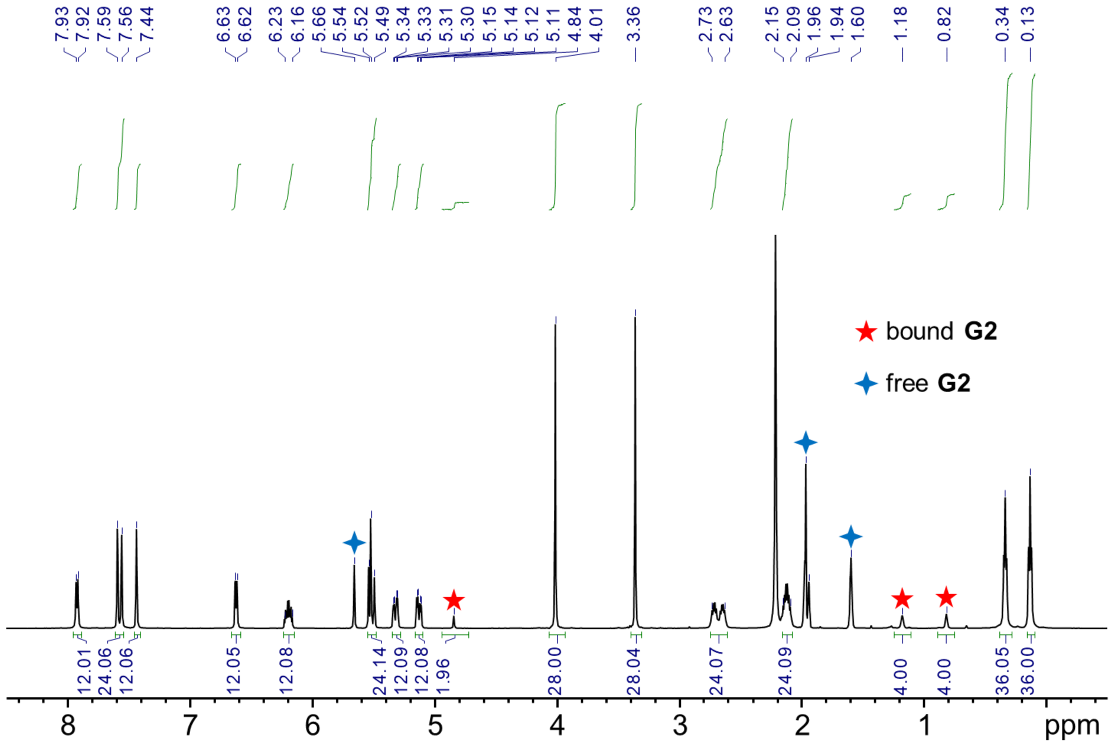

Figure S40. ^{1}H NMR spectrum (600 MHz, CD_3CN, 298 K) of cage **1** binding **G2**. (★) Bound guest. (✦) Free guest.

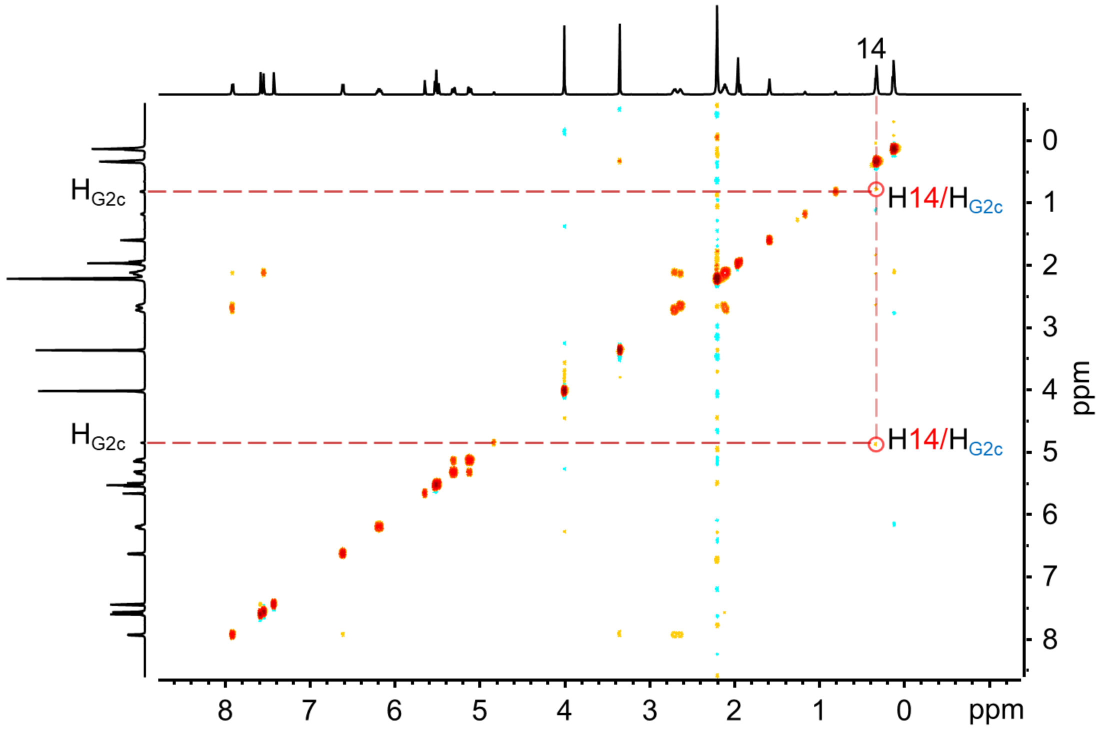

Figure S41. ^{1}H-^{1}H NOESY NMR spectrum (600 MHz, CD_3CN, 298 K) of cage **1** binding **G2**. Complexed species are represented by "c".

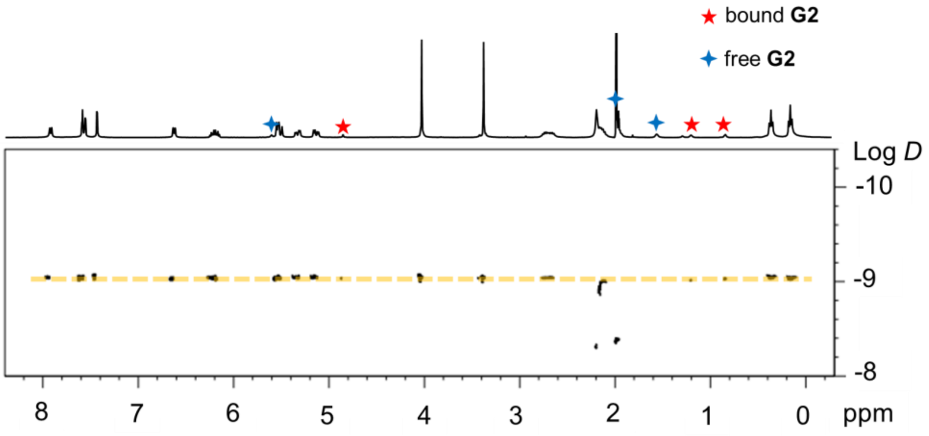

Figure S42. ^{1}H DOSY spectrum (600 MHz, CD_3CN, 298 K) of cage **1** binding **G2** (log D = −9.03). Protons of bound guest were represented by red five-pointed asterisks. (★) Bound guest. (✦) Free guest.

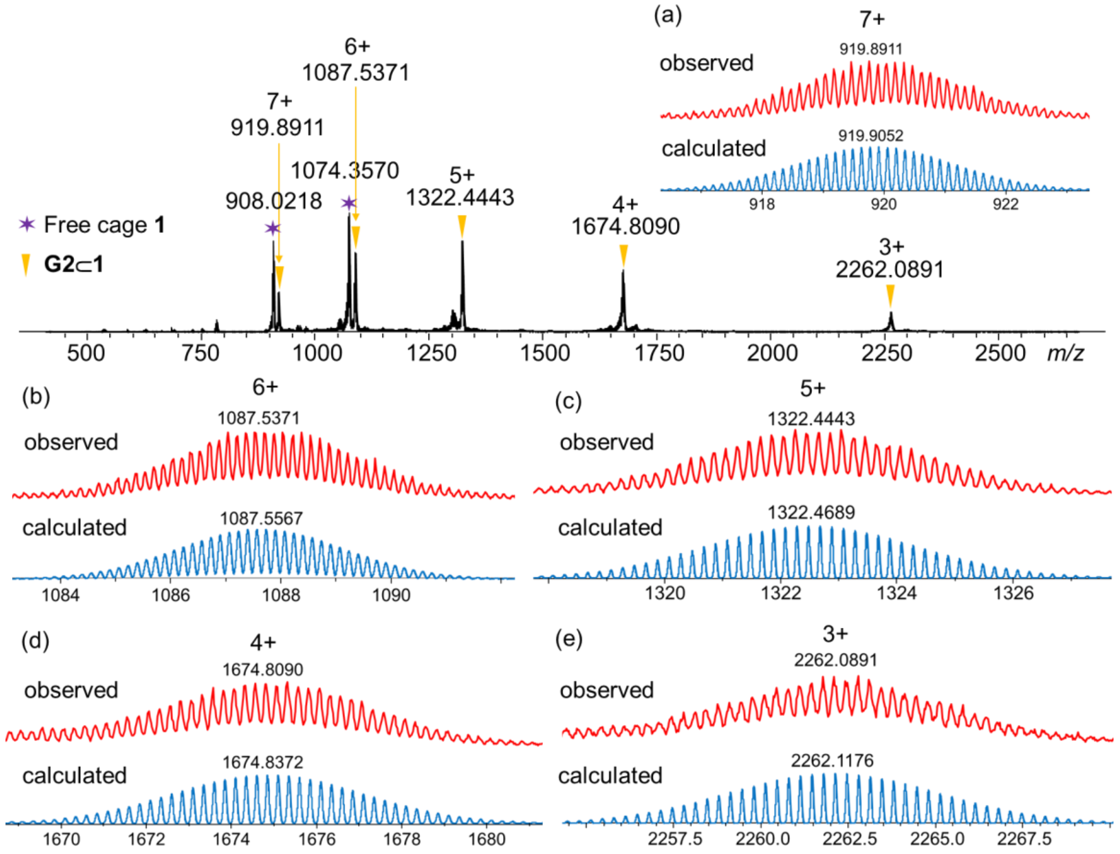

Figure S43. ESI mass spectrum (positive ions) of cage **1** binding **G2**. Calculated (blue) and experimental (red) isotope distribution (positive ions) of **G2⊂1**: (a) [**G2⊂1** – $7BF_4]^{7+}$; (b) [**G2⊂1** – $6BF_4]\}^{6+}$; (c) [**G2⊂1** – $5BF_4]^{5+}$; (d) [**G2⊂1** – $4BF_4]^{4+}$; (e) [**G2⊂1** – $3BF_4]^{3+}$. (▼) **G2⊂1**. (✶) Free cage **1**.

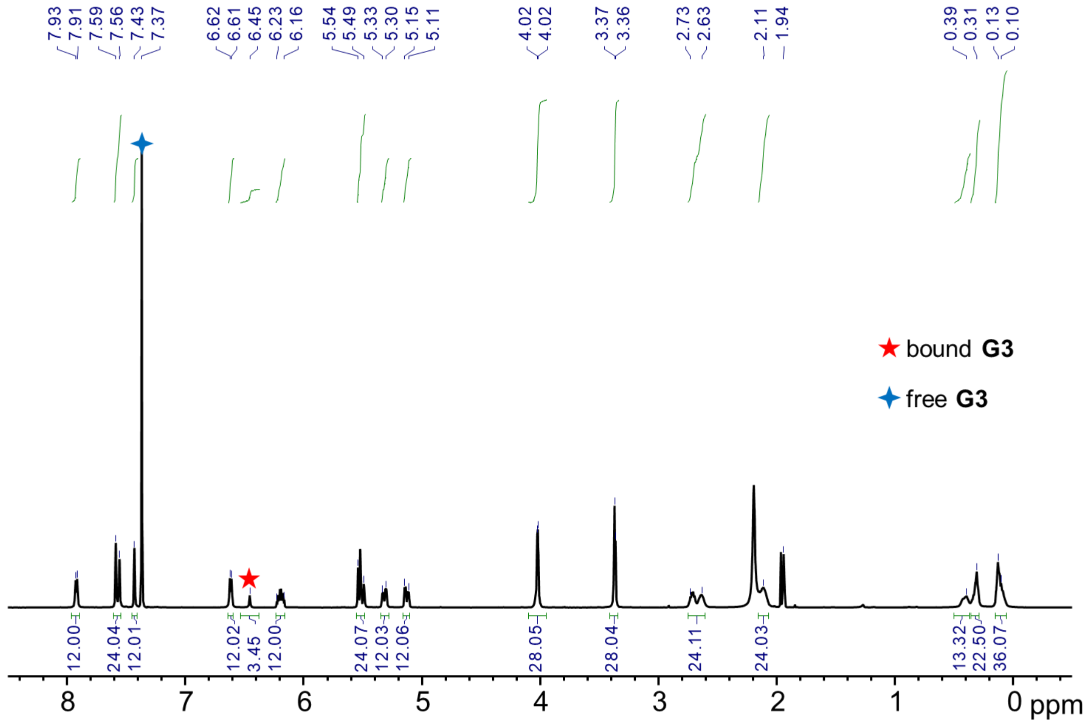

Figure S44. ^{1}H NMR spectrum (600 MHz, CD_3CN, 298 K) of cage **1** binding **G3**. (★) Bound guest. (✦) Free guest.

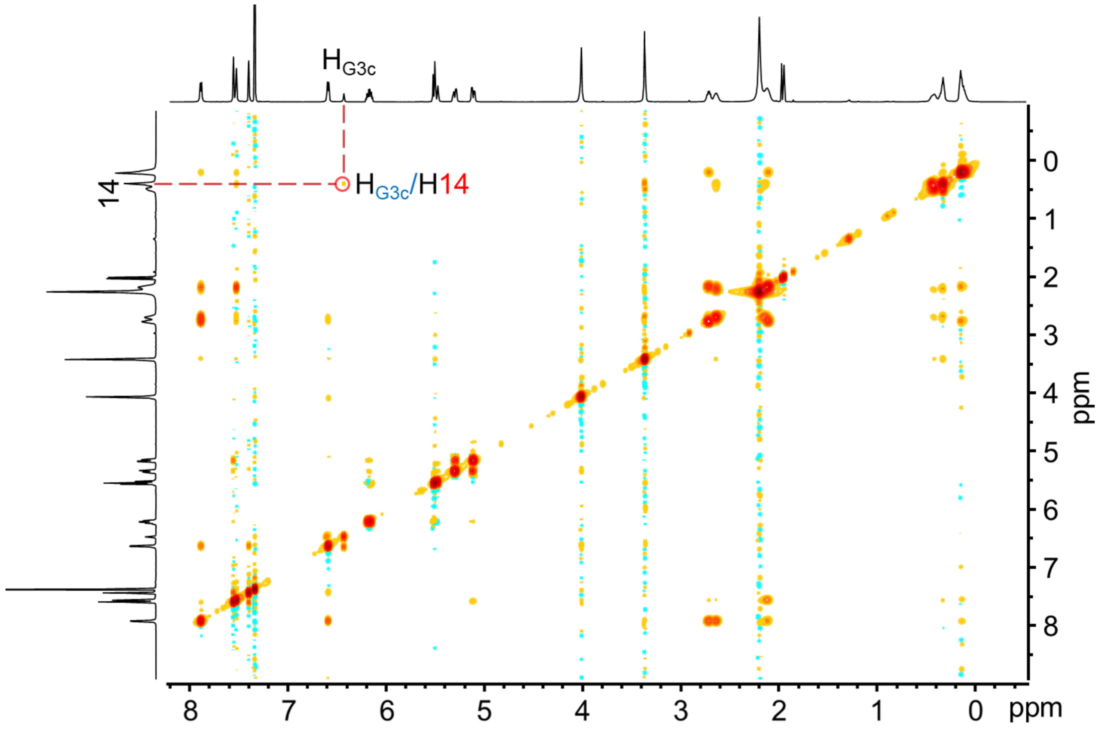

Figure S45. ^{1}H-^{1}H NOESY NMR spectrum (600 MHz, CD_3CN, 298 K) of cage **1** binding **G3**. Complexed species are represented by "c".

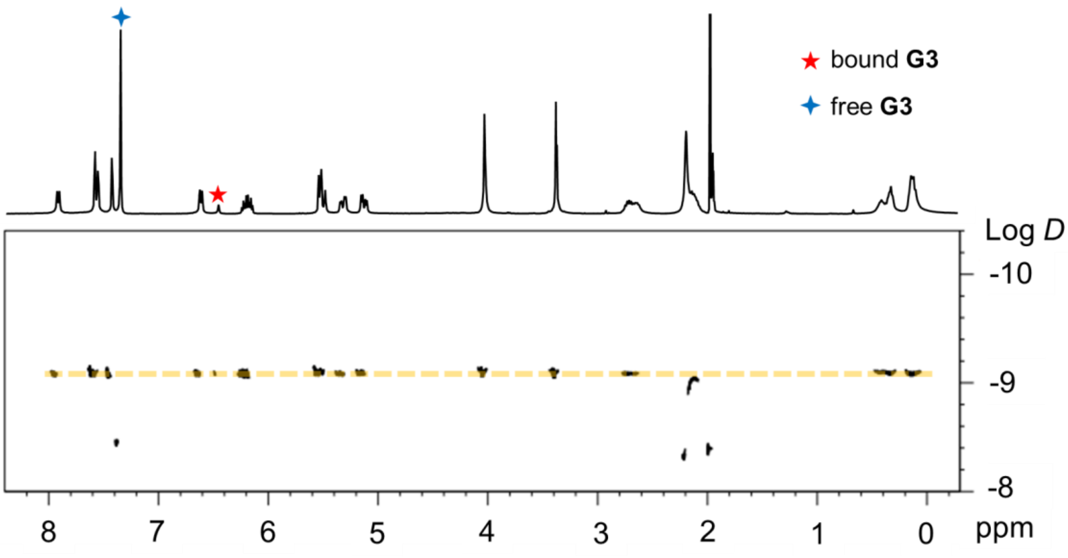

Figure S46. ^{1}H DOSY spectrum (CD_3CN, 600 MHz, 298 K) of cage **1** binding **G3** (log D = −9.13). Protons of bound guest were represented by red five-pointed asterisks. (★) Bound guest. (✦) Free guest.

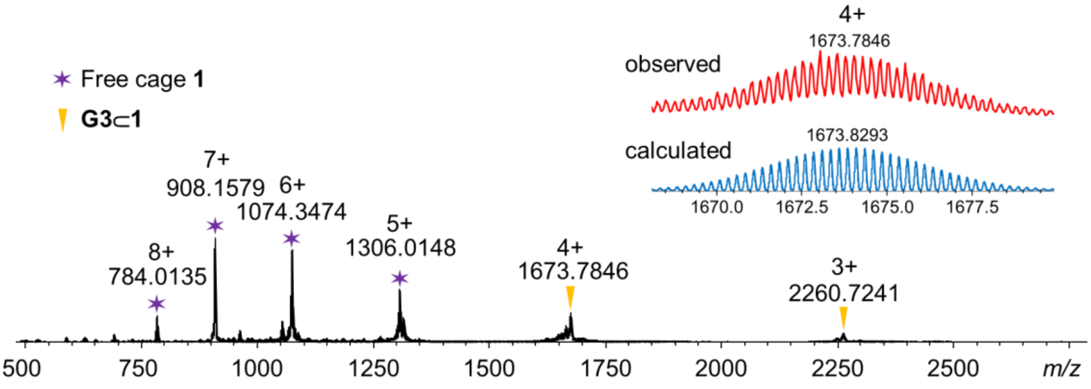

Figure S47. ESI mass spectrum (positive ions) of cage **1** binding **G3**. Calculated (blue) and experimental (red) isotope distribution (positive ions) of **G3⊂1**: [**G3⊂1** – $4BF_4]^{4+}$. (▼) **G3⊂1**. (✶) Free cage **1**.

8. Binding strength studies

The guest solution was titrated into the solution of free cage **1**, allowing the system to equilibrate overnight before obtaining each ^{1}H NMR spectrum. The relative integrations of the resonances corresponding to the free and bound host enabled the determination of the fraction of cage **1** binding to the guest as a function of the total amount of guest added. These data were fitted to the one-to-one binding model previously derived by Jonathan R. Nitschke's group [S4]. Due to the high binding strength of cage **1** to **G1**, the K_a value of **G1** may not be accurately obtained by the NMR titration test [S5,S6]. ITC experiment between cage **1** and **G1** was carried out by a VP-ITC (Malvern) at 298 K, and computer fitting of the data were performed using the VP-ITC analyze software.

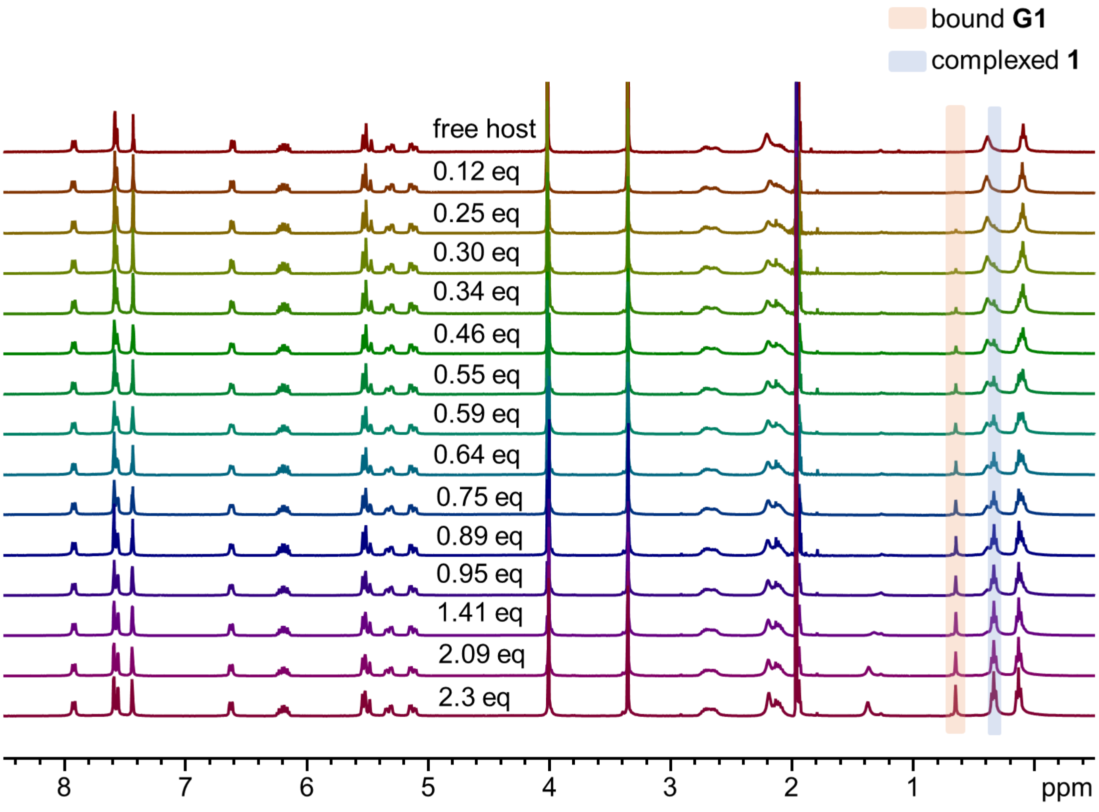

Figure S48. ^{1}H NMR titration (CD_3CN, 400 MHz, 298 K) of **G1** into a solution of cage **1** (10^{-2} M).

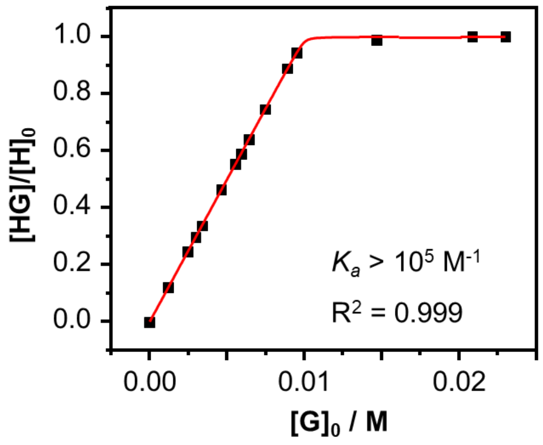

Figure S49. Data fitting for the titration of the slow exchanging **G1** into a solution of cage **1** (10^{-2} M). A binding constant (> 10^5 M^{-1}) is too large to be accurately determined from the ^{1}H NMR spectrum.

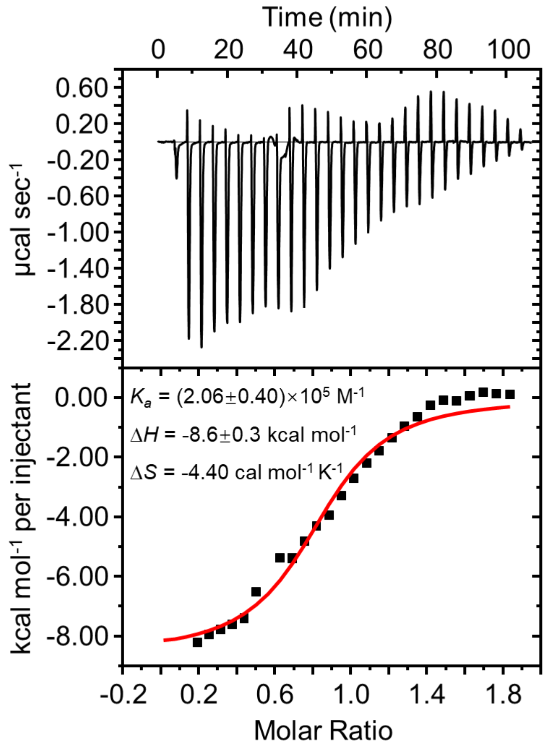

Figure S50. ITC titration plot and fitted curve (298 K, CH_3CN) obtained through titrations of **G1** (1 mM) into cage **1** (0.10 mM), which correspond well to a 1:1 binding stoichiometry. A binding constant of (2.06 ± 0.40) × 10^5 M^{-1} was determined for cage **1** binding **G1**, which in consistent well with the result from competitive guest binding experimental result (about 26 times of **G2**).

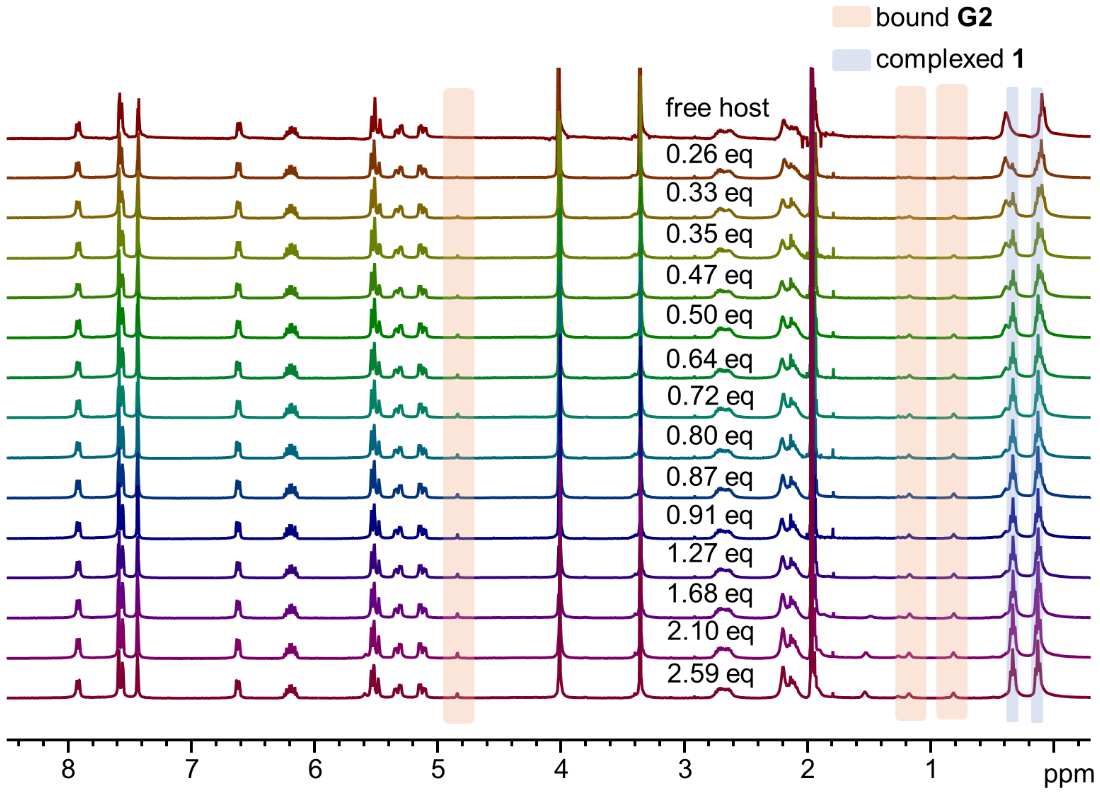

Figure S51. ^{1}H NMR titration (CD_3CN, 400 MHz, 298 K) of **G2** into a solution of cage **1** (10^{-2} M).

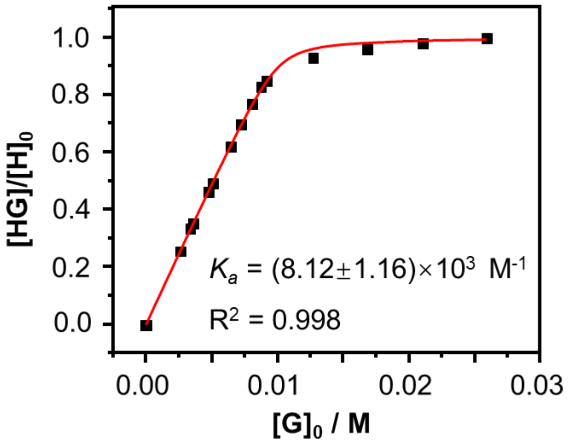

Figure S52. Data fitting for the NMR titration of the slow exchanging **G2** into a solution of cage **1** (10^{-2} M). A binding constant of (8.12 ± 1.16) × 10^{3} M^{-1} was determined for cage **1** binding **G2**.

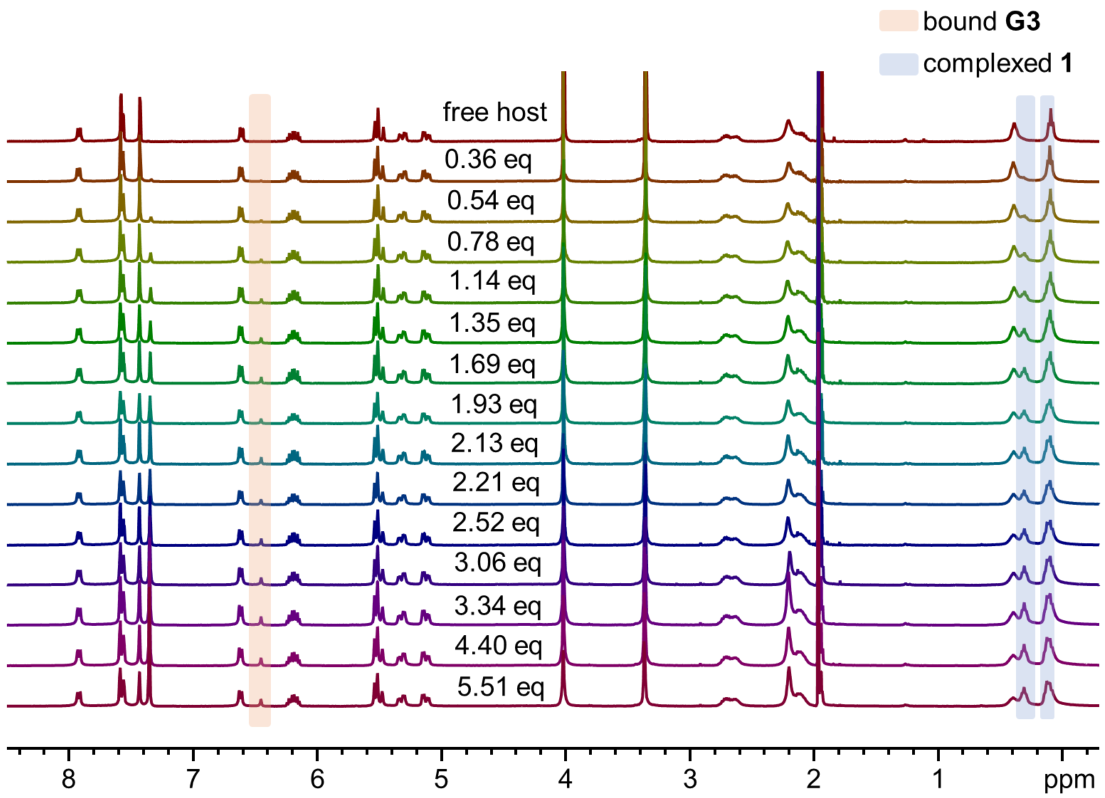

Figure S53. ^{1}H NMR titration (CD_3CN, 400 MHz, 298 K) of **G3** into a solution of cage **1** (10^{-2} M).

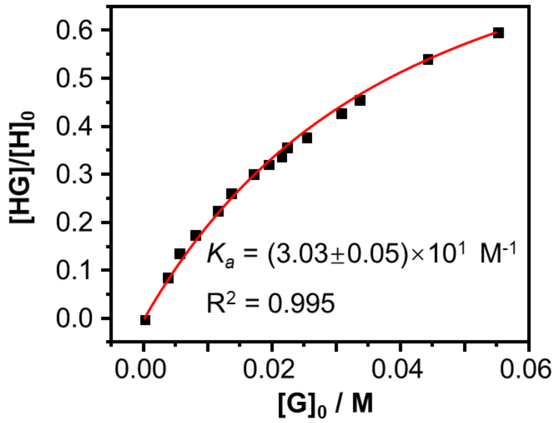

Figure S54. Data fitting for the NMR titration of the slow exchanging **G3** into a solution of cage **1** (10^{-2} M). A binding constant of $(3.03 \pm 0.05) \times 10^1$ M^{-1} was determined for cage **1** binding **G3**.

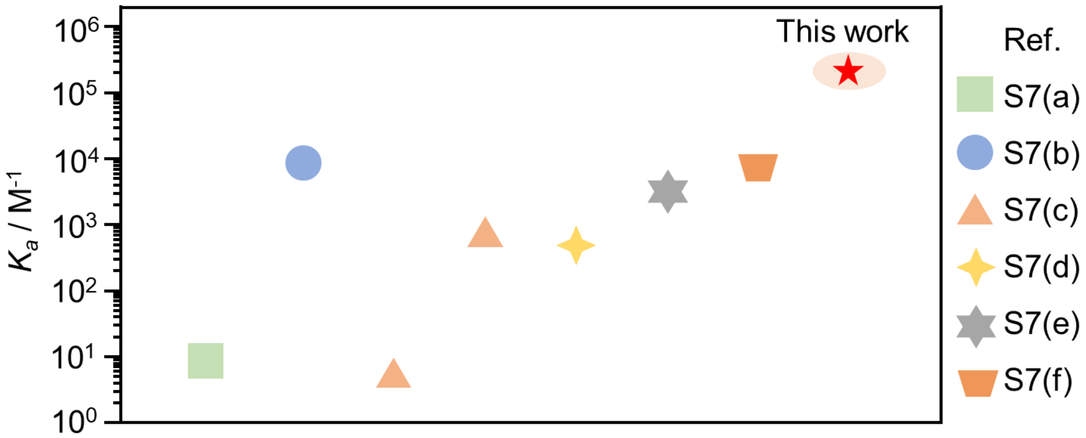

Figure S55. Comparison of binding constants of cage **1** and other reported coordination cages for cyclohexane [S7].

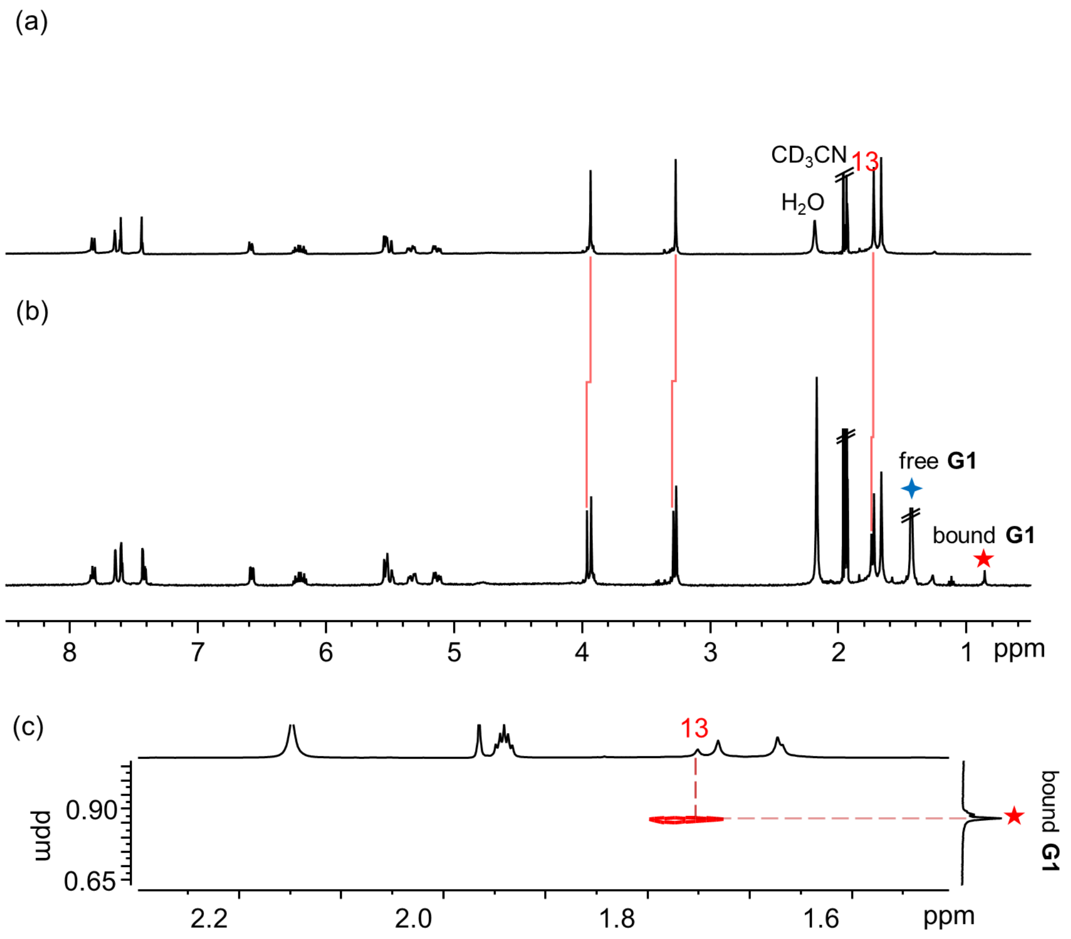

Figure S56. ^{1}H NMR spectra (CD_3CN, 400 MHz, 298 K) of (a) free cage **2** (10^{-3} M) and (b) cage **2** binding **G1**. (c) ^{1}H–^{1}H NOESY spectrum (CD_3CN, 600 MHz, 298 K) of the host−guest complex cage **1** binding **G1**. (★) Bound guest. (✦) Free guest.

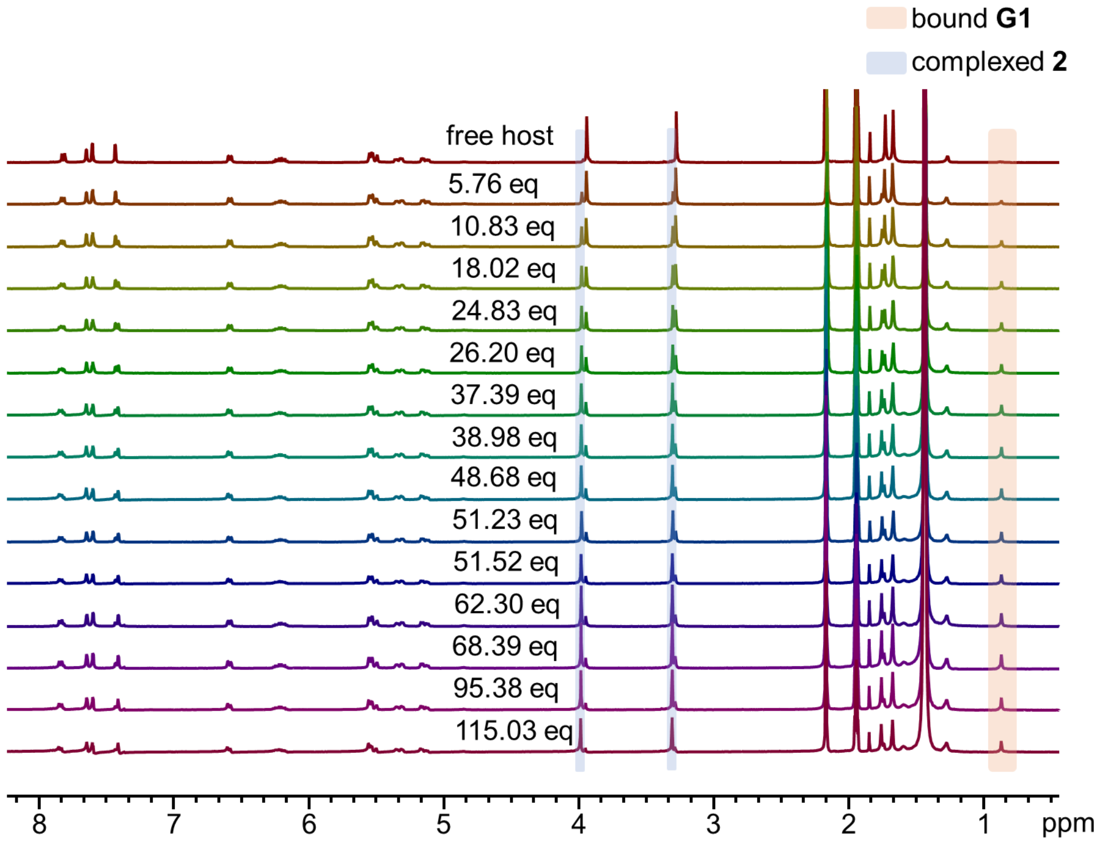

Figure S57. ^{1}H NMR titration (CD_3CN, 400 MHz, 298 K) of **G1** into a solution of cage **2** (3×10^{-3} M).

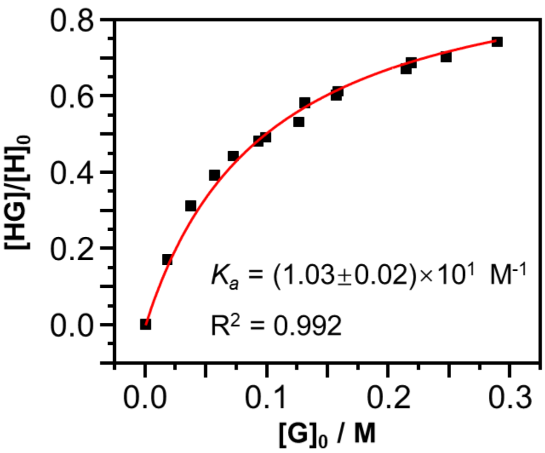

Figure S58. Data fitting for the NMR titration of the slow exchanging **G1** into a solution of cage **2** (3×10^{-3} M). A binding constant of $(1.03 \pm 0.02) \times 10^{1}$ M^{-1} was determined for cage **2** binding **G1**.

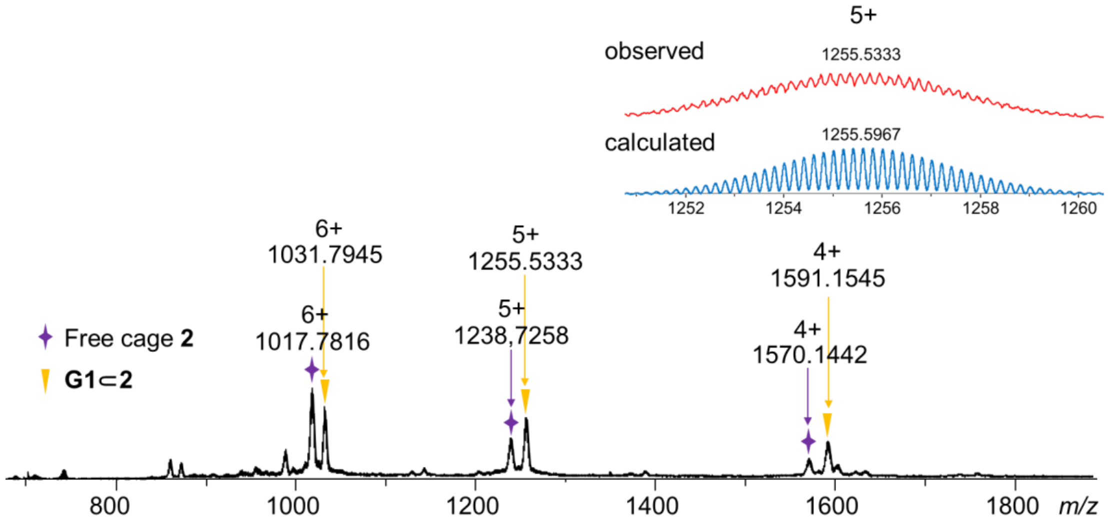

Figure S59. ESI mass spectrum (positive ions) of cage **2** binding **G1**. Calculated (blue) and experimental (red) isotope distribution (positive ions) of **G1⊂2**: [**G1⊂2** – $5BF_4]^{5+}$. (▼) **G1⊂2**. (✶) Free cage **2**.

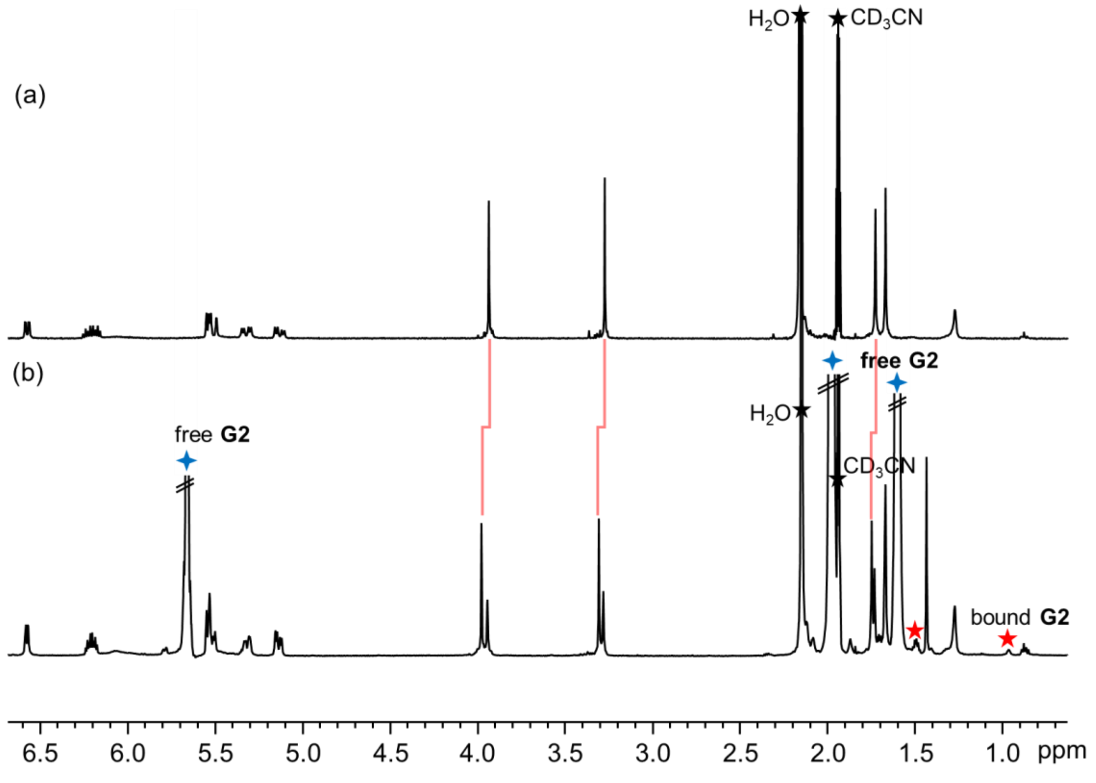

Figure S60. ^{1}H NMR spectra (CD_3CN, 400 MHz, 298 K) of (a) free cage **2** (10^{-3} M) and (b) cage **2** binding **G2**. (★) Bound guest. (✦) Free guest.

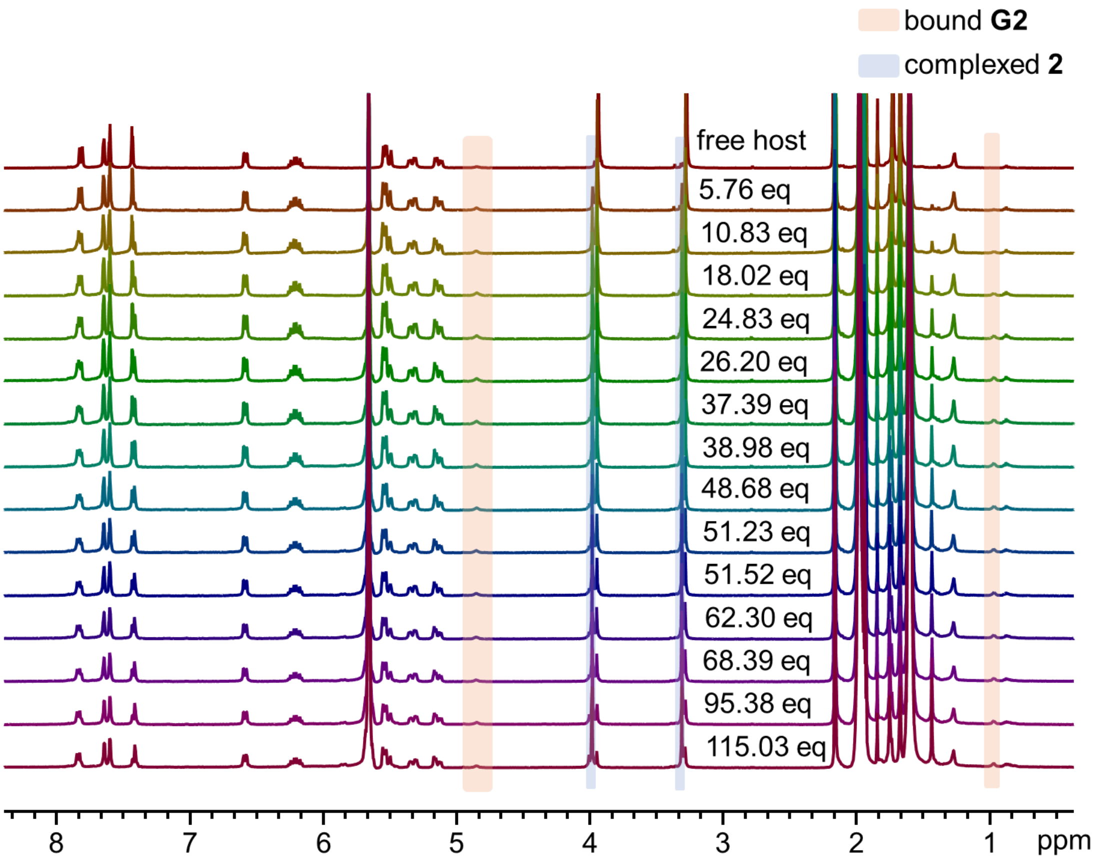

Figure S61. ^{1}H NMR titration (CD_3CN, 400 MHz, 298 K) of **G2** into a solution of cage **2** (3×10^{-3} M).

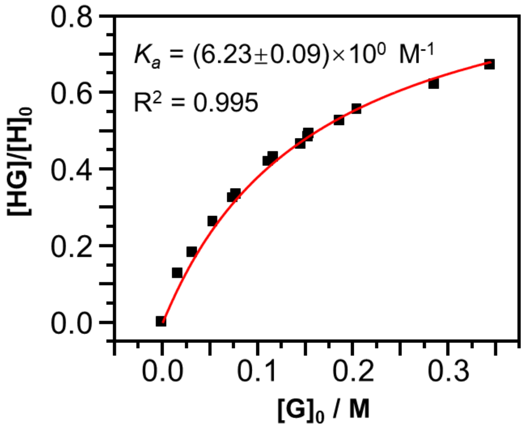

Figure S62. Data fitting for the NMR titration of the slow exchanging **G2** into a solution of cage **2** (3×10^{-3} M). A binding constant of $(6.23 \pm 0.09) \times 10^0$ M^{-1} was determined for cage **2** binding **G2**.

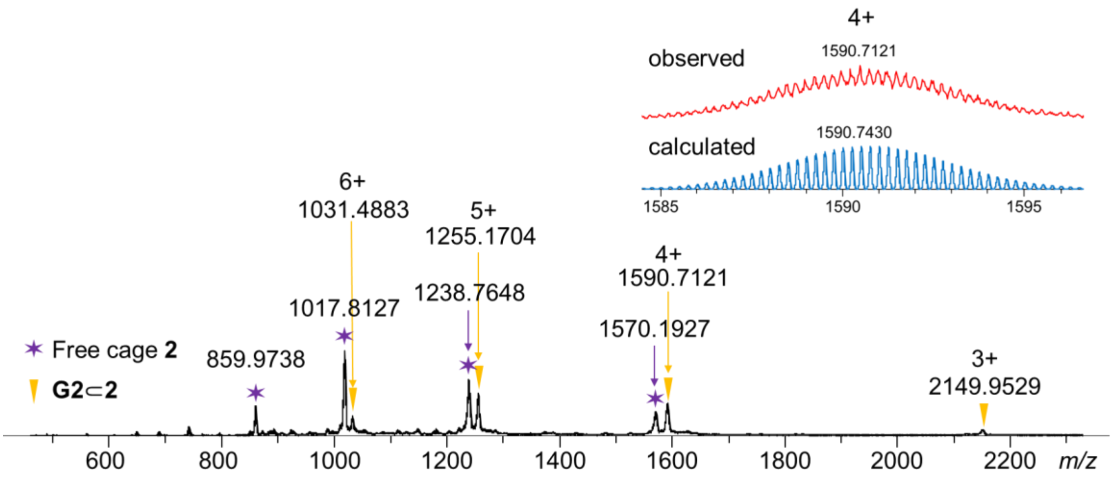

Figure S63. ESI mass spectrum (positive ions) of cage **2** binding **G2**. Calculated (blue) and experimental (red) isotope distribution (positive ions) of **G2⊂2**: [**G2⊂2** – $4BF_4]^{4+}$. (▼) **G2⊂2**. (✶) Free cage **2**.

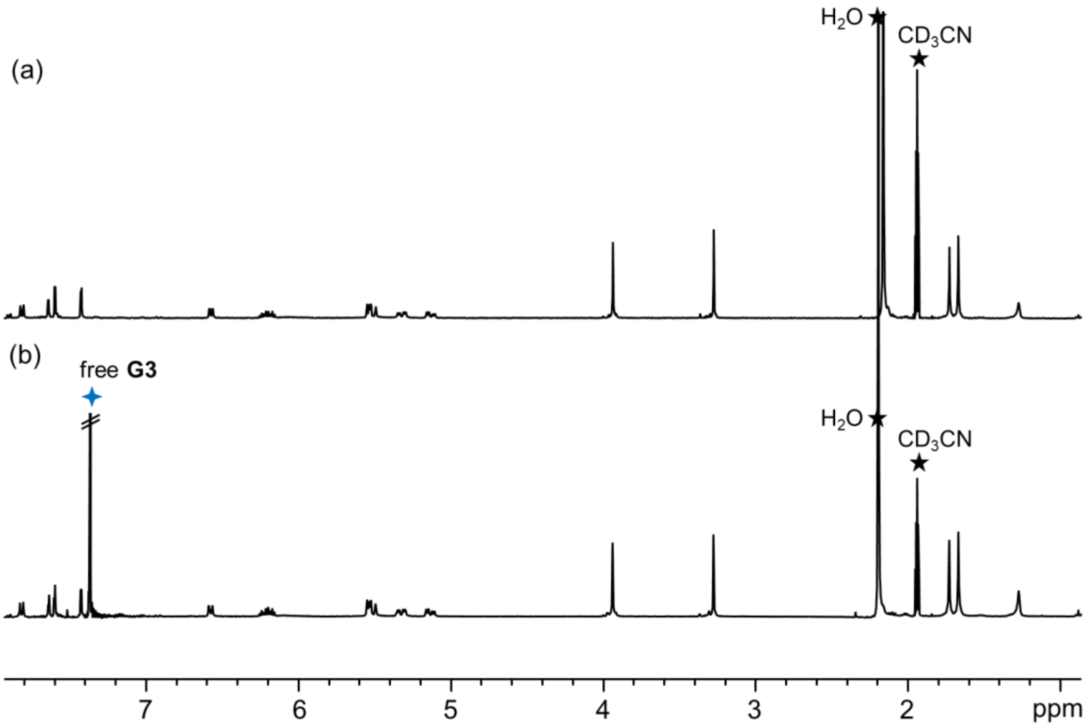

Figure S64. ^{1}H NMR spectra (CD_3CN, 400 MHz, 298 K) of (a) free cage **2** (10^{-3} M) and (b) cage **2** and **G3**. ^{1}H NMR integration indicated cage **2** cannot trap **G3** when excess **G3** (43 equiv) was added to a CD_3CN solution of **2**. (✦) Free guest.

9. Host–guest chemistry between cage 1 and other guests

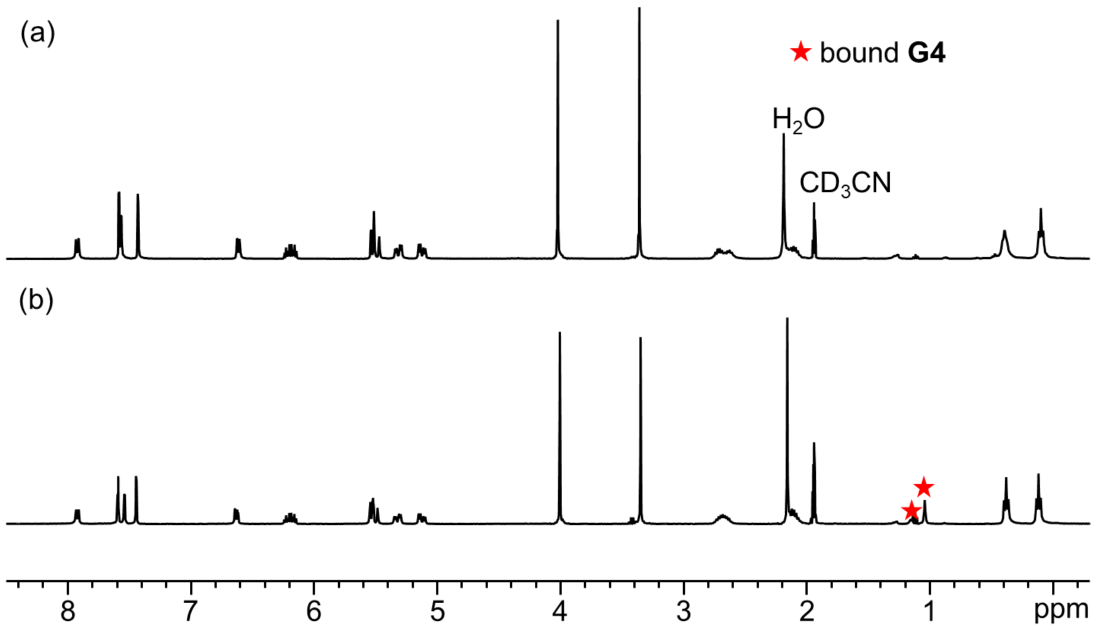

Figure S65. ^{1}H NMR spectra (CD_3CN, 400 MHz, 298 K) of (a) free cage **1** and (b) cage **1** binding **G4**.

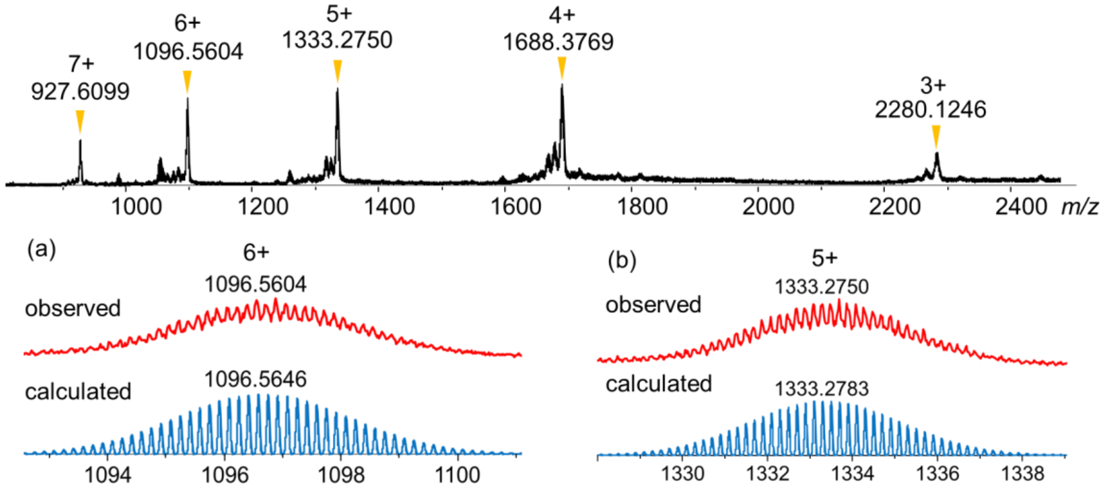

Figure S66. HR-ESI mass spectrum (positive ions) of cage **1** binding **G4**. Calculated (blue) and experimental (red) isotope distribution (positive ions) of **G4**⊂**1**: (a) {[**G4**⊂**1** – $6BF_4]^{6+}$; (b) [**G4**⊂**1** – $5BF_4]^{5+}$.

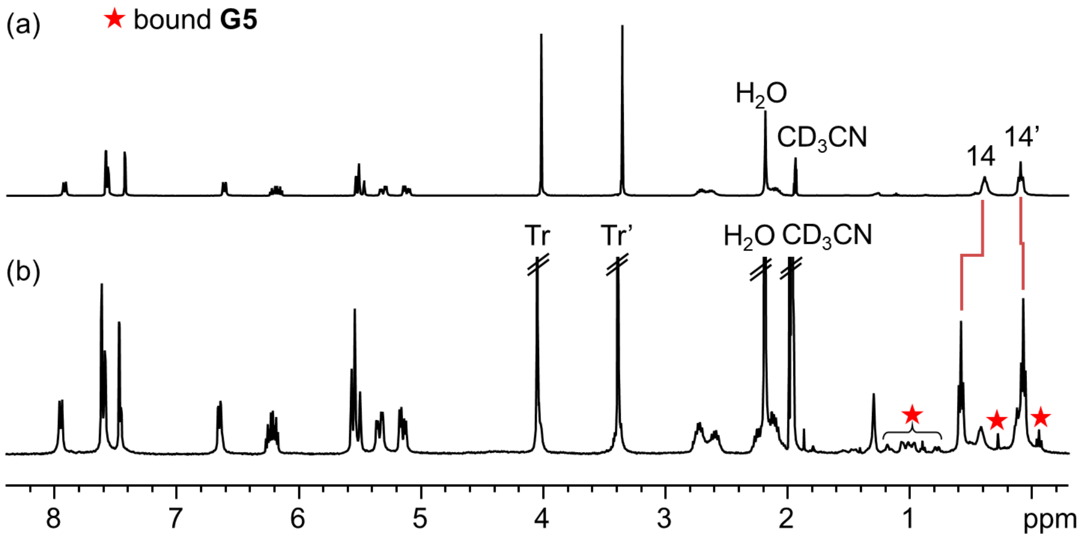

Figure S67. ^{1}H NMR spectra (CD_3CN, 400 MHz, 298 K) of (a) free cage **1** and (b) cage **1** binding **G5**.

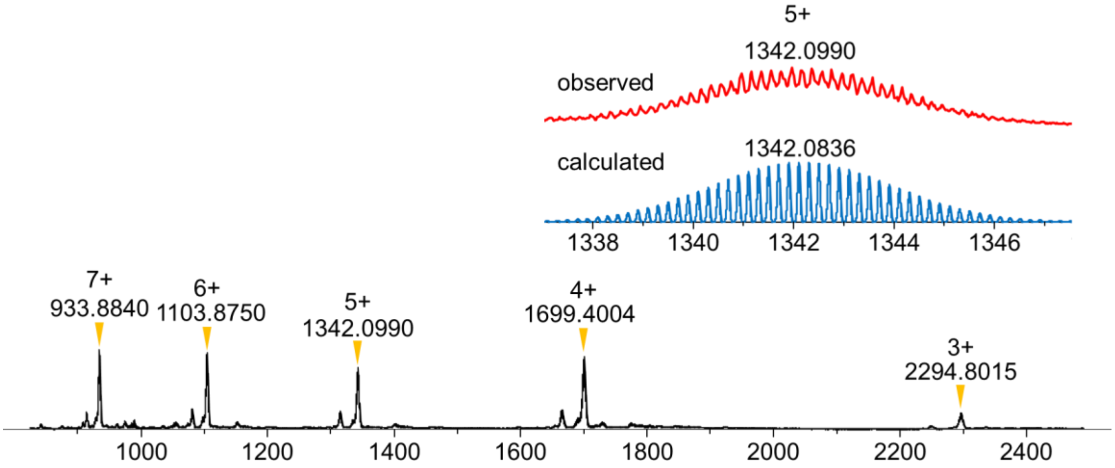

Figure S68. HR-ESI mass spectrum (positive ions) of cage **1** binding **G5**. Calculated (blue) and experimental (red) isotope distribution (positive ions) of **G5⊂1**: [**G5⊂1** – $5BF_4]^{5+}$.

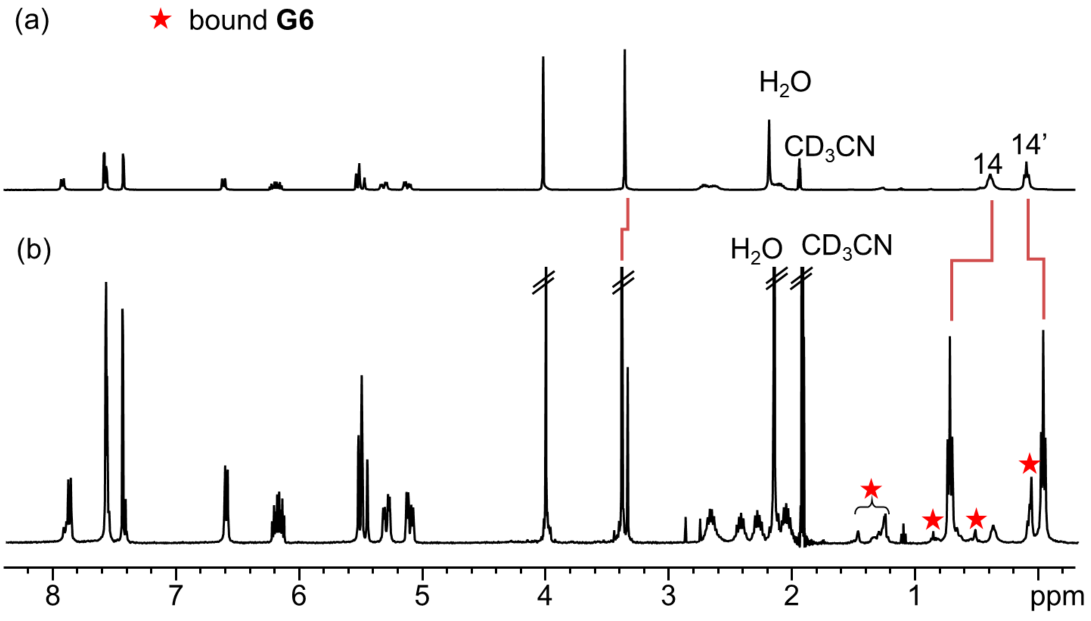

Figure S69. ^{1}H NMR spectra (CD_3CN, 400 MHz, 298 K) of (a) free cage **1** and (b) cage **1** binding **G6**.

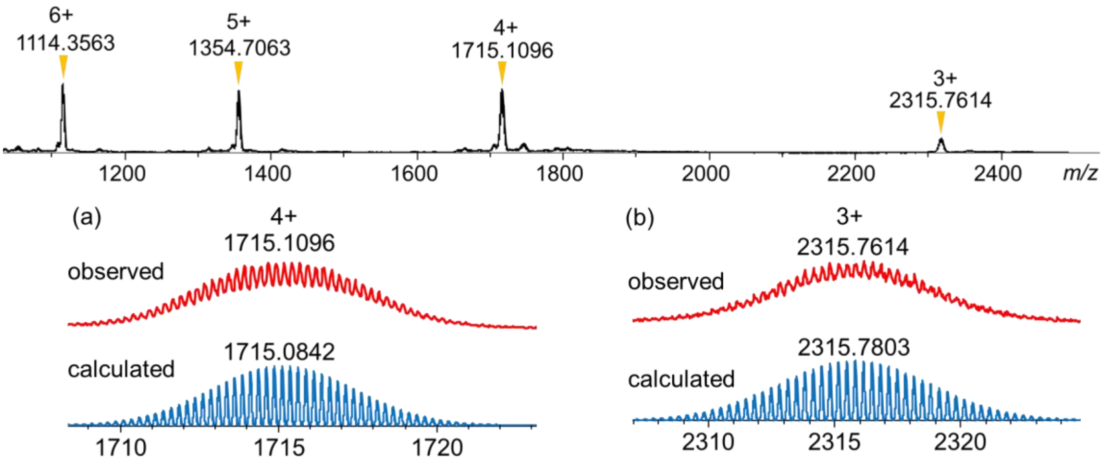

Figure S70. HR-ESI mass spectrum (positive ions) of cage **1** binding **G6**. Calculated (blue) and experimental (red) isotope distribution (positive ions) of **G6⊂1**: [**G6⊂1** – $4BF_4]^{4+}$; (b) [**G6⊂1** – $3BF_4]^{3+}$.

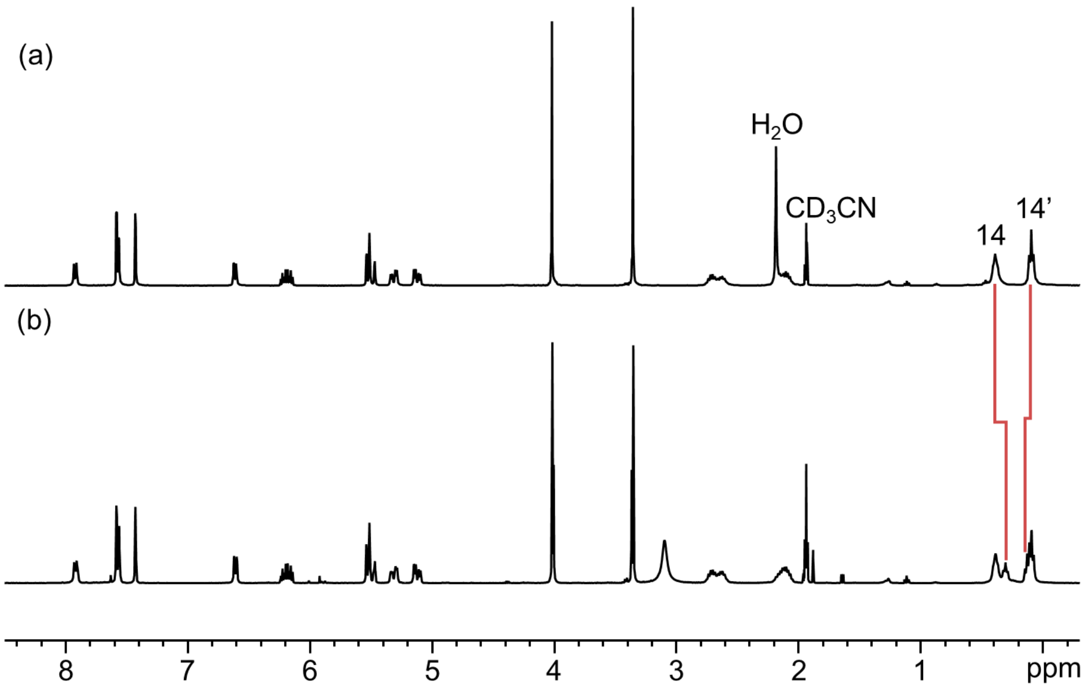

Figure S71. ^{1}H NMR spectra (CD_3CN, 400 MHz, 298 K) of (a) free cage **1** and (b) cage **1** binding **G7**.

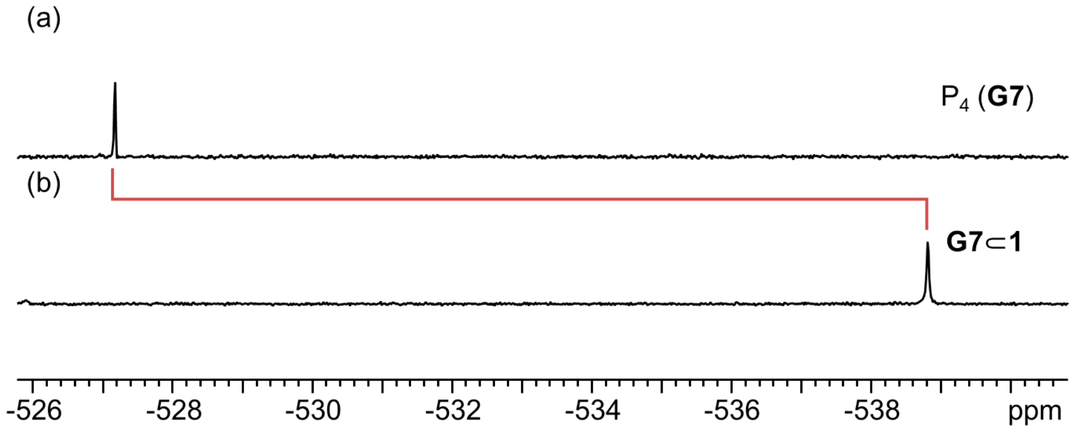

Figure S72. ^{31}P NMR spectra (CD_3CN, 162 MHz, 298 K) of (a) free **G7** (P_4) and (b) cage **1** binding **G7.**

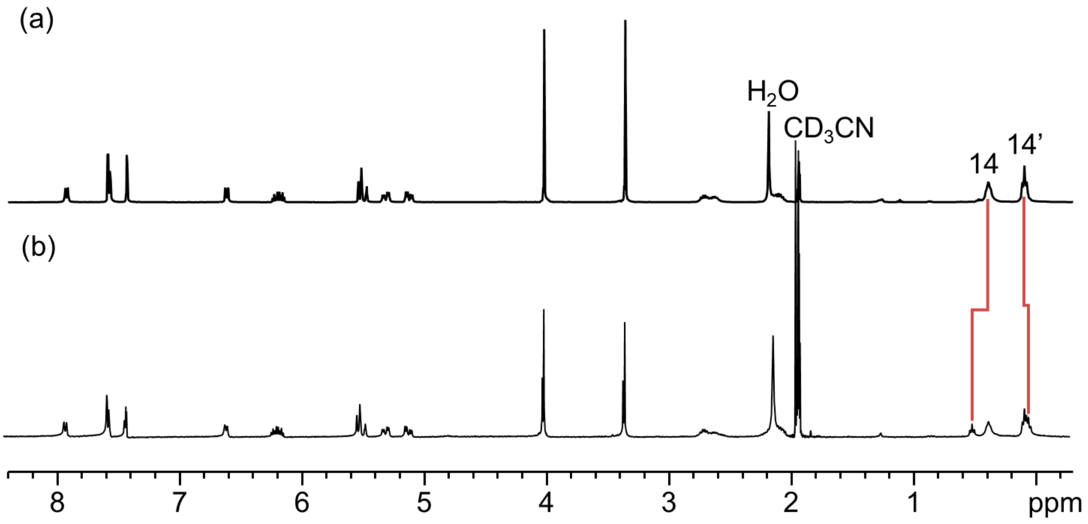

Figure S73. ^1H NMR spectra (CD_3CN, 400 MHz, 298 K) of (a) free cage **1** and (b) cage **1** binding **G8**.

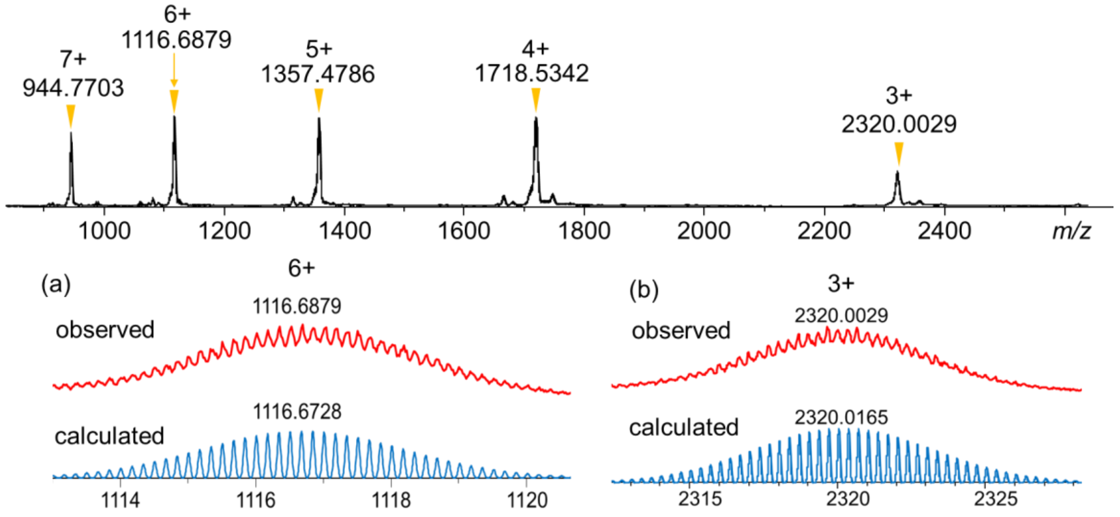

Figure S74. HR-ESI mass spectrum (positive ions) of cage **1** binding **G8**. Calculated (blue) and experimental (red) isotope distribution (positive ions) of **G8⊂1**: (a) [**G8⊂1** – $6BF_4]^{6+}$; (b) [**G8⊂1** – $3BF_4]^{3+}$.

10. X-ray crystallography

Single crystals of ligand **L1** and cage **1** for X-ray diffraction analysis have been obtained by slow diffusion of diethyl ether into a chloroform solution of ligand **L1**, and slow diffusion of isopropyl ether into a N,N-dimethylformamide solution of cage **1** at ambient temperature. Single crystals suitable for diffraction were obtained by the slow vapor diffusion of dioxane and tetrahydrofuran into a DMF solution of cage **2**. Single crystals of the host–guest complexes can be gained by the slow vapor diffusion of benzene into a DMF solution of **G4⊂1** or **G8⊂1**. Diffraction data was collected at 200–236 K with a Bruker APEX-II CCD diffractometer. Using the Olex2 program, the structures were solved with the Olex2.solve structure solution program using Charge Flipping and refined with the ShelXL refinement package using Least Squares minimization [S8-S10]. The structure graphics shown were generated made using the program Diamond. The crystal data of ligand **L1**, cage **1**, cage **2**, **G4⊂1**, **G8⊂1** and selected structural parameters are summarized in Tables S1–S5. Among them, despite many attempts at various types of radiation sources including Mo-*K*α radiation (λ = 0.71073 Å), Cu-*K*α radiation (λ =1.54178 Å) and Beamline BL17B (Synchrotron radiation: λ = 0.82654 Å) of National Facility for Protein Science at Shanghai Synchrotron Radiation Facility (SSRF), the single crystal data of the host–guest complexes **G4⊂1** and **G8⊂1** still exhibited the disorder of carbon atoms in the cycloheptatrienyl cationic rings due to the weakly diffraction at high angles. However, these phenomena did not affect our discussion on the overall architecture and ethyl units inside the cavity.

Table S1. Crystal data of ligand **L1**.

Empirical formula	$C_{59}H_{62}Cl_6N_6Se_3$
Formula weight	1304.72
Temperature/K	210
Crystal system	monoclinic
Space group	$P2_1/c$
a/Å	7.2661(3)
b/Å	21.7525(11)
c/Å	37.4417(19)
α/°	90
β/°	92.3910(10)
γ/°	90
Volume/Å^3	5912.7(5)
Z	4
ρ_{calc} g/cm^3	1.466
μ/mm^{-1}	2.181
F(000)	2648
Crystal size/mm^3	0.22 × 0.2 × 0.16
Radiation	Mo $K\alpha$ (λ = 0.71073)
2Θ range for data collection/ °	3.744 to 50.6
Index ranges	$-8 \le h \le 8$, $-26 \le k \le 26$, $-44 \le l \le 44$
Reflections collected	45734
Independent reflections	10756 [R_{int} = 0.0745, R_{sigma} = 0.0810]
Data/restraints/parameters	10756/122/735
Goodness-of-fit on F^2	1.033
Final R indexes [$I \ge 2\sigma(I)$]	R_1 = 0.0586, wR_2 = 0.1403
Final R indexes [all data]	R_1 = 0.1240, wR_2 = 0.1711
Largest diff. peak/hole / e Å^{-3}	0.75/-0.72
CCDC	2321636

Table S2. Crystal data of cage **1**.

Empirical formula	$C_{284}H_{296}N_{24}Pd_{12}Se_{12}$
Formula weight	6269.75
Temperature/K	236
Crystal system	trigonal
Space group	$R\bar{3}c$
a/Å	35.183(2)
b/Å	35.183(2)
c/Å	119.223(6)
α/°	90
β/°	90
γ/°	120
Volume/$Å^3$	127811(16)
Z	12
ρ_{calc} g/cm^3	0.977
μ/mm^{-1}	1.554
F(000)	37536
Crystal size/mm^3	0.23 × 0.22 × 0.21
Radiation	Mo *K*α (λ = 0.71073)
2Θ range for data collection/ °	3.552 to 50.048
Index ranges	-33 ≤ h ≤ 41, -41 ≤ k ≤ 41, -132 ≤ l ≤ 141
Reflections collected	538340
Independent reflections	25086 [R_{int} = 0.0800, R_{sigma} = 0.0321]
Data/restraints/parameters	25086/521/1098
Goodness-of-fit on F^2	1.061
Final *R* indexes [$I \geq 2\sigma(I)$]	R_1 = 0.0630, wR_2 = 0.2112
Final *R* indexes [all data]	R_1 = 0.0968, wR_2 = 0.2463
Largest diff. peak/hole / e $Å^{-3}$	0.73/-0.95

CCDC	2321637

Table S3. Crystal data of cage **2**.

Empirical formula	$C_{260}H_{248}N_{24}Pd_{12}Se_{12}B_6F_{24}$
Formula weight	6453.99
Temperature/K	200
Crystal system	monoclinic
Space group	*C2/c*
a/Å	48.614(11)
b/Å	21.882(5)
c/Å	37.692(14)
α/°	90
β/°	126.272(4)
γ/°	90
Volume/Å^3	32325(16)
Z	4
ρ_{calc} g/cm^3	1.326
μ/mm^{-1}	2.061
F(000)	12728.0
Crystal size/mm^3	0.21 × 0.20 × 0.18
Radiation	Mo *K*α (λ = 0.71073)
2Θ range for data collection/ °	4.264 to 49
Index ranges	-56 ≤ h ≤ 56, -25 ≤ k ≤ 25, -43 ≤ l ≤ 43
Reflections collected	201166
Independent reflections	26403 [R_{int} = 0.1059, R_{sigma} = 0.0892]
Data/restraints/parameters	26403/543/1510
Goodness-of-fit on F^2	1.161
Final *R* indexes [$I \geq 2\sigma(I)$]	R_1 = 0.1014, wR_2 = 0.3085
Final *R* indexes [all data]	R_1 = 0.1610, wR_2 = 0.3448
Largest diff. peak/hole / e Å^{-3}	1.68/-0.90

CCDC	2414845

Table S4. Crystal data of **G4⊂1**.

Empirical formula	$C_{286}H_{304}N_{24}Pd_{12}Se_{12}$
Formula weight	6301.83
Temperature/K	220.15
Crystal system	cubic
Space group	*I23*
a/Å	25.8484(18)
b/Å	25.8484(18)
c/Å	25.8484(18)
α/°	90
β/°	90
γ/°	90
Volume/$Å^3$	17270(4)
Z	2
ρ_{calc} g/cm^3	1.212
μ/mm^{-1}	1.918
F(000)	6296.0
Crystal size/mm^3	0.21 × 0.2 × 0.18
Radiation	Mo *K*α (λ = 0.71073)
2Θ range for data collection/ °	3.86 to 50.086
Index ranges	-30 ≤ h ≤ 30, -30 ≤ k ≤ 30, -30 ≤ l ≤ 30
Reflections collected	79828
Independent reflections	5078 [R_{int} = 0.0412, R_{sigma} = 0.0207]
Data/restraints/parameters	5078/261/245
Goodness-of-fit on F^2	1.024
Final *R* indexes [$I \geq 2\sigma(I)$]	R_1 = 0.0732, wR_2 = 0.2218
Final *R* indexes [all data]	R_1 = 0.1021, wR_2 = 0.2626
Largest diff. peak/hole / e $Å^{-3}$	0.71/-0.98

CCDC	2414847

Table S5. Crystal data of **G8⊂1**.

Empirical formula	$C_{280}H_{292}N_{24}Pd_{12}S_8Se_{12}$
Formula weight	6474.16
Temperature/K	213.15
Crystal system	cubic
Space group	I23
a/Å	25.689(3)
b/Å	25.689(3)
c/Å	25.689(3)
α/°	90
β/°	90
γ/°	90
Volume/$Å^3$	16953(6)
Z	2
ρ_{calc} g/cm^3	1.268
μ/mm^{-1}	2.003
F(000)	6456.0
Crystal size/mm^3	0.23 × 0.2 × 0.18
Radiation	Mo *K*α (λ = 0.71073)
2Θ range for data collection/ °	3.884 to 46.492
Index ranges	-28 ≤ h ≤ 23, -15 ≤ k ≤ 26, -28 ≤ l ≤ 20
Reflections collected	23205
Independent reflections	4034 [R_{int} = 0.0414, R_{sigma} = 0.0391]
Data/restraints/parameters	4034/248/247
Goodness-of-fit on F^2	1.246
Final *R* indexes [$I \geq 2\sigma(I)$]	R_1 = 0.0995, wR_2 = 0.2916
Final *R* indexes [all data]	R_1 = 0.1650, wR_2 = 0.3580
Largest diff. peak/hole / e Å^{-3}	0.71/-0.98

CCDC 2414846

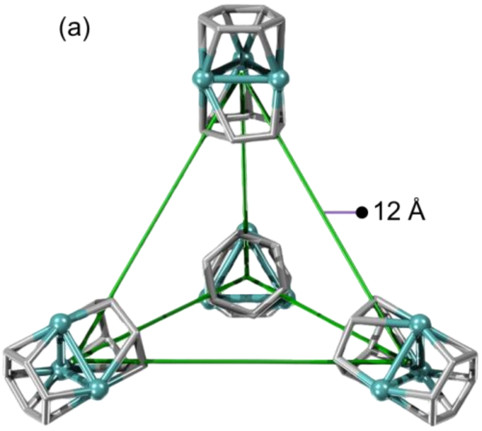

Figure S75. View of the four tripalladium fragments within the crystal structures of (a) **G4⊂1** and (b) **G8⊂1**.

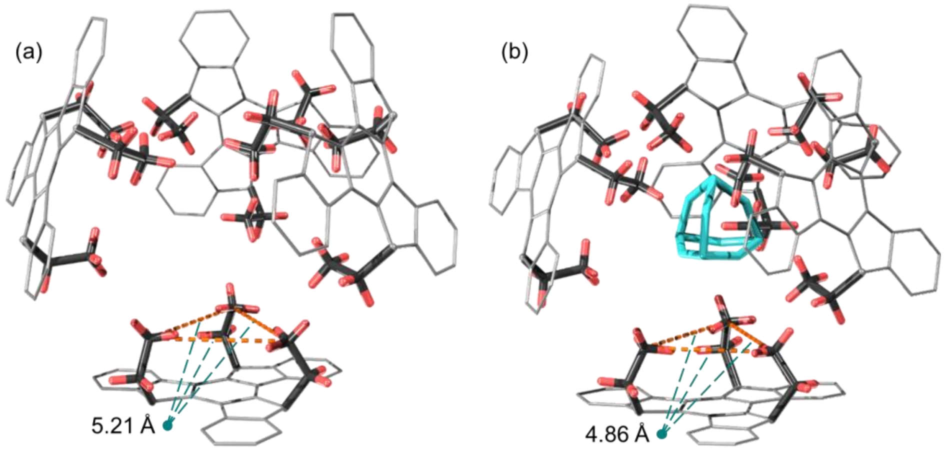

Figure S76. View involving partial aliphatic *endo*-functional units of a portion of the crystal structures of (a) cage **1** and (b) **G4⊂1** confirmed by single-crystal X-ray diffraction.

11. References

[S1] (a) Zhu JL. Zhang D, Ronson TK *et al.* A cavity-tailored metal–organic cage entraps gases selectively in solution and the amorphous solid state. *Angew Chem Int Ed* 2021; **60**: 11789–92. (b) Echeverri M, Gámez-Valenzuela S, González-Cano RC *et al.* Effect of the linkage position on the conjugation length of truxene-based porous polymers: implications for their sensing performance of nitroaromatics. *Chem Mater* 2019; **31**: 6971–8. (c) Wang X, Wang Y, Yang H *et al.* Assembled molecular face-rotating polyhedra to transfer chirality from two to three dimensions. *Nat Commun* 2016, **7**: 12469.

[S2] Murahashi T, Hashimoto Y, Chiyoda K *et al.* Reductive coupling of metal triangles in sandwich complexes. *J Am Chem Soc* 2008; **130**: 8586–7.

[S3] Kleywegt GJ, Jones TA Detection, delineation, measurement and display of cavities in macromolecular structures. *Acta Cryst* 1994; **D50**: 178–85.

[S4] Hristova YR, Smulders MMJ, Clegg, JK *et al.* Selective anion binding by a ‘‘chameleon’’ capsule with a dynamically reconfigurable exterior. *Chem Sci* 2011; **2**: 638–41.

[S5] Thordarson P. Determining association constants from titration experiments in supramolecular chemistry. *Chem Soc Rev* 2011; **40**: 1305–23.

[S6] Ulatowski F, Dąbrowa K, Bałakier T *et al.* Recognizing the limited applicability of Job plots in studying host–guest interactions in supramolecular chemistry. *J Org Chem* 2016; **81**: 1746–56.

[S7] (a) Wang LJ, Li X, Bai S *et al.* Self-assembly, structural transformation, and guest-binding properties of supramolecular assemblies with triangular metal–metal bonded units. *J Am Chem Soc* 2020; **142**: 2524–31. (b) Zheng J, von Krbek LKS, Ronson TK *et al.* Host spin-crossover thermodynamics indicate guest fit. *Angew Chem Int Ed* 2022; **61**: e202212634. (c) Xu L, Zhang D, Ronson TK *et al.* Improved acid resistance of a metal–organic cage enables cargo release and exchange between hosts. *Angew Chem Int Ed* 2020; **59**: 7435–8. (d) Castilla AM, Ronson TK, Nitschke JR. Sequence-dependent guest release triggered by

orthogonal chemical signals. *J Am Chem Soc* 2016; **138**: 2342–51. (e) Löffler S, Lübben J, Krause L *et al.* Triggered exchange of anionic for neutral guests inside a cationic coordination cage. *J Am Chem Soc* 2015; **137**: 1060–3. (f) Bolliger JL, Ronson TK, Ogawa M *et al.* Solvent effects upon guest binding and dynamics of a $Fe^{II}{}_4L_4$ cage. *J Am Chem Soc* 2014; **136**: 14545–53.

[S8] Dolomanov OV, Bourhis LJ, Gildea RJ *et al.* OLEX2: A complete structure solution, refinement andanalysis program. *J Appl Cryst* 2009; **42**: 339–41.

[S9] Bourhis LJ, Dolomanov OV, Gildea RJ *et al.* The anatomy of a comprehensive constrained, restrained refinement program for the modern computing environment - Olex2 dissected. *Acta Cryst* 2015; **A71**: 59–75.

[S10] Sheldrick GM. Crystal structure refinement with SHELXL. *Acta Cryst.* 2015; **C71**: 3–8.
